# Cell non-autonomous signaling through the conserved *C*. *elegans* glycoprotein hormone receptor FSHR-1 regulates cholinergic neurotransmission

**DOI:** 10.1371/journal.pgen.1011461

**Published:** 2024-11-19

**Authors:** Morgan Buckley, William P. Jacob, Letitia Bortey, Makenzi E. McClain, Alyssa L. Ritter, Amy Godfrey, Allyson S. Munneke, Shankar Ramachandran, Signe Kenis, Julie C. Kolnik, Sarah Olofsson, Milica Nenadovich, Tanner Kutoloski, Lillian Rademacher, Alexandra Alva, Olivia Heinecke, Ryan Adkins, Shums Parkar, Reesha Bhagat, Jaelin Lunato, Isabel Beets, Michael M. Francis, Jennifer R. Kowalski

**Affiliations:** 1 Department of Biological Sciences, Butler University, Indianapolis, Indiana, United States of America; 2 Department of Neurobiology, University of Massachusetts Chan School of Medicine, Worcester, Massachusetts, United States of America; 3 Neural Signaling and Circuit Plasticity Group, Department of Biology, KU Leuven, Leuven, Belgium; Brown University, UNITED STATES OF AMERICA

## Abstract

Modulation of neurotransmission is key for organismal responses to varying physiological contexts such as during infection, injury, or other stresses, as well as in learning and memory and for sensory adaptation. Roles for cell autonomous neuromodulatory mechanisms in these processes have been well described. The importance of cell non-autonomous pathways for inter-tissue signaling, such as gut-to-brain or glia-to-neuron, has emerged more recently, but the cellular mechanisms mediating such regulation remain comparatively unexplored. Glycoproteins and their G protein-coupled receptors (GPCRs) are well-established orchestrators of multi-tissue signaling events that govern diverse physiological processes through both cell-autonomous and cell non-autonomous regulation. Here, we show that follicle stimulating hormone receptor, FSHR-1, the sole *Caenorhabditis elegans* ortholog of mammalian glycoprotein hormone GPCRs, is important for cell non-autonomous modulation of synaptic transmission. Inhibition of *fshr-1* expression reduces muscle contraction and leads to synaptic vesicle accumulation in cholinergic motor neurons. The neuromuscular and locomotor defects in *fshr-1* loss-of-function mutants are associated with an underlying accumulation of synaptic vesicles, build-up of the synaptic vesicle priming factor UNC-10/RIM, and decreased synaptic vesicle release from cholinergic motor neurons. Restoration of FSHR-1 to the intestine is sufficient to restore neuromuscular activity and synaptic vesicle localization to *fshr-1-*deficient animals. Intestine-specific knockdown of FSHR-1 reduces neuromuscular function, indicating FSHR-1 is both necessary and sufficient in the intestine for its neuromuscular effects. Re-expression of FSHR-1 in other sites of endogenous expression, including glial cells and neurons, also restored some neuromuscular deficits, indicating potential cross-tissue regulation from these tissues as well. Genetic interaction studies provide evidence that downstream effectors *gsa-1*/*G*α_*S*_, *acy-1*/adenylyl cyclase and *sphk-1/*sphingosine kinase and glycoprotein hormone subunit orthologs, GPLA-1/GPA2 and GPLB-1/GPB5, are important for intestinal FSHR-1 modulation of the NMJ. Together, our results demonstrate that FSHR-1 modulation directs inter-tissue signaling systems, which promote synaptic vesicle release at neuromuscular synapses.

## Author summary

Communication to the nervous system from other tissues, such as gut-to-brain signaling, helps to coordinate organismal responses to a variety of stresses, including infection, injury, and aging, as well as modulates learning, memory, and sensorimotor function. How this inter-tissue regulation of neurons occurs is not fully understood. Here we describe a role for a conserved glycoprotein hormone receptor, FSHR-1, in regulating neuromuscular signaling necessary for motility in *C. elegans*, a model roundworm that shares high genetic and nervous system conservation with humans. Our results indicate that FSHR-1 can act in the intestine, as well as in several other tissues, to promote signaling from excitatory motor neurons and muscle excitation. We further show FSHR-1 is specifically required in the intestine, where it works in conjunction with two known glycoprotein ligands and several known effector molecules, to regulate neuromuscular function. As related mammalian glycoprotein receptors are implicated in neurological and neurodegenerative conditions, our work is relevant for understanding human nervous system function and the impacts of neurological disease.

## Introduction

Decades of research have yielded tremendous insights into mechanisms by which signaling within pre- and post-synaptic neurons controls the amount and timing of neurotransmission at their cognate synapses; however, recent data from a diversity of systems has revealed that complex cell non-autonomous pathways also modulate synaptic activity. Cross-tissue signaling, including gut-brain, glial-neuronal, and inter-neuronal, is essential for nervous system function and organismal survival, particularly in the face of physiological stressors [1–5]. Gut-brain crosstalk, for example, occurs across phylogeny via neural and endocrine mechanisms to promote anti-bacterial effects and homeostatic organism-level protection [2,5,6]. Likewise, release of gliotransmitters from astrocytes can impact both short- and long-term plasticity at neuronal synapses, and cytokine and neurotrophic factor signaling from microglia affects synapse survival. These and other types of glial-neuronal communication are impacted by physiological circumstances including stress, reproduction, and homeostatic signals [7–9]. Finally, neuropeptide, lipid, and neurohormone signals released from one neuron type in response to a variety of internal and external states can impact transmission at both neighboring and more distant synapses [10,11]. Nevertheless, although inter-tissue signaling has been widely demonstrated and its effects on neuronal function are clear, the molecular players involved in these regulatory pathways remain largely unexplored.

G protein-coupled receptors (GPCRs) are a large class of seven-pass transmembrane proteins that can regulate multiple aspects of neuronal signaling and are involved in coordinating multi-tissue responses to diverse stimuli [12–16]. GPCRs are expressed in most tissues and can respond to multiple different cues and/or activate multiple distinct responses upon binding different ligands [17–19]. Functions for the more than 800 GPCRs encoded in the human genome include roles as receptors for neurotransmitters, neuropeptides, hormones, lipids, and other molecules [12,14,20,21]. Upon ligand binding, conformational changes in the activated receptor are transmitted to an associated heterotrimeric G protein [22]. The α subunit of the G protein exchanges GDP for GTP, which causes dissociation of the β and γ subunits and subsequent activation of any of a number of downstream signaling pathways, including production of second messengers such as cyclic adenosine monophosphate (cAMP), diacylglycerol (DAG), and Ca^2+^, that ultimately lead to changes in protein activity and/or gene expression [23].

GPCRs can exert their effects on neuronal signaling either cell autonomously, directing effects within the cells in which the GPCR itself is found, or cell non-autonomously, initiating signals that act in a different cell type. Cell autonomous functions of metabotropic glutamate, GABA_B_, and acetylcholine GPCRs include their roles as autoreceptors on presynaptic neurons, where they regulate synaptic transmission and form intrasynaptic feedback loops [14]. Additional neuronal GPCRs serve as receptors for neuropeptides and other neuroendocrine molecules, which influence synaptic transmission through effects on presynaptic protein function [24]. Examples of cell non-autonomous activities of GPCRs are increasingly described and have been documented in both neuronal and non-neuronal contexts. During zebrafish heart development, the Aplnr GPCR appears to direct the migration of embryonic cardiac progenitor cells (CPC) by acting in surrounding niche cells to activate a non-canonical signaling pathway. Activation of this pathway causes the release of one or more extracellular factors that initiate gene expression changes leading to CPC migration [25]. Likewise, in response to microbial infection, the DOP-4 dopamine receptor acts in ASG neurons in *C*. *elegans* to signal for the neuronal release of an as yet unidentified neuroendocrine molecule that acts on intestinal cells to initiate intestinal p38/MAP kinase signaling and immune-related gene expression [26]. Despite these examples, given the complexity of GPCR signaling networks and responses in the nervous system and beyond, as well the vast numbers of GPCRs found across phylogeny, a complete picture of intra- and inter-tissue signaling networks utilized by many GPCRs to influence nervous system function has yet to be fully elucidated.

The GPCR FSHR-1 is the sole *C*. *elegans* ortholog of vertebrate glycoprotein hormone receptors, belonging to the leucine-rich repeat-containing GPCR (LGRs) family, including the follicle-stimulating hormone receptor (FSHR), luteinizing hormone receptor (LHR), and thyroid-stimulating hormone receptor (TSHR), which are involved in regulating gonad differentiation and function, as well as energy homeostasis and development/metamorphosis in vertebrates [27–31]. These receptors are also expressed in the mammalian nervous system and other non-gonadal mammalian tissues [32–34]. Both LHR and TSHR have been implicated in controlling neuronal functions, with LHR signaling involved in learning and memory [34–37] and changes in both LH and TSH/TSHR levels linked to ADHD and Alzheimer’s Disease [38–42]. Expression of FSHR, and its ligand FSH, have been found in mammalian hippocampus neurons, cortex, and spinal cord tissue [32,43,44]. Although the functions of FSHR signaling in neuronal locations are not yet clear, recent studies found *Fshr* deficiency causes depressive and affective disorder behaviors in mice [45,46]. Additional work demonstrated a role for FSH as a driver of amyloid-β and Tau deposition and associated cognitive impairment—defects that could be reduced by inhibition of FSH/FSHR signaling [44,47], suggesting further unexplored roles in the nervous system.

*C*. *elegans* FSHR-1 is known to be expressed in multiple tissues, including a subset of head neurons and glia, as well as the intestine, pharynx, and vulva [27,48,49], and regulates a variety of physiological processes, many of which interface with the nervous system [48–50]. These processes include innate immunity, oxidative and other stress responses, germline differentiation, body size regulation, lipid homeostasis, and stress-induced organismal death, or phenoptosis [27,48,51–56]. FSHR-1 acts as a cell non-autonomous endocrine regulator in several of these processes, including oxidative stress responses, germline differentiation, freeze-thaw induced phenoptosis, and body size [27,48,51]. Thus, *C*. *elegans* FSHR-1 represents an ideal ortholog through which to explore additional cell autonomous and non-autonomous neuronal and neuro-regulatory functions of the LGR family of receptors.

Prior RNA interference (RNAi) screens in *C*. *elegans* implicated FSHR-1 in the regulation of synaptic vesicle exocytosis and neuromuscular function. *fshr-1* knockdown animals and loss-of-function genetic mutants are resistant to paralysis induced by the acetylcholinesterase inhibitor aldicarb [50] and show decreased movement in liquid [57]. Additionally, the synaptic vesicle marker SNB-1::GFP accumulates in excitatory cholinergic axons of *fshr-1* mutants, consistent with decreased synaptic vesicle release [50]. However, the cell types where FSHR-1 acts to promote its neuromuscular effects and the mechanisms by which FSHR-1 signaling may impact neuromuscular transmission are unknown.

Here, we investigated the sites and mechanisms by which FSHR-1 controls neuromuscular signaling balance in *C*. *elegans*. Using behavioral assays and quantitative fluorescence imaging, we first confirmed the neuromuscular function defects in *fshr-1* loss-of-function mutants. We found the concomitant accumulation of synaptic vesicles observed in cholinergic synapses of *fshr-1-*deficient animals correlated with aberrant localization of active zone proteins in cholinergic motor neurons and with decreased synaptic vesicle release. Re-expression of *fshr-1* in the intestine, glia, or neurons of *fshr-1-*deficient worms restored muscle excitation to at or near wild type levels, and intestinal expression promoted restoration of synaptic vesicle localization. Conversely, intestine-specific knockdown of *fshr-1* reduced swimming. Finally, mutations in genes encoding effectors of FSHR signaling did not enhance the neuromuscular deficits of *fshr-1* loss-of-function mutants, consistent with actions in the FSHR-1 pathway to regulate neuromuscular function [27,51,56,58,59]. Similar epistasis experiments provide evidence that the α and β glycoprotein hormone orthologs, GPA2/GPLA-1/FLR-2 and GPB5/GPLB-1, which activate FSHR-1 *in vitro*, also act in a common pathway with FSHR-1 to regulate neuromuscular activity *in vivo*. Overall, our data provide evidence that the glycoprotein hormone receptor ortholog FSHR-1 is necessary and sufficient in the intestine (and perhaps acts in additional tissues) to regulate neuromuscular activity through effects on synaptic vesicle release. These findings expand our knowledge of the emerging neuroendocrine functions of this conserved receptor that has key roles in mammalian nervous system physiology and disease in humans.

## Results

### FSHR-1 is required for neuromuscular behaviors

**Fig 1:**
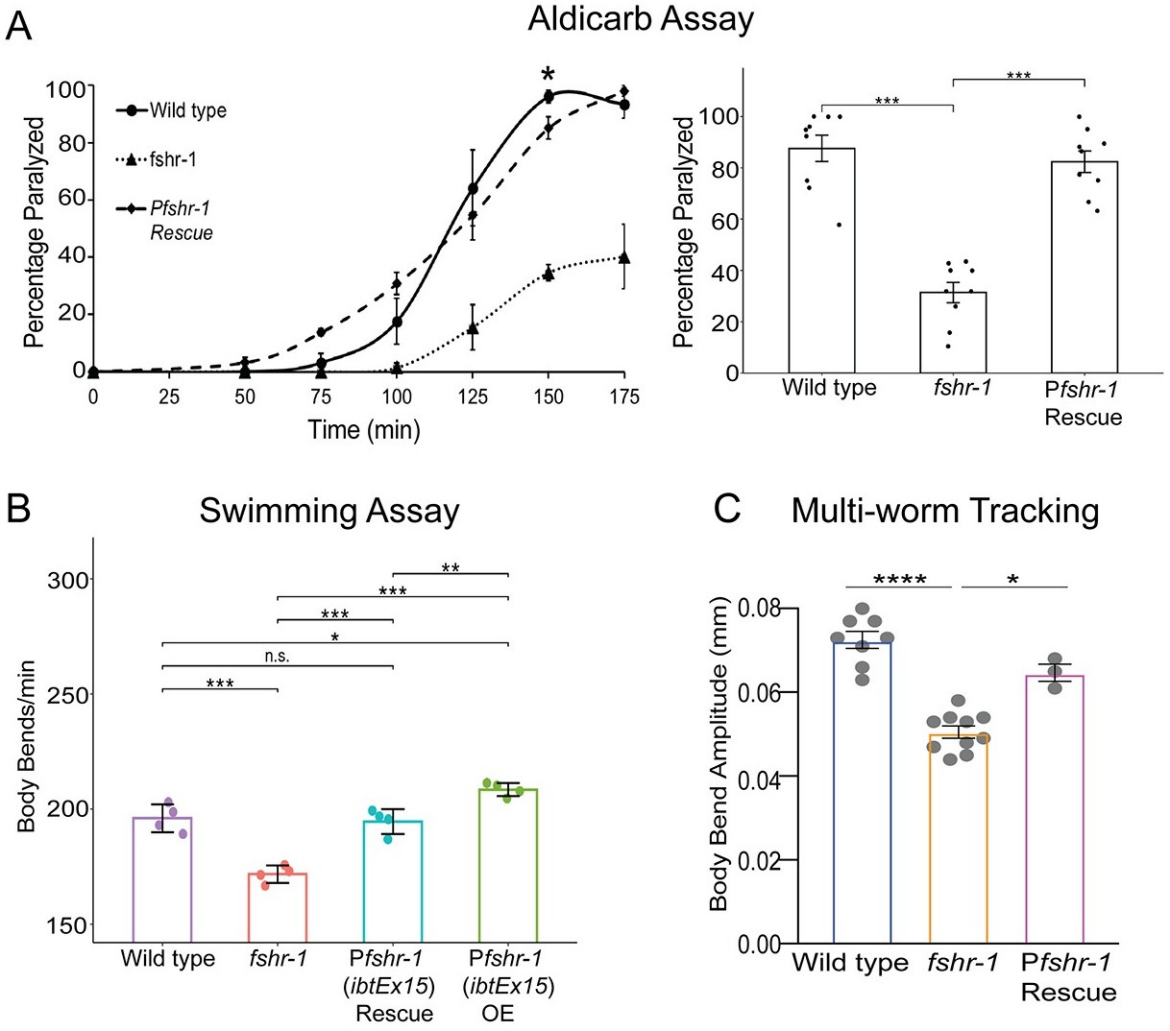
*fshr-1* is required for neuromuscular function in multiple assays. (A) Aldicarb paralysis assays, (B) swimming assays, and (C) multi-worm tracking assays were performed on wild type worms, *fshr-1(ok778)* mutants, rescued animals (Rescue) re-expressing *fshr-1* under the endogenous *fshr-1* promoter (P*fshr-1*, *ibtEx15*) in the *fshr-1* mutant background, and over-expression (OE) animals expressing *fshr-1* under the endogenous *fshr-1* promoter (P*fshr-1*, *ibtEx15*). (A) (*Left panel*) Representative aldicarb assays showing the mean percentage of worms paralyzed on 1mM aldicarb ± s.e.m. for n = 3 plates of approximately 20 young adult animals each per strain. (*Right panels*) Bar graphs showing cumulative mean data ± s.e.m. pooled from 3–4 independent experiments for worms paralyzed at the timepoint indicated by an asterisk (*) in the left panel. Scatter points show individual plate averages. Statistical significance of the data was analyzed using a one-way ANOVA and Tukey’s post hoc test or a Wilcoxon Rank Sum test followed by a Steel-Dwass multiple comparison analysis, as appropriate. (B) Mean body bends per minute ± S.D. obtained in swimming experiments. Each scatter point represents an independent experiment testing 30 animals per genotype. (C) Mean body bend amplitude ± S.D. obtained from multi-worm tracking experiments. Each scatter point represents an independent experiment from a population average of 20 animals. Statistical significance of the data was analyzed using a one-way ANOVA with Tukey’s multiple comparison. For A-C, results of analyses for which *p* ≤ 0.05 are indicated by horizontal lines above the bars. *p ≤ 0.05, **p ≤ 0.01, ***p ≤ 0.001, **** *p* ≤ 0.0001, n.s., not significant.

To explore the mechanisms by which *fshr-1* impacts neuromuscular synapse structure and function, we initially sought to define the cells in which *fshr-1* acts to control muscle excitation. First, we tested *fshr-1(ok778)* loss-of-function (*lf*) mutants for their sensitivity to the acetylcholine esterase inhibitor, aldicarb. Both excitatory cholinergic and inhibitory GABAergic inputs regulate the extent of muscle contraction at *C*. *elegans* body wall NMJs [60]. Aldicarb exposure leads to an accumulation of acetylcholine in the synaptic cleft, causing muscle hypercontraction and paralysis. Animals with mutations that increase cholinergic or decrease GABAergic signaling, or both, cause increased paralysis (aldicarb hypersensitivity) relative to wild type worms, whereas decreased paralysis (aldicarb resistance) is seen in animals with mutations that cause reduced cholinergic and/or increased GABAergic signaling [61]. We found that *fshr-1*(*lf*) mutants exhibited strong aldicarb resistance relative to wild type worms (Fig 1A), confirming previous results [50]. After 100 minutes on aldicarb, roughly 20% of *fshr-1* mutant worms were paralyzed, compared with approximately 80% of wild type (Fig 1A, *right panel*). The aldicarb resistance was rescued by expression of the *fshr-1* genomic sequence and native promoter region (Figs 1A and S1A) suggestive of a specific role for *fshr-1*.

Defects in neuromuscular transmission may be accompanied by locomotory deficits. To explore this possibility, we quantified the movement of *fshr-1* mutants in a liquid swimming assay [62]. We found reduced body bends in *fshr-1* mutants (~165 body bends/minute) compared to wild type (~195 body bends/minute), in line with our previously published results (Fig 1B) [57]. In addition, we found that *fshr-1* mutant animals have reduced body bending amplitude during crawling on agar (Fig 1C). The altered swimming and crawling behaviors were each rescued to wild type levels by expression of the *fshr-1* genomic sequence and endogenous promoter [27,48], demonstrating the specificity of the phenotype (Figs 1B and 1C and S1A). Overexpression of *fshr-1* under its endogenous promoter in wild type animals also modestly increased body bending rates, demonstrating the ability of *fshr-1* to drive increased neuromuscular function (Fig 1B). Additional high-resolution single-worm tracking experiments revealed reductions in head bending, locomotion speed, and foraging speed compared to wild type worms (S2 Fig), further supporting involvement of *fshr-1* in neuromuscular regulation and motility.

### FSHR-1 regulates cholinergic presynaptic structure and function

**Fig 2:**
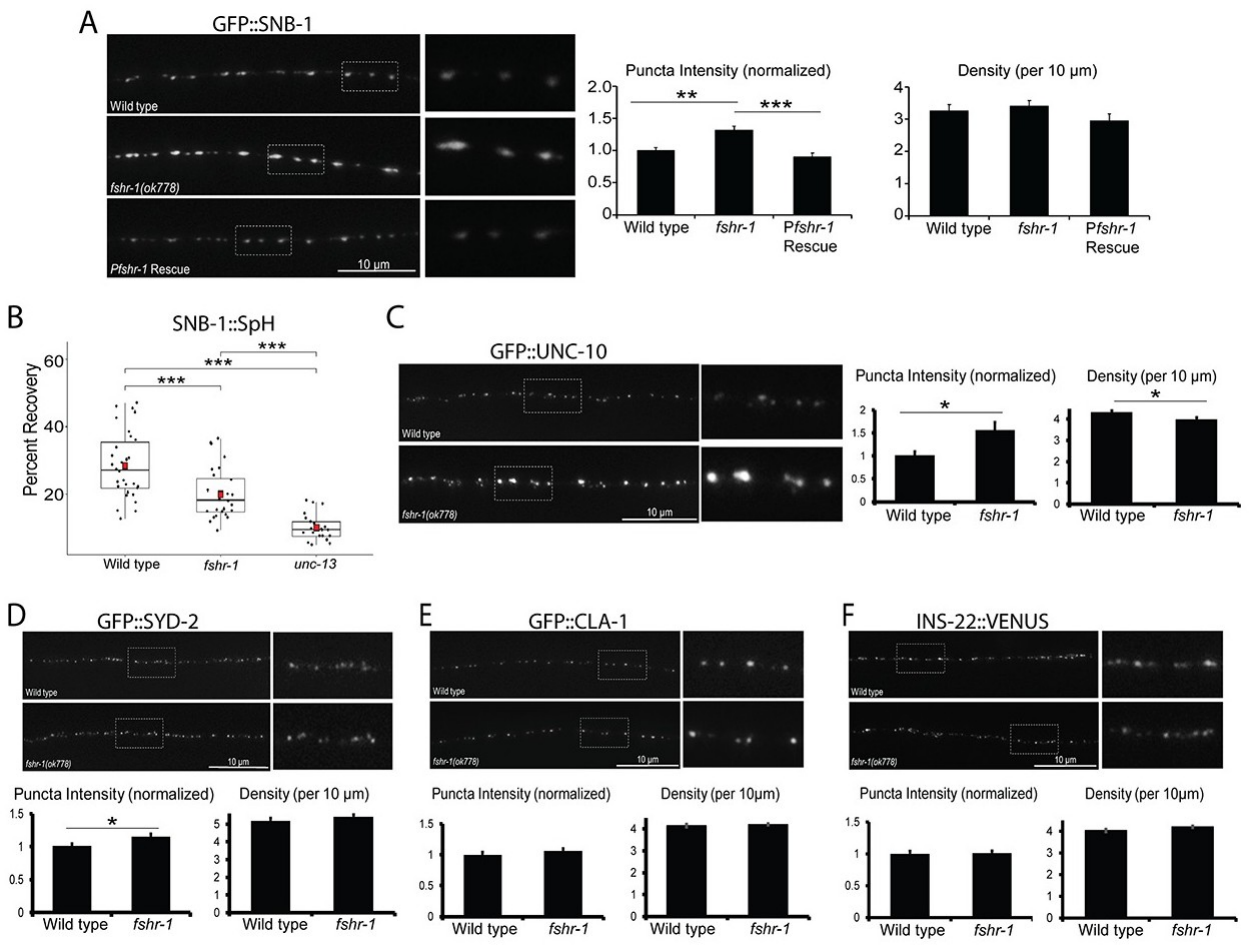
*fshr-1* mutants have decreased synaptic vesicle release accompanied by accumulation of some synaptic vesicle and active zone proteins. (A) Wild type worms, *fshr-1*(*ok778*) mutants, and *fshr-1* mutants re-expressing *fshr-1* genomic DNA under the endogenous *fshr-1* promoter (*Pfshr-1*, *agEx43*) that also expressed GFP::SNB-1 in cholinergic (ACh) neurons were imaged using a 100x objective. (*Left panel*) Representative images of the dorsal nerve cords halfway between the vulva and the tail of young adult animals. Boxed areas are shown in higher resolution to the right of the main images. (*Right panels*) Quantification of puncta (synaptic) intensity and puncta density (per 10 *μ*m) ± s.e.m. Puncta intensity is shown normalized to wild type. For (A), n = 24 animals imaged for wild type, n = 31 for *fshr-1*, and n = 26 for *Pfshr-1* rescue. (B) Percent recovery of SNB-1::Superecliptic pHluorin (SpH) fluorescence at ACh motor neuron presynapses following photobleaching in wild type, *fshr-1*(*ok778*), and fusion-defective *unc-13*(*se69*) animals. For wild type, n = 30 animals; for *fshr-1*, n = 28; for *unc-13*, n = 21. (C-F) Wild type or *fshr-1*(*ok778*) mutant animals that also expressed (C) GFP::UNC-10, (D) GFP::SYD-2, (E) GFP::CLA-1, or (F) INS-22::VENUS in ACh neurons were imaged using a 100x objective. (*Upper panels*) Representative images of the dorsal nerve cords halfway between the vulva and the tail of young adult animals. (*Lower panels*) Quantification of normalized puncta (synaptic) intensity and puncta density (per 10 *μ*m) ± s.e.m. For (C), n = 25 animals imaged for wild type, n = 24 for *fshr-1*. For (D), n = 31 for wild type, n = 35 for *fshr-1*. For (E), n = 30 for wild type, n = 31 for *fshr-1*. For (F), n = 31 for wild type, n = 35 for *fshr-1*. One-way ANOVA followed by Tukey’s post hoc tests were used to compare the means of the datasets in A and B; Student’s *t* tests were used to compare datasets in C-F. *p ≤ 0.05, **p ≤ 0.01, ***p ≤ 0.001. Upper two images and numeric data in (A) originally published in modified format in Hulsey-Vincent et al., 2023a [63].

Prior work showed that the aldicarb resistance of *fshr-1* mutants is paralleled by an accumulation of GFP::SNB-1/synaptobrevin-labeled synaptic vesicles in cholinergic axons of the dorsal nerve cord, suggestive of decreased acetylcholine release [50]. We confirmed that GFP::SNB-1 is increased in abundance at cholinergic presynaptic terminals. GFP::SNB-1 puncta intensity at cholinergic presynaptic terminals of *fshr-1* mutants is increased by approximately 40% compared to controls, while synapse density is not changed appreciably (Fig 2A) [63]. Expression of *fshr-1* under its own promoter was sufficient to reverse the increased GFP:SNB-1 fluorescence in cholinergic axons of *fshr-1* mutants (Fig 2A). In contrast, the GFP::SNB-1 puncta intensity and density in the GABAergic axons of *fshr-1* mutants were more variable, increasing moderately in a few experiments (S3A Fig). Thus, our data suggest *fshr-1* expression primarily regulates the levels of synaptic vesicles at cholinergic terminals of motor axons, though may also have less prominent roles at GABAergic synapses.

To determine if the accumulation of synaptic vesicles in cholinergic axons of *fshr-1(lf*) mutants may arise due to decreased cholinergic synaptic vesicle release, we performed fluorescence recovery after photobleaching (FRAP) experiments using cholinergic expression of synaptopHluorin (SpH), a pH-sensitive GFP variant fused to the luminal domain of SNB-1 (SNB-1::Superecliptic pHluorin) [64,65]. SpH fluorescence is largely quenched when exposed to the acidic environment of the vesicle lumen. Accordingly, SpH fluorescence primarily indicates vesicular material exposed at the surface of the plasma membrane following synaptic vesicle fusion, estimated to be ~30% of the total SpH pool [64]. The amount of fluorescence recovery after photobleaching provides a measure of new SpH on the surface as a result of synaptic vesicle release following photobleaching. Consistent with this, we found that SpH fluorescence recovered to about 30% in wild type worms within 50 s following photobleaching (Figs 2B and S4). In contrast, SpH fluorescence recovery (measured after photobleaching) was significantly reduced in *fshr-1(lf)* mutants (~35% decrease) (Fig 2B). For comparison, fluorescence recovery was decreased by ~70% in *unc-13*(*s69*) mutants that have severe defects in vesicle fusion (Fig 2B) [66–68]. Together, these data demonstrate that FSHR-1 signaling promotes the localization and/or release of cholinergic synaptic vesicles.

We next asked whether other aspects of synapse structural organization might be altered by FSHR-1 signaling. Specifically, we tested whether the localizations of several active zone proteins known to be involved in synaptic vesicle docking and release, UNC-10/RIM, SYD-2/ Liprinα, and CLA-1/Clarinet, are altered in *fshr-1* mutants [69–72]. We found a ~55% increase in GFP::UNC-10 fluorescence intensity at cholinergic synapses and a small, but statistically significant (~8%) decrease in the density of GFP::UNC-10 puncta (Fig 2C). Neither of these parameters differed for mCherry::UNC-10 in the GABAergic motor neurons of *fshr-1* mutants (S3B Fig). In contrast, we observed a modest elevation in the synaptic levels of GFP::SYD-2/Liprinα at both cholinergic (~14%) and GABAergic (~20%) presynapses of *fshr-1-*deficient animals while GFP::SYD-2 puncta density was not significantly changed (Figs 2D and S3C). Finally, neither the intensity nor density of cholinergic GFP::CLA-1/Clarinet synaptic puncta were significantly altered in *fshr-1* mutants (Fig 2E). Together, these results argue that *fshr-1* negatively regulates the delivery or turnover of UNC-10/RIM at cholinergic synaptic terminals, suggesting a potential mechanism through which FSHR-1 may affect the abundance of cholinergic synaptic vesicles at releases sites and neurotransmission.

Neuropeptide-containing dense core vesicles (DCVs) are also released from motor neuron synapses, and neuropeptide signaling can influence neuromuscular transmission [73–78]. To determine whether loss of FSHR-1 also impacts DCVs, we assessed whether *fshr-1* mutants had altered accumulation of the neuropeptide and DCV marker, INS-22::Venus, in cholinergic motor neurons of the dorsal nerve cord [50, 77]. Unlike the effect of *fshr-1* loss-of-function on synaptic vesicles and several active zone proteins, there was no change in the localization or abundance of INS-22::VENUS at cholinergic presynapses (Fig 2F). This result suggests that FSHR-1 signaling specifically regulates the release of synaptic vesicles, but not dense core vesicles, from cholinergic motor neurons.

Next, we sought to determine the impact of FSHR-1 signaling on muscle AChR activity. We performed paralysis assays using the acetylcholine receptor agonist, levamisole [79]. Levamisole sensitivity depends upon the number of levamisole-sensitive acetylcholine receptors, with more receptors causing increased sensitivity to levamisole-induced paralysis, as well as on the internal excitation and metabolic state of the muscle cells [60,80,81]. Surprisingly, we found that *fshr-1(lf)* animals exhibited levamisole hypersensitivity—nearly 100% paralysis at 100 min on 200 μm levamisole compared to only ~50% paralysis of wild type worms. This sensitivity was fully restored by expression of *fshr-1* under its own promoter (S5 Fig). These data most likely point toward a compensatory increase in postsynaptic muscle acetylcholine receptors or excitation machinery, as has been observed previously for other mutants with decreased ACh release [82,83]. This compensation is insufficient to overcome the reduction in acetylcholine release, however, as *fshr-1*(*lf*) mutants remain deficient in neuromuscular behaviors (swimming, aldicarb-induced paralysis, and crawling phenotypes) despite increased muscle excitability.

### FSHR-1 acts in the intestine and other distal tissues to regulate neuromuscular function

**Fig 3:**
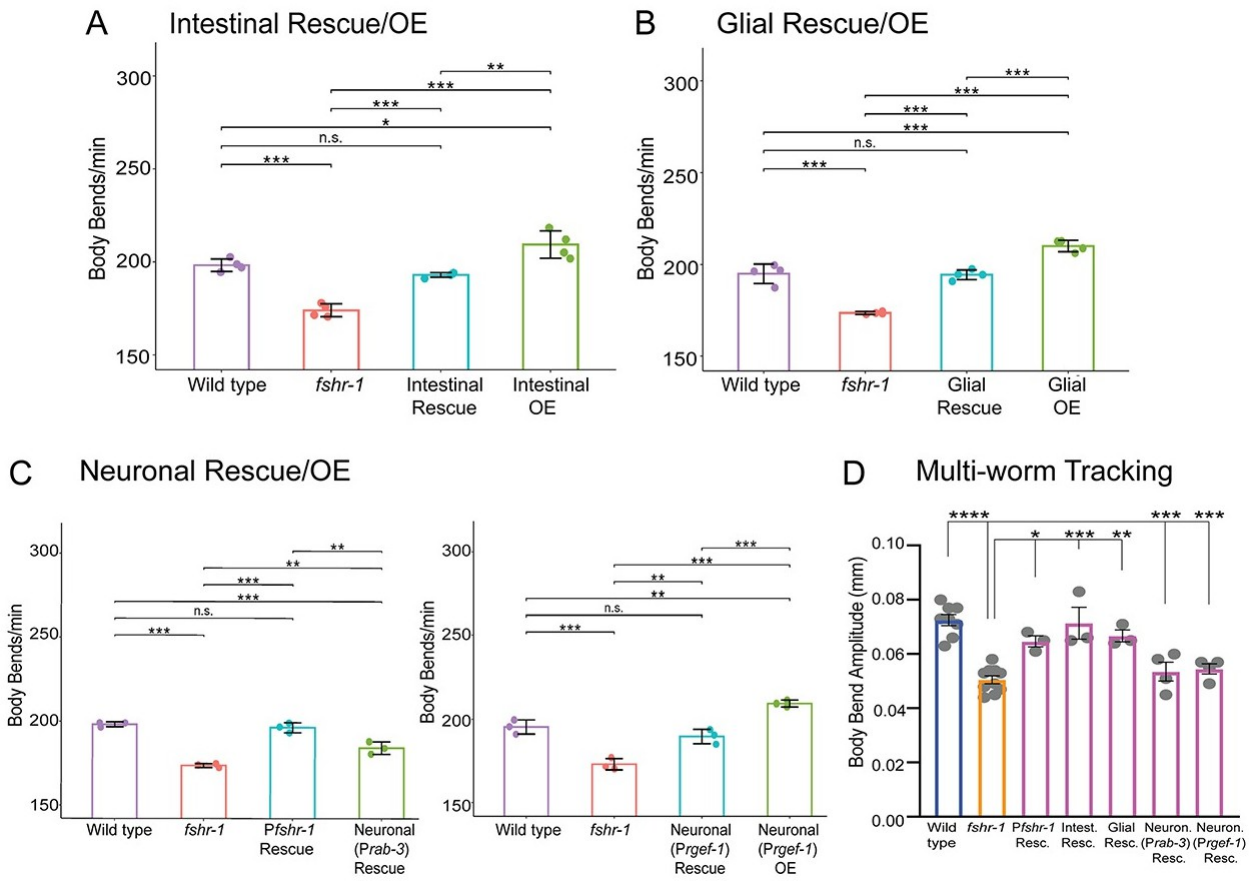
*fshr-1* re-expression in multiple distal tissues is sufficient to restore neuromuscular signaling to *fshr-1(lf)* mutants. (A-C) Swimming assays and (D) multi-worm tracking experiments were performed on wild type worms, *fshr-1(ok778)* mutants, and animals re-expressing *fshr-1* under either an intestinal promoter (A, D; *Pges-1*, *ibtEx35)*, a pan-glial promoter (B, D; P*mir-228*, *ibtEx51*) or a pan-neuronal promoter (C, D; *Prab-3*, *ibtEx34* or *Prgef-1*, *ibtEx67*) in either a wild type (OE, overexpression) or *fshr-1* mutant background (Rescue/Resc.). (A-C) Mean body bends per minute ± S.D. obtained in swimming experiments. Each scatter point represents an independent experiment testing 30 animals per genotype. (D) Mean body bend amplitude ± S.D. obtained from multi-worm tracking experiments. Each scatter point represents an independent experiment from a population average of 20 animals. Statistical significance of the data was analyzed using a one-way ANOVA with Tukey’s multiple comparison. Colors on the bars indicate strain groups as follows: Blue = wild type, yellow = *fshr-1* mutants; magenta = *fshr-1* rescue strains. For all experiments, one-way ANOVA and Tukey’s post hoc tests were used to compare the means of the datasets (*p ≤ 0.05, ** p ≤ 0.01, ***p 0.001; **** *p* ≤ 0.0001, n.s., not significant; in (D), asterisks above the two Neuron Rescue strains indicate statistically significant differences from wild type worms, whereas asterisks above the P*fshr-1*, Intestinal Rescue, and Glial Rescue bars indicate differences from *fshr-1* mutants).

Previous studies reported prominent *fshr-1* expression in the intestine, pharynx, vulva, and spermatheca, as well as in undescribed neurons and glia in the head [27,48,49]. Intestinal, glial, and neuronal *fshr-1* expression have all been implicated in controlling different aspects of *fshr-1* function, including pathogen susceptibility, stress responses, phenoptosis, body size, and lipid homeostasis [48,51–56] To determine the tissues where *fshr-1* expression may be most important for the regulation of ACh release from motor neurons, we performed tissue-specific rescue and overexpression of *fshr-1* in *fshr-1* mutants and wild type animals, respectively. We found that restoration of *fshr-1* expression using intestinal, pan-glial, or pan-neuronal promoters in *fshr-1* mutants was sufficient to restore partially or fully wild type swimming rates (Figs 3A–3C and S1B and S1C) and crawling body bend amplitude (Fig 3D), as well as levamisole resistance (S5A–S5C Fig). Intestinal or pan-glial expression provided more robust rescue in comparison to neuronal rescue (*P*rab-3 or *Prgef-1* promoters) (Figs 3, S1B and S1C and S5A–S5C). Additionally, we observed that overexpression of the same *fshr-1* transgenes in wild type animals modestly, but significantly, increased swimming rates compared to control (Fig 3). Taken together, these results indicate that *fshr-1* activation is sufficient in multiple distal tissues to regulate neuromuscular signaling.

**Fig 4:**
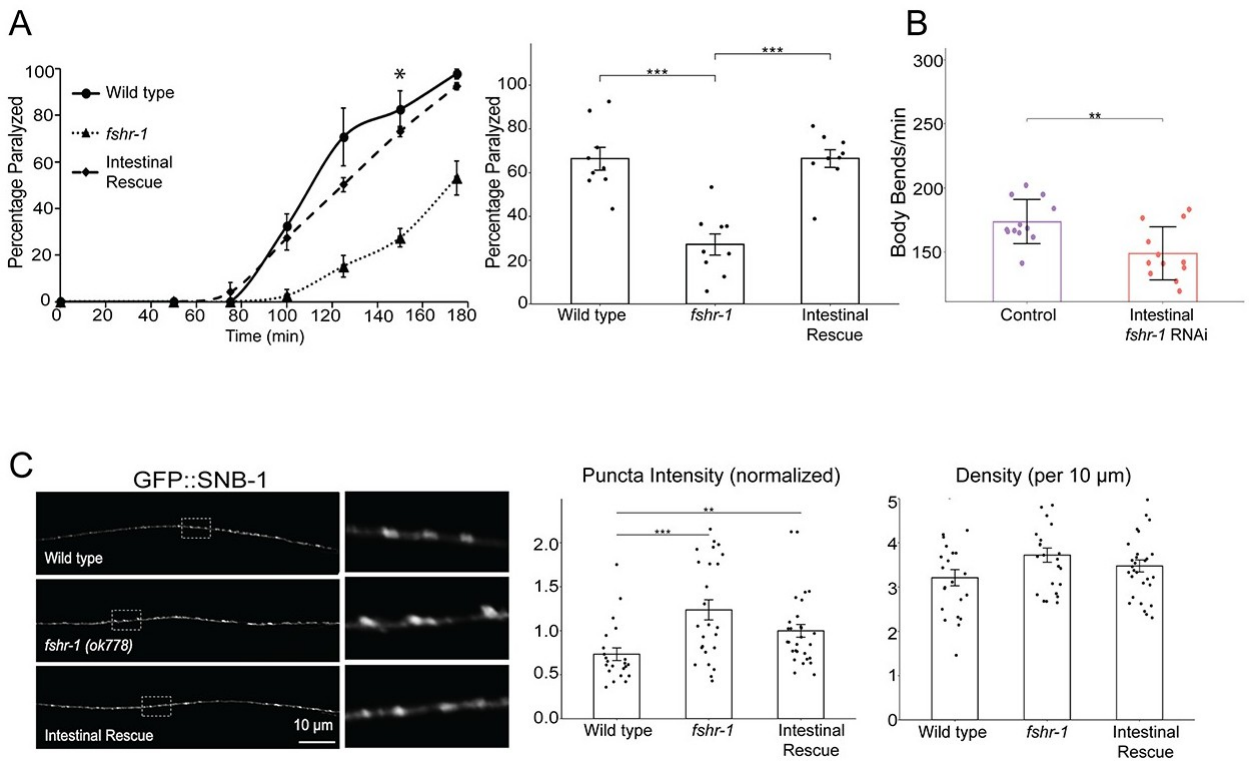
*fshr-1* expression in the intestine is necessary and sufficient for neuromuscular function and structure. (A, C) Intestinal rescue (*Pges-1*, *ibtEx35*) and (B) Intestine-specific RNAi [*Pnhx-2*::*rde-1; rde-1(ne219)*] effects on *fshr-1* neuromuscular phenotypes. (A) (*Left panel*) Representative aldicarb assays showing the mean percentage of wild type, *fshr-1*(*ok778*) mutant, and intestinal rescue worms paralyzed on 1mM aldicarb ± s.e.m. for n = 3 plates of approximately 20 young adult animals per strain. (*Right panel*) Bar graphs showing cumulative data ± s.e.m.pooled from 3 independent aldicarb experiments for worms paralyzed at the timepoint indicated by an asterisk (*) in the left panel. Scatter points show individual plate averages. (B) Mean body bends per minute ± S.D. obtained in swimming experiments done on L4440 vector only-treated (Control) or *fshr-1* RNAi-treated worms (Intestinal *fshr-1* RNAi). (C) Wild type, *fshr-1*(*ok778*) mutant, and intestinal rescue worms that also expressed GFP::SNB-1 in ACh neurons were imaged using a 100x objective. (*Left panels*) Representative images of the dorsal nerve cords halfway between the vulva and the tail of young adult animals. Boxed areas are shown in higher resolution to the right of the main images. (*Right panels*) Quantification of normalized puncta (synaptic) intensity and puncta density (per 10 *μ*m) ± s.e.m. Scatter points show individual worm means (n = 22–29 animals per genotype). One-way ANOVA and Tukey’s post hoc were used to compare the means of the datasets; *p ≤ 0.05, **p ≤ 0.01, ***p ≤ 0.001, **** *p* ≤ 0.0001, n.s., not significant.

The functions of FSHR-1 characterized to date have been most closely linked to its intestinal expression [48,52,53,55,56]. Hence, we focused our analysis on the role of intestinal *fshr-1* in controlling neuromuscular function. In addition to restoration of swimming and crawling behavior, we found that re-expression of *fshr-1* in the intestine alone was sufficient to restore wild type aldicarb paralysis rates (Figs 4A and S1B). To test the specificity of the intestinal *fshr-1* requirement, we performed swimming experiments following tissue-specific depletion of *fshr-1* from the intestine via feeding RNA interference (RNAi) in *kbIs7*;*rde-1(ne219)* worms, which restrict RNAi efficacy to the intestinal cells [84]. *fshr-1* knockdown animals exhibited a ~15% decline in body bending rates compared to worms treated with bacteria containing an empty vector control (Fig 4B). These results match those seen for *fshr-1* genetic *lf* animals and demonstrate that *fshr-1* is necessary, as well as sufficient, in the intestine for neuromuscular function. Importantly, these functional effects of intestinal *fshr-1* correlated with structural effects at cholinergic synapses, as intestinal re-expression of *fshr-1* partially restored the aberrant synaptic vesicle accumulation seen in *fshr-1* mutants (Fig 4C). Rescue of GFP::SNB-1 accumulation was not observed in *fshr-1* mutants with glial-specific or pan-neuronal-specific restoration of *fshr-1* expression (S6 Fig), despite some glial and neuronal rescue of behavioral phenotypes (Figs 3 and S1C). Additionally, although expression of *fshr-1* in either cholinergic or GABAergic motor neurons rescued aldicarb and/or swimming deficits in *fshr-1* mutants, GFP::SNB-1 accumulation was exacerbated in these strains, suggesting mis-expressed FSHR-1 in neurons is sufficient to impact neuromuscular function through unknown mechanisms (S7 Fig). Similarly, muscle-specific *fshr-1* mis-expression increased swimming rates of *fshr-1* mutants compared to wild type animals, while failing to rescue levamisole sensitivity (S5D and S5E Fig). Overall, our tissue-specific expression data suggest that *fshr-1* expression in the intestine is necessary and sufficient for the organization of cholinergic presynaptic terminals and neuromuscular transmission; however, FSHR-1 may also act in other distal tissues, including glia and possibly head neurons, to regulate muscle excitation.

### FSHR-1 acts upstream of canonical G protein and lipid kinase pathways to regulate neuromuscular function

**Fig 5:**
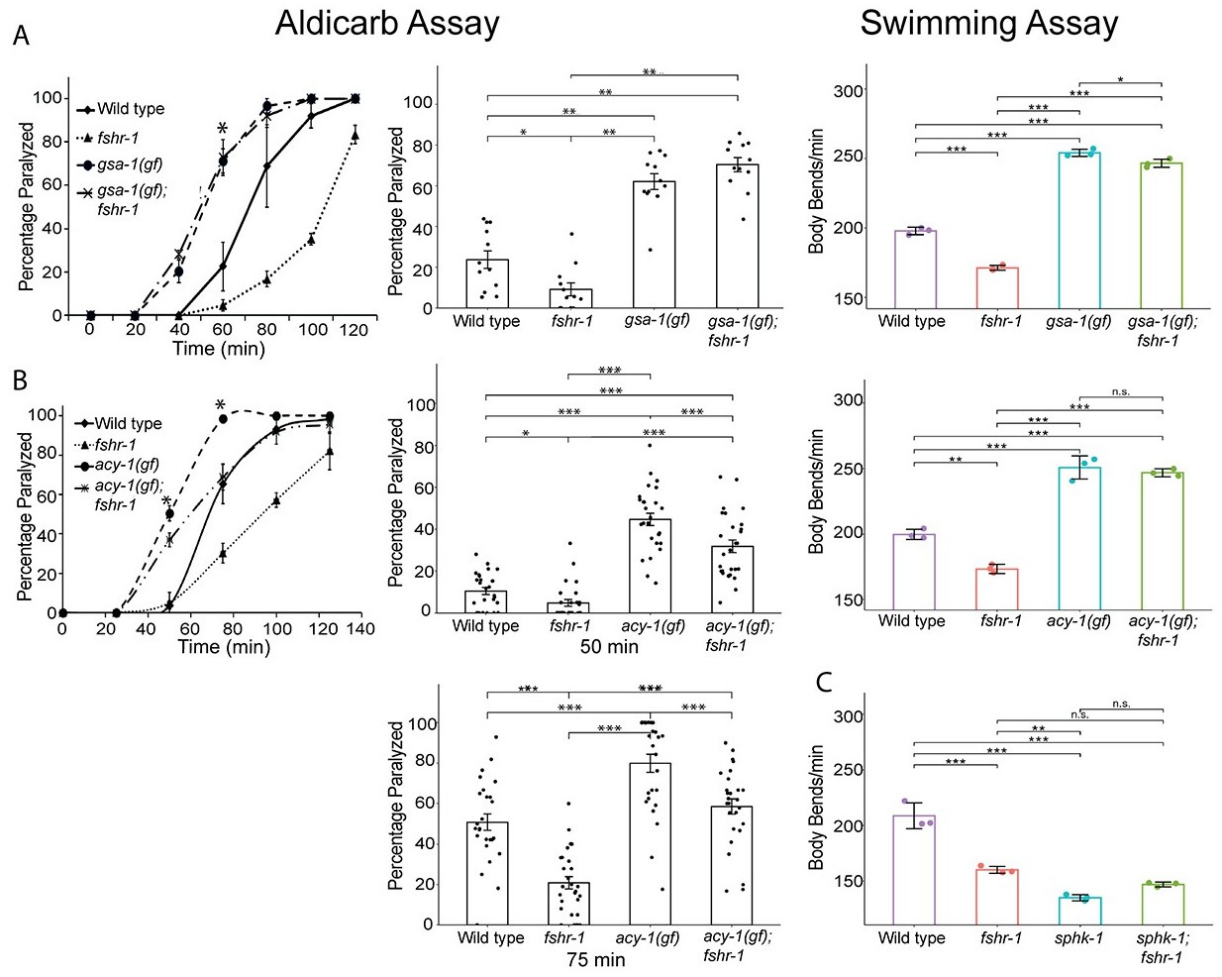
*gsa-1(gf)*, *acy-1(gf)*, and *sphk-1(lf)* mutations suppress *fshr-1(lf)* aldicarb phenotypes consistent with a downstream function. Aldicarb paralysis and swimming assays were performed on wild type worms, *fshr-1(ok778)* mutants, and (A) *gsa-1(ce81*) or (B) *acy-1*(*md1756*) gain-of-function (*gf*) mutants, or (C) *sphk-1*(*ok1097*) loss-of-function mutants, along with their respective double mutants (*gsa-1;fshr-1*, *acy-1;fshr-1*, or *sphk-1;fshr-1*). (*Left panels*) Representative aldicarb assays showing the mean percentage of worms paralyzed on 1mM aldicarb ± s.e.m. for n = 3 plates of approximately 20 young adult animals each per strain. (*Center panels*) Bar graphs show cumulative data ± s.e.m. pooled from (A) 4 or (B) 8–9 independent aldicarb experiments for worms paralyzed at the timepoint indicated by an asterisk (*) in the upper panels. Scatter points show individual plate averages. Statistical significance of the data was analyzed using a Wilcoxon Rank Sum test followed by a Steel-Dwass multiple comparison analysis, as appropriate. Results of analyses for which *p* ≤ 0.05 are indicated by horizontal lines above the bars. (*Right panels*) Mean body bends per minute ± S.D. obtained in swimming experiments. Each scatter point represents an independent experiment testing 30 animals per genotype, analyzed by one-way ANOVA and Tukey’s post hoc test, *p ≤ 0.05, **p ≤ 0.01, ***p ≤ 0.0001, n.s., not significant.

We next sought to define the signaling pathway components involved in FSHR-1 control of neuromuscular function. Mammalian glycoprotein hormone receptors can act through several different signaling pathways depending on the cell type and context [85]. Previous studies in *C*. *elegans* demonstrated that FSHR-1 acts upstream of genes encoding the Gα_S_ protein GSA-1 and the adenylyl cyclase ACY-1 [27,56,76], and FSHR-1 can activate cAMP signaling when expressed in cultured mammalian cells [48,86]. To assess the potential involvement of these downstream players in FSHR-1 modulation of cholinergic neuromuscular signaling, we used strains carrying gain-of-function (*gf*) GSA-1 and ACY-1 alleles that have been previously shown to promote muscle excitation [87,88]. The aldicarb resistance of *fshr-1* mutants was fully suppressed in *gsa-1(gf);fshr-1(lf)* double mutants, which carry a *gsa-1(gf)* mutation that prevents GTP hydrolysis (Fig 5A). Increased neuromuscular function induced by *gsa-1(gf)* mutations also occurred in swimming experiments, where *gsa-1(gf)* also suppressed the reduced body bending rates of *fshr-1(lf)* mutants (Fig 5A). We noted similar suppression of the *fshr-1(lf)* swimming phenotypes by *acy-1(gf)* mutation; however, the aldicarb resistance of *fshr-1* mutants was only partially suppressed by *acy-1(gf)* activating mutations [88–90] (Fig 5B). Notably, the suppression was reproducibly strongest at early timepoints, then declined. Similarly reduced effects of the *acy-1(gf)* mutation compared to *gsa-1(gf)* were observed previously and may reflect a weaker *gf* allele [88] or changes in signaling over time. Together, these data suggest that the canonical Gα_S_ and adenylyl cyclase enzymes act downstream of *fshr-1* to regulate cholinergic transmission.

Along with G protein pathway components, both mammalian FSHR and *C*. *elegans* FSHR-1 have been implicated in signaling pathways containing the lipid kinase SPHK-1, which converts sphingosine to sphingosine-1-phosphate [51,59]. For example, neuronal FSHR-1 signaling controls SPHK-1 localization to intestinal mitochondria in response to intestinal oxidative stress [51]. Additionally, FSHR signaling promotes SPHK activation, leading to proliferation of epithelial ovarian cancer cells [59]. SPHK-1, which is expressed in both neurons and the intestine, has also been implicated in the regulation of neuromuscular structure and function via its ability to promote recruitment of UNC-13/Munc13 to presynaptic terminals following muscarinic ACh receptor activation [91,92]. Consistent with this, *sphk-1* mutants are known to have reduced rates of swimming and aldicarb-induced paralysis, similar to *fshr-1* mutants [50,91,92]. We asked whether *fshr-1* acts upstream of *sphk-1* by comparing the phenotypes of *fshr-1*(*ok778*) and *sphk-1(ok1097)* single and double loss-of-function mutants in swimming experiments. We found that *fshr-1;sphk-1* double mutants had body bending rates that were non-additive, suggesting these genes do act together to regulate neuromuscular function (Fig 5C).

### FSHR-1 ligands, the glycoprotein hormone subunit orthologs GPLA-1 and GPLB-1, act in a common pathway with FSHR-1 to regulate neuromuscular function

**Fig 6:**
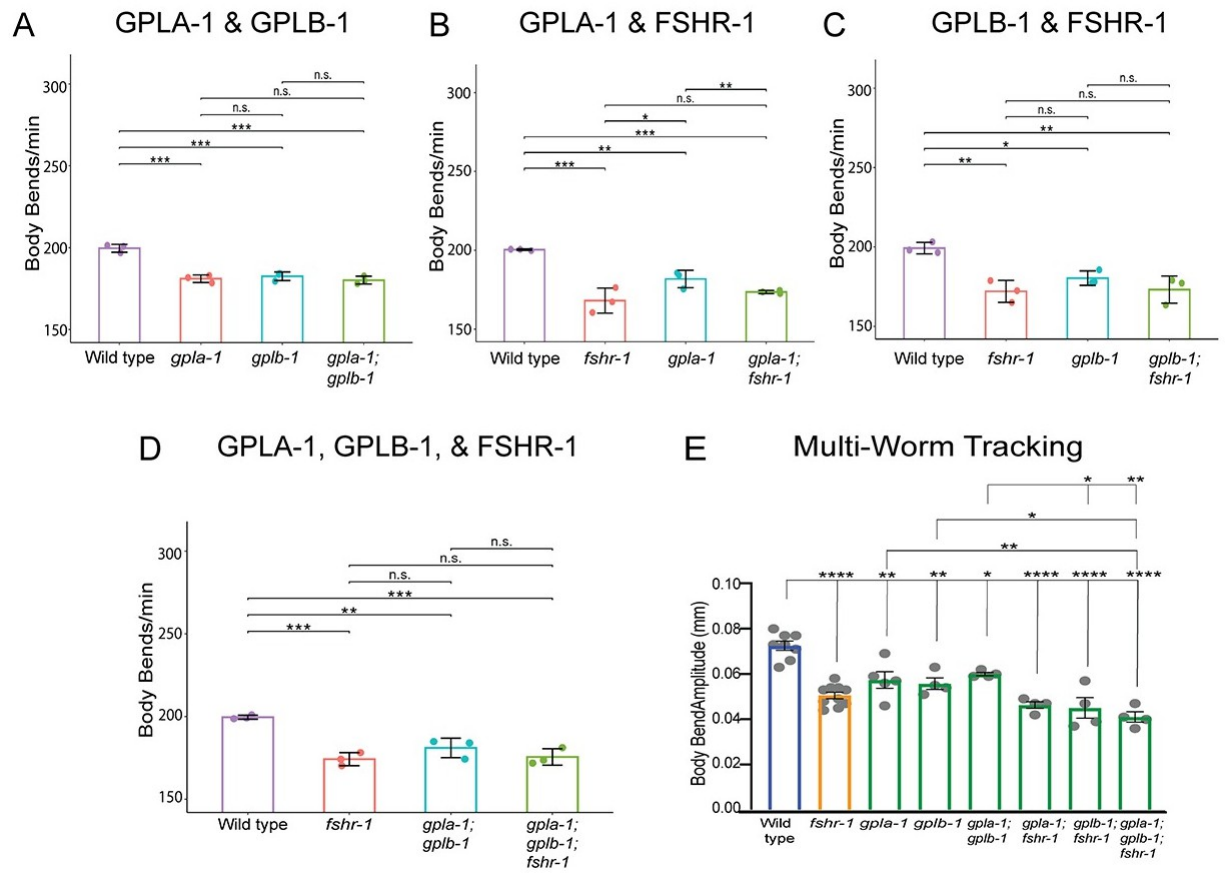
GLPA-1/FLR-2/GPA2 and GPLB/GB5 glycoprotein act in a common genetic pathway with FSHR-1 at the NMJ. (A-D) Mean body bends per minute ± S.D. obtained in swimming experiments testing *fshr-1* and α and β glycoprotein ligand mutants [*fshr-1(ok778)*, *gpla-1(ibt1) α*, *gplb-1* (*ibt4*) β worms] or combinations of double and triple mutants in these genes. Each scatter point represents an independent experiment testing 30 animals per genotype. (E) Mean body bend amplitude obtained from multi-worm tracking experiments. Each scatter point represents an independent experiment with a population average of 20 animals. Colors indicate groups of mutants as follows: blue = wild type, yellow = *fshr-1*; green = glycoprotein mutants. For all experiments, one-way ANOVA and Tukey’s post hoc tests were used to compare the means of the datasets (*p ≤ 0.05, **p ≤ 0.01, ***p ≤ 0.001; ****p ≤ 0.0001; n.s., not significant).

GPLA-1/GPA2 and GPLB-1/GPB5 encode *C*. *elegans* orthologs of the glycoprotein hormone subunits GPA2 and GPB5, which are ancestral to all glycoprotein hormones, including FSH in vertebrates [93–96]. GPLA-1/GPA2 and GPLB-1/GPB5 activate FSHR-1 *in vitro* and act as cognate FSHR-1 ligands in body size regulation [48]. GPLA-1/GPA2 was also implicated with FSHR-1 in regulating *C*. *elegans* lipid homeostasis and phenoptosis [55,56]. We tested whether *gpla-1* and *gplb-1* act with *fshr-1* to control neuromuscular function. We found that loss-of-function mutants in either gene reduced swimming and crawling rates by ~15% compared to wild type worms–levels similar to those observed for *fshr-1(lf)* mutants (Fig 6A–6C). The movement deficits of *gpla-1(lf)* and *gplb-1*(*lf*) single mutants were not enhanced when in combination with each other [*gpla-1(lf);gplb-1*(*lf*) double mutants] (Fig 6A) or with *fshr-1*(*lf*) [*gpla-1(lf); fshr-1*(*lf*) and *gplb-1*(*lf*);*fshr-1*(*lf*) double mutants or *gpla-1(lf);gplb-1(lf);fshr-1* triple mutants] (Fig 6B–6E), suggesting these glycoprotein subunit orthologs act together to regulate NMJ function, as well as with *fshr-1*. Together, these data suggest that both *gpla-1* and *gplb-1* are required to promote neuromuscular function and that they do so in a pathway that also requires FSHR-1.

### Intestinal FSHR-1 effects on the NMJ require the glycoprotein hormone ligands and intestinal downstream effectors

**Fig 7:**
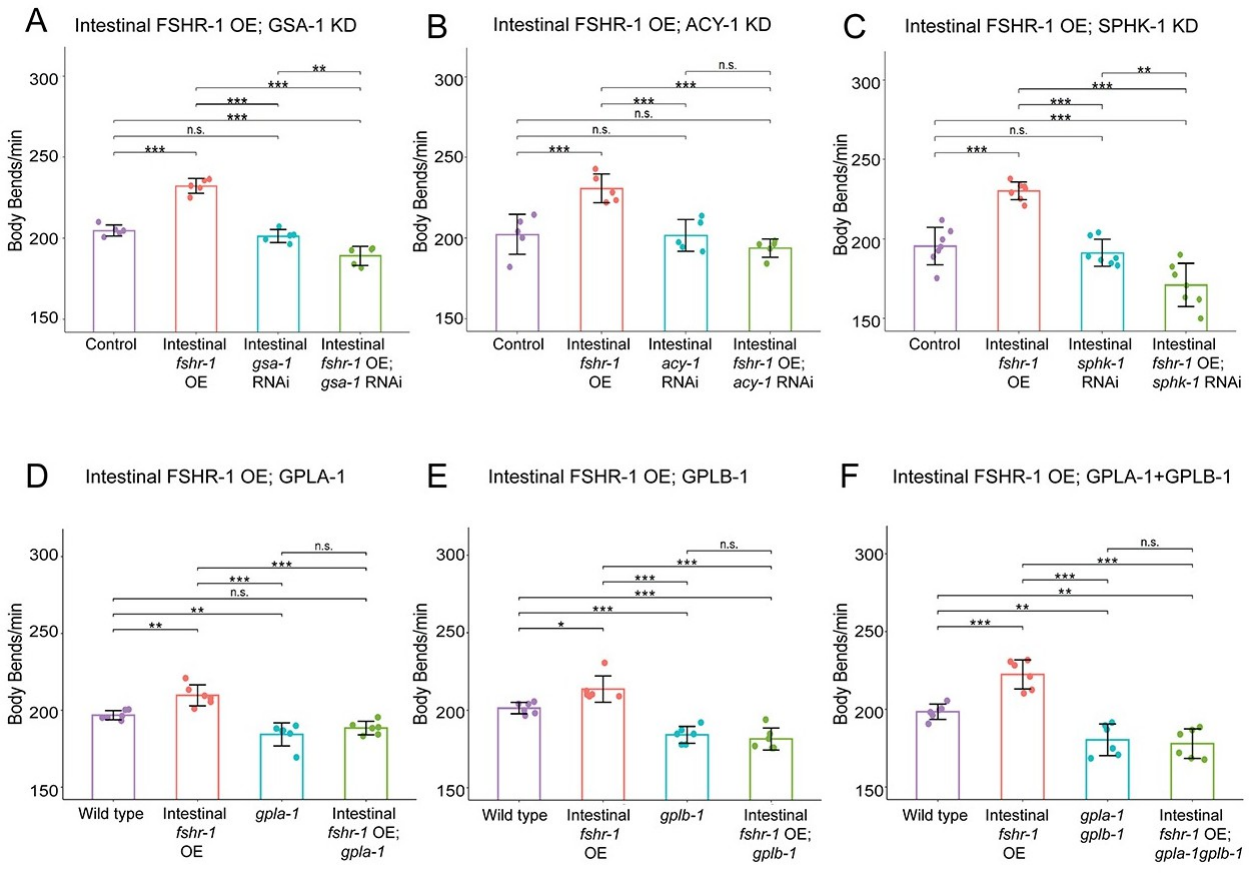
Regulation of neuromuscular function by intestinal FSHR-1 requires intestinally expressed GSA-1, ACY-1, AND SPHK-1, as well as the GLPA-1 and GPLB glycoproteins. (A-C) Mean body bends per minute ± S.D. obtained in swimming experiments testing intestinal RNAi sensitized [*Pnhx-2*::*rde-1; rde-1(ne219)*] control-treated animals overexpressing *fshr-1* in the intestine (*Pges-1*, *ibtEx35*, Intestinal *fshr-1* OE), treated with feeding RNAi targeting (A) *gsa-1*, (B) *acy-1*, or (C) *sphk-1*, or animals with intestinal FSHR-1 overexpression and *gsa-1*, *acy-1*, or *sphk-1* RNAi compared to L4440 empty vector-treated worms (Control). Each scatter point represents an independent experiment testing 10 animals per genotype. (D-F) Mean body bends per minute ± S.D. obtained in swimming experiments testing animals overexpressing *fshr-1* in the intestine (*Pges-1*, *ibtEx35*), (D) *gpla-1*(*ibt1*), (E) *gplb-1*(*itb4*), or (F) *gpla-1gplb-1* mutations compared to animals with both intestinal *fshr-1* overexpression and glycoprotein mutation, and wild type controls. Each scatter point represents an independent experiment testing 30 animals per genotype. For all experiments, one-way ANOVA and Tukey’s post hoc tests were used to compare the means of the datasets (*p ≤ 0.05, **p ≤ 0.01, ***p ≤ 0.001; n.s., not significant).

Our data demonstrate that *fshr-1* works in a common genetic pathway with the known intracellular effectors, *gsa-1*, *acy-1*, and *sphk-1*, as well as with the glycoprotein ligands *gpla-1* and *gplb-1*, to regulate neuromuscular function. Given their expression in multiple tissues we used intestine-specific feeding RNAi to test whether intestinal *gsa-1*, *acy-1*, or *sphk-1* is required for the effects of intestinal *fshr-1* overexpression on swimming rates. We found that knocking down expression of each of these downstream effectors, while causing minimal effects on swimming behavior when knocked down on their own, was sufficient to reduce the increased swimming rates caused by intestinal *fshr-1* overexpression (Fig 7A–7C). These results demonstrate that *gsa-1*, *acy-1*, and *sphk-1* are required for the ability of intestinal *fshr-1* to drive increased neuromuscular function, particularly under conditions of heightened FSHR-1 activation or expression.

Looking upstream, we also asked whether the activity of FSHR-1 in the intestine requires the GPLA-1 and GPLB-1 ligands. We found that animals overexpressing *fshr-1* in the intestine but carrying deletions in *gpla-1* and/or *gplb-1* were unable to promote the increased swimming rates seen with intestinal *fshr-1* overexpression alone (Fig 7D–7F). These data indicate that despite some evidence of constitutive receptor activity in other systems, the activity of the FSHR-1 receptor in the *C*. *elegans* intestine requires the presence of the GPLA-1 and GPLB-1 ligands.

## Discussion

**Fig 8:**
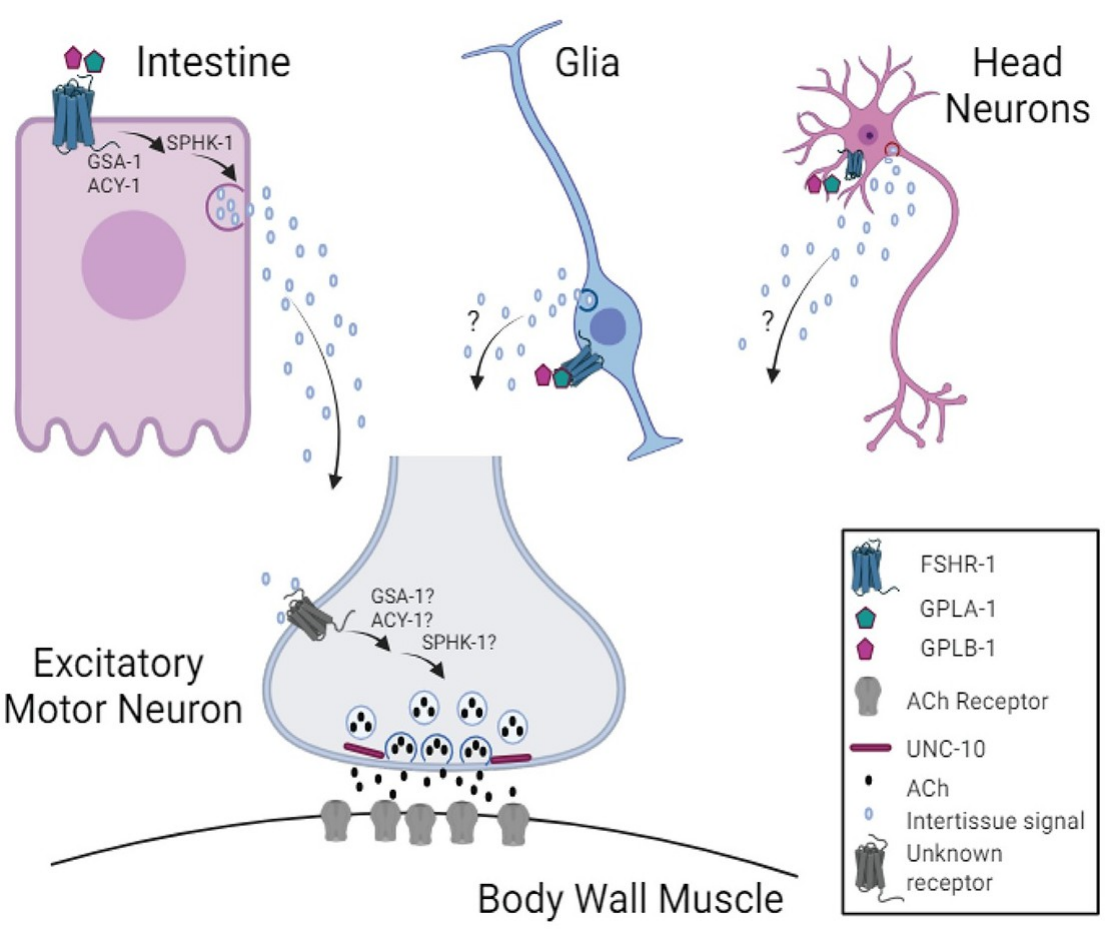
Hypothesized mechanism for FSHR-1 cross-tissue regulation of neuromuscular function. Our data support a model in which FSHR-1 acts in distal tissues, including the intestine, and possibly glia or head neurons, to promote neurotransmitter release from cholinergic body wall motor neurons leading to body wall muscle excitation. This cell non-autonomous regulation of neuromuscular function likely requires secretion of currently unknown molecules from the intestine or other distal tissues in response to FSHR-1 activation that, in turn, act on unknown receptors on the cholinergic motor neurons to promote synaptic vesicle release through effects on UNC-10/RIM. Results of our epistasis experiments further suggest that FSHR-1 is activated by the glycoprotein ligands, GPLA-1/GPA2 and GPLB-1/GPB5. Known effectors of FSHR-1 in other contexts, GSA-1, ACY-1, and SPHK-1, act downstream of FSHR-1 in the intestine during neuromuscular junction regulation; however, further studies will be required to determine whether these molecules also act in motor neurons themselves or other distal tissues following FSHR-1 activation. Created in BioRender. BioRender.com/u21o416.

FSHR-1 is a conserved GPCR implicated in diverse aspects of *C*. *elegans* physiology, including germline differentiation, stress responses, organism growth, and neuromuscular signaling. Here, we investigated the mechanisms by which FSHR-1 regulates synaptic transmission at the *C*. *elegans* neuromuscular junction. Our data demonstrate that *fshr-1* acts cell non-autonomously in the intestine, as well as potentially in other distal tissues, including glia and/or head neurons, to promote muscle contraction. Quantitative imaging of synaptic proteins shows that *fshr-1* regulates the localization and release of cholinergic synaptic vesicles, as well as the abundance of the synaptic vesicle docking and priming factor, UNC-10/RIM, in cholinergic motor neurons. Epistasis experiments support a model in which Gα_S_ and adenylyl cyclase, as well as SPHK signaling, act downstream of FSHR-1 in its control of neuromuscular activity, while the FSHR-1 ligands GPLA-1/GPA2 and GPLB-1/GPB5 act upstream in this context. Together, these results suggest a mechanism by which *fshr-1* promotes muscle excitation through effects manifested predominantly in cholinergic motor neurons but initiated through FSHR-1 signaling pathways in the intestine and/or other neurosecretory cells (Fig 8).

### FSHR-1 acts in the intestine to promote muscle excitation

Prior work used aldicarb and swimming assays to establish a requirement for FSHR-1 in promoting muscle excitation under both normal and oxidative stress conditions [50,57] and demonstrated animals lacking *fshr-1* accumulate the synaptic vesicle-associated protein GFP::SNB-1 in cholinergic motor neurons [50]. Our cell type-specific rescue and knockdown experiments support a model in which FSHR-1 activity in the intestine is both necessary and sufficient for cell non-autonomous regulation of cholinergic motor neuron synaptic structure and function. This finding is in line with recent studies showing that neuronal GPCRs, including FSHR-1 and SRZ-75, can act via inter-tissue signaling mechanisms to regulate downstream protein localization and oxidative stress responses in the intestine, muscle, and/or hypodermis [51,97] and that signals released from the intestine can impact neuronal and/or neuromuscular signaling and behavior [98,99].

While our data strongly support a model for intestinal regulation of neuromuscular function by FSHR-1, we also observed rescue of neuromuscular behaviors with *fshr-1* re-expression under pan-glial and pan-neuronal promoters and with expression in cholinergic or GABAergic neurons and even muscle, suggesting additional potential sites of *fshr-1* action and modes of regulation. These additional rescue sites suggest that FSHR-1 is a multi-tissue coordinator of NMJ function that may be further explored. However, several pieces of evidence give us pause when considering which, if any, of these additional tissue types may also be sites of endogenous FSHR-1 action on the NMJ.

First, although we found that *fshr-1* expression in *either* excitatory cholinergic or inhibitory GABAergic motor neurons was sufficient to restore muscle excitation to *fshr-1* mutants (S7 Fig), this result implies different effects of FSHR-1 signaling in these antagonistic motor neuron classes to induce similar increases in muscle contraction. Such a model is not consistent with the synaptic vesicle accumulation seen in cholinergic and, to a lesser extent, in GABAergic motor neurons, nor with the exacerbation of synaptic vesicle accumulation defects in both cholinergic and GABAergic rescue strains (Figs 2, S3 and S7). Most importantly, we and others have been unable to detect significant *fshr-1* expression in the dorsal or ventral nerve cords [27,48,49]. If *fshr-1* is expressed at low levels in motor neurons, additional studies will be required to identify and describe cell type-specific FSHR-1 signaling pathways that could mediate cell autonomous effects.

Second, our own and others’ expression data does support the potential for cell non-autonomous activity of *fshr-1* in some other distal tissues. In addition to its intestinal expression, *fshr-1* is expressed in subsets of glial cells (all six IL socket glia, S8 Fig; 48) and transcripts have been detected in head neurons, such as ASEL chemosensory neurons, CAN cells, DVB motor neurons in the defecation circuit, and PVW interneurons in the tail [27,48,49]. Either glial cells or these neurons may release neuropeptides or other molecules that act at a distance to impact neuromuscular function [100,101]. Thus, a cell non-autonomous function for *fshr-1* that impacts neuromuscular signaling would be consistent with known secretory functions of these cells. In fact, several studies have directly demonstrated the ability of FSHR-1 to participate in inter-tissue signaling to control diverse cellular processes. Cho and colleagues (2007) showed that expression of *fshr-1* in somatic tissues alone can restore fertility and germline cell fate to *fshr-1*(*0*);*fbf-1(RNAi)* animals [27]. Kim and Sieburth (2020) demonstrated that *fshr-1* expression in neuronal cells following induction of intestinal oxidative stress is sufficient to control the mitochondrial localization of the lipid kinase SPHK-1 in the intestine [51]. Despite the ability of glial or neuronal expression of *fshr-1* to restore neuromuscular behavioral phenotypes to varying extents, expression in these tissues is unable to rescue GFP::SNB-1 accumulation in the cholinergic neurons of *fshr-1* mutants (Figs 3 and S6). This further supports the hypothesis that *fshr-1* expression in these cells may not be the correct or sole location of endogenous FSHR-1 activity in controlling neuromuscular signaling. It is also possible that FSHR-1 functions in multiple tissues in addition to the intestine but that in our single tissue-specific rescue, we have not achieved the correct balance of *fshr-1* expression to restore full wild type effects on synaptic vesicles and/or neuromuscular activity. Conversely, the *mir-228p* pan-glial promoter, which is active in all glia, as well as in seam and excretory cells [102], or pan-neuronal promoters *rab-3p* or *rgef-1p*, may be driving expression of *fshr-1* in the subsets of these cells where *fshr-1* is endogenously expressed but also in additional glia, neurons, or other cells in which *fshr-1* expression is not found (S8 Fig). These sites of ectopic *fshr-1* expression may create neomorphic effects that impact the outcomes of our glial and neuronal rescue experiments. Additionally, it is possible that *fshr-1* expression in any of these tissues is increasing aldicarb sensitivity by increasing feeding and, thus, aldicarb intake; however, we think this is unlikely since such feeding effects should not impact swimming or crawling behaviors in the same way. Finally, as some prior studies have reported leaky expression of rescuing transgenes in the intestine [103,104], the rescue and/or overexpression neuromuscular phenotypes achieved with exogenous expression of *fshr-1* under glia- or neuron-specific promoters could be due to aberrant intestinal expression. Future studies testing rescue in specific subsets of glial cells [102] or head neurons, as well as experiments assessing the effects of *fshr-1* knock down in additional tissue types where *fshr-1* is expressed, should prove informative in more fully defining the endogenous tissues in which FSHR-1 acts to control muscle excitation.

### FSHR-1 regulates synaptic vesicle localization and release potentially through effects on active zone proteins in cholinergic motor neurons

We observed reduced recovery of the pH-sensitive SpH reporter after photobleaching in *fshr-1* mutants compared to wild type worms (Fig 2B). *fshr-1* mutants exhibited ~35% reduction in recovery of SpH in cholinergic motor neurons following photobleaching compared to ~70% reduction in recovery for *unc-13* mutants, consistent with a modest but incomplete reduction of muscle excitation in the *fshr-1*(*lf*) animals. We propose a model in which reduced cholinergic vesicle exocytosis is responsible for the reduced muscle excitation and consequent alterations in swimming and crawling, as well as sensitivity to aldicarb paralysis. Our results are consistent with prior findings that *fshr-1* mutants have defects in cholinergic synaptic vesicle exocytosis, as evidenced by the accumulation of the synaptic vesicle associated protein, GFP::SNB-1 in cholinergic motor neurons [50]. For some tissue-specific *fshr-1* expression experiments, we observed partial rescue of the swimming and crawling *fshr-1* mutant phenotypes without a restoration of normal synaptic vesicle localization (e.g., cholinergic motor neurons, GABAergic motor neurons, glial cells, S6 and S7 Figs). We conclude that GFP::SNB-1 accumulation may not solely report on rates of synaptic vesicle release and/or that there are compensatory mechanisms for increasing muscle excitation (e.g. upregulation of postsynaptic ACh receptors or muscle excitatory machinery). Notably, *fshr-1* re-expression under either its own promoter or an intestinal promoter (Figs 2 and 4), but not with promoters driving expression in other tissues (S6 and S7 Figs), provided rescue of both neuromuscular behaviors and GFP::SNB-1 localization. These findings underscore the physiological importance of intestinal FSHR-1 in maintaining neuromuscular activity through effects on cholinergic synaptic vesicle release.

We also observed modest, although inconsistent, increases in GFP::SNB-1 puncta intensity (~25%) and density (~15%) in GABAergic motor neurons (S3A Fig). While it is possible *fshr-1* impacts GABA neurons either directly or indirectly, the greater effect on GFP::SNB-1 accumulation in cholinergic neurons likely accounts for the overall reduction in muscle contraction observed in *fshr-1* mutants. Future experiments using more sensitive approaches will be required to determine if the trends we observed in GABAergic neurons of *fshr-1* mutants have physiological relevance.

Our quantitative imaging indicates *fshr-1* is important for localization of the active zone scaffold and synaptic vesicle priming factor, UNC-10, at cholinergic motor neuron presynapses (Fig 2C). While modest effects on GFP::SYD-2 also were observed in cholinergic and GABAergic motor neuron presynapses, there was no change in the synaptic abundance of GFP::CLA-1, indicating the specificity of *fshr-1’s* effects (Figs 2D and 2E and S3C). UNC-10 is a component of the presynaptic dense projection where it is involved in synaptic vesicle priming in conjunction with UNC-13/Munc13 [105,106]. UNC-10 also works alongside RIMB-1/RIM binding protein to promote localization of UNC-2 voltage-gated calcium channels, which are required for synaptic transmission [107]. Although a recent study also implicated Rim1/2, MUNC-13, and RAB3 in the release of DCVs from mammalian hippocampal neurons [108], DCVs appear relatively undisturbed in *unc-10* loss of function mutants [105,109], where synaptic vesicle priming is impaired. This finding is consistent with our data showing no effect of *fshr-1* loss of function on the neuropeptide and DCV marker INS-22 (Fig 2F).

The strong, cholinergic-specific effects on UNC-10, in contrast to the weaker and general accumulation of SYD-2, suggest UNC-10 may be the critical target of FSHR-1 signaling in cholinergic motor neurons. Mammalian RIM1 is known to interact with several active zone proteins, including SYD-2/Liprinα [110]. In *C*. *elegans*, UNC-10/RIM and SYD-2/Liprinα colocalize in the active zone where they work in genetic pathway to tether vesicles to the presynaptic dense projection [71,111]. Although *syd-2* mutants display disrupted UNC-10 synaptic distribution, *unc-10* is not required for SYD-2 localization to the active zone [112]. These data are consistent with our findings showing a significant accumulation of UNC-10 but more general and less robust effects on SYD-2 in *fshr-1* mutants. Nevertheless, as most studies have focused on the effects of active zone protein depletion, the effects of excess UNC-10, SYD-2, or other active zone proteins, as observed in our experiments, remain largely uncharacterized. Both UNC-10 and SYD-2 are multi-domain scaffolds that interact with numerous binding partners involved in active zone organization and synaptic vesicle release. Therefore, an improper build-up of UNC-10 and SYD-2 in and around cholinergic synapses in the absence of *fshr-1* expression could lead to aberrant synaptic docking and priming at release sites. This effect could be responsible for the accumulation cholinergic synaptic vesicles in *fshr-1* mutants. *syd-2* loss of function mutants were shown to have decreased synaptic INS-22::Venus abundance and increased dendritic and cell body INS-22::Venus fluorescence, indicating a requirement for SYD-2 in polarized trafficking of DCVs [73]. In contrast, UNC-10 has not been implicated in neuropeptide release in *C*. *elegans*. Thus, the fact that we observe no change in INS-22::Venus-labeled DCV localization in our *fshr-1* mutants, is consistent with a model in which the primary effect of FSHR-1 on muscle excitation may be via effects on UNC-10 localization to impact synaptic vesicles.

### GSA-1 and ACY-1 and SPHK-1 act downstream of FSHR-1 to control neuromuscular activity

Mammalian LGRs frequently act via Gα_S_ proteins to increase cyclic AMP (cAMP), which, in turn, activates protein kinase A (PKA) to phosphorylate targets leading to a variety of cellular effects including changes in gene expression [85,113,114]. Genetic studies implicated a GSA-1 –ACY-1 pathway downstream of FSHR-1 in *C*. *elegans* germline in development and fate specification, and *fshr-1* acts in parallel to *pmk-1* p38 Map kinase to promote resistance to pathogen infection and for expression of genes involved in innate immune responses and lipid homeostasis [27,52,53,55]. Similarly, work in mammalian epithelial ovarian cancer cells demonstrated that FSHR activates SPHK via an Erk Map kinase pathway [59]. Neuronal FSHR-1 also regulates SPHK-1 mitochondrial localization in the *C*. *elegans* intestine following intestinal stress [51,59]. Consistent with these studies and other reports showing that neuronal GSA-1, ACY-1, and SPHK-1 can all promote muscle excitation through effects to increase neurotransmitter release [88,91], our genetic epistasis data support roles for GSA-1 and ACY-1, as well as SPHK-1, downstream of FSHR-1 in controlling neuromuscular signaling (Fig 5). Additional experiments with intestine-specific RNAi demonstrate *gsa-1*, *acy-1*, and *sphk-1* are all required in the intestine for the effects of intestinal *fshr-1* overexpression on body bending rates (Fig 7A–7C), suggesting cell autonomous activation of these effectors by intestinal FSHR-1.

Despite these findings, our studies do not define whether GSA-1, ACY-1, and/or SPHK-1 also act in other distal tissues or in the motor neurons to impact UNC-10 and synaptic vesicle release in response to inter-tissue signals initiated by FSHR-1. Additionally, in the case of ACY-1, it is possible the incomplete suppression of *fshr-1(lf)* by *acy-1(gf)*, rather than being due to a weak *acy-1(gf)* allele [88], is due to the activity of other ACY family members (ACY-2, 3, or 4) acting downstream of FSHR-1 in one or more cell types. GSA-1 –ACY-1 signaling often leads to cAMP-mediated activation of PKA, and RIM1 is a phosphorylation target of mammalian PKA [115,116]. Therefore, it is tempting to speculate that PKA may also act downstream of the FSHR-1 –GSA-1 –ACY-1 signaling axis to connect this pathway to UNC-10 and ultimately to synaptic vesicle release. PKA has been shown to function downstream of GSA-1 and ACY-1 in excitatory GABAergic neurons to control expulsion in *C*. *elegans* [117]. Future work will be needed to confirm if PKA is a relevant downstream target of FSHR-1 signaling and if PKA or other molecules are intermediates between FSHR-1 signaling and the effects on UNC-10 and SYD-2. In support of this possibility, FSHR signaling through PKA in mammalian cells can lead to the activation of ERK Map Kinases, PI3 Kinases, IGF-1R phosphorylation and p38 Map kinases in granulosa cells [85].

While FSHR and other glycoprotein hormone receptors (TSHR and LHR) most commonly activate Gα_S_—adenylyl cyclase—PKA pathways, each of these receptors can also initiate signaling through other G proteins. For example, LHR has been shown to switch, upon ligand binding, from initial Gα_S_ activation to Gαi_13_ activation following prolonged stimulation [118], with corresponding changes in the cAMP levels. Alternatively, FSHR was reported to interact with Gαi upon activation by specific glycosylated FSH variants and with Gαq/11 at high FSH concentrations [85]. In the context of SPHK activation, signaling from FSHR through Erk Map kinase and SPHK-1 localization to presynaptic sites in cholinergic neurons was shown to depend upon EGL-30/Gαq and the Rac exchange factor UNC-73/TRIO [59,91]. Finally, effects of FSHR-1 on other synaptic vesicle associated factors may affect the localization of synaptic vesicles and additional active zone proteins. A recent study showed that FSHR acts via a cAMP pathway to increase the transcription and protein expression of SNAP-25 and synaptotagmin VII in mouse ovarian granulosa cells following stimulation with pregnant mare serum gonadotropin or FSH [119]. Additional tests with cell type-specific loss- and gain-of-function alleles of the genes encoding these and other G proteins, lipid signaling effectors, and other candidate signaling components will be needed to fully characterize the direct downstream FSHR-1 pathway.

### The thyrostimulin-like glycoprotein subunit orthologs GPLA-1 and GPLB-1 regulate NMJ function as likely FSHR-1 ligands

Recent work implicated the GPLA-1/GPA2 and GPLB-1/GPB5 thyrostimulin-like subunits as FSHR-1 ligands in the regulation of body size [48]; other results demonstrated a similar role for GPLA-1 with FSHR-1 in controlling phenoptosis and growth but did not test GPLB-2 [55,56]. Our results add to the list of processes regulated by GPA2/GPB5 signaling in conjunction with FSHR-1. We found that *lf* mutations in either *gpla-1*, *gplb-1*, or *fshr-1* all showed similar levels of reduction in neuromuscular behaviors and these effects were non-additive when tested in double or triple mutant combinations (Fig 6). These results support a model in which both α and β subunits are required for receptor activation. This finding matches *in vivo* results for body size regulation, but differs from *in vitro* results showing either subunit alone, or both in combination, can increase FSHR-1 receptor activation [48]. The non-overlapping cellular expression of *gpla-1* and *gplb-1* and biochemical pulldown results demonstrating that GPLA-1 and the extracellular domain of FSHR-1 can co-precipitate in the absence of excess GPLB-1 also support the predicted potential for independent action of these ligands [48,56,95,120]. While our results suggest that both GPLA-1 and GPLB-1 are required for FSHR-1 neuromuscular regulation, it will be of interest to determine if they have independent roles in other physiological contexts, such as during oxidative stress, and if there are additional FSHR-1 ligands. Finally, ligand-independent constitutive activation of FSHR-1 has been reported [48,86]. While our data indicate that both GPLA-1 and GPLB-1 ligands are specifically required for the effects of intestinal FSHR-1 overexpression on neuromuscular function in *C*. *elegans* (Fig 7D–7E), future work assessing potential roles for constitutive FSHR-1 activation in the context of neuromuscular regulation and in other physiological processes will be important for gaining a complete picture of FSHR-1 activity.

### Conclusions and future directions

Overall, our data are consistent with a cell non-autonomous role for the conserved GPCR FSHR-1 in promoting muscle excitation in *C*. *elegans*. Our data suggest that FSHR-1 acts in intestinal cells and potentially in other distal tissues upstream of GSA-1, ACY-1, and SPHK-1 signaling to ultimately regulate active zone protein localization and synaptic vesicle release from cholinergic motor neurons (Fig 8). While we find that FSHR-1 can function in multiple cell types to restore muscle contraction to *fshr-1* mutants, our collective data provide significant support for cell non-autonomous activity of intestinal FSHR-1 in controlling neuromuscular function, likely by promoting the release of one or more inter-tissue signaling molecules. Recent work has demonstrated the existence of such cross-tissue signaling mechanisms, such as neuron-gut and glia-neuron, and some of the secreted factors involved in these processes are beginning to be described [51,97,98,100,121]. Although additional studies are required to completely define the signaling and secretory pathways involved in mediating FSHR-1 regulation of the NMJ, our work defines a previously unappreciated pathway for inter-tissue regulation of neuromuscular function that has clear implications for coordination of organismal responses to physiological stressors. FSHR-1 has been shown to act in multiple tissues to control processes ranging from germline development to oxidative stress and pathogen resistance to phenoptosis and organism growth. Our data are consistent with the emerging role of FSHR-1 as a central inter-tissue regulator in *C*. *elegans*. Future studies investigating the relevant ligands of FSHR-1 in diverse contexts, as well as connections between roles of FSHR-1 in neuronal function, stress responses, and development will be critical for a complete picture of FSHR-1 activity. This work will also provide novel avenues to explore regarding glycoprotein hormone receptor function in mammals. Such studies will ultimately contribute to our understanding of GPCR biology and neuronal signaling imbalances in neurological diseases.

## Materials and methods

### Strains and strain maintenance

*C*. *elegans* strains used in this study include those listed in Table 1. All strains were grown on 6 cm plates containing nematode growth medium (NGM) agar spotted with ~300 μL of OP50 *E*. *coli* at 20°C using standard protocols described previously [122]. Young adult hermaphrodites were used for all experiments.

**Table 1:** *C*. *elegans* strains used in this study. (*) indicates number of backcrosses to N2.

Strain Number	Genotype	Reference
N2	N2	
RB911	*fshr-1*(*ok778*)	*C*. *elegans* Deletion Mutant Consortium [123]
JRK165	*fshr-1*(*ok778*)*6	This study
KG522	*acy-1*(*md1756*)	Cho et al (2007) [27]
WY371	*acy-1*(*md1756*); *fshr-1*(*ok778*)	Cho et al (2007) [27]
KG421	*gsa-1*(*ce81*)	Cho et al (2007) [27]
WY415	*gsa-1*(*ce81*); *fshr-1*(*ok778*)	Cho et al (2007) [27]
VC916	*sphk-1*(*ok1097*)	*C*. *elegans* Deletion Mutant Consortium [123]
JRK166	*sphk-1*(*ok1097*)*4	This study
JRK168	*sphk-1*(*ok1097*)*4; fshr-1(ok778)*6	This study
KP3814	*nuIs152* (P*unc-129*::*SNB- 1*::*GFP*)*2	Sieburth et al (2005) [27]
JRK42	*nuIs152; fshr-1*(*ok778*)*3	This study
KP3928	*nuIs165* (*Punc-129*::*UNC-10*::*GFP*)	Sieburth et al (2005) [27]
JRK76	*nuIs165; fshr-1(ok778)*	This study
KP3091	*nuIs159* (*Punc-129*::*SYD-2*::*GFP*)*10,	Sieburth et al (2005) [27]
JRK67	*nuIs159; fshr-1*	This study
TV18676	*wyIs687* (*Punc-17*::*CLA-1*::*GFP*)	P. Kurshan
JRK124	*wyIs687; fshr-1*	This study
KP3894	*nuIs195* (*Punc-129*::*ins-22*::*VENUS*)	Sieburth et al (2005) [50]
JRK96	*nuIs195; fshr-1*(*ok778*)	This study
MT20435	*nIs463 [Punc-17*::*snb-1*::*SEP(SpH)*]	Paquin et al (2016) [65]
JRK164	*nIs463; fshr-1*(*ok778*)*3	This study
JKR181	*nIs463; unc-13* (*s69*)	This study
IBE89	*ibtEx15 [fshr-1p*::*fshr-1gDNA*::*sl2*::*mKate 10 ng/μl; unc-122p*::*gfp 25 ng/μl]; fshr-1*(*ok778*)	Kenis et al (2023) [48]
IBE225	*ibtEx35 [ges-1p*::*fshr-1 cDNA*::*sl2*::*GFP 30 ng/μl; myo-2p*::*mCherry 10 ng/μl*]; *fshr-1(ok778)*	Kenis et al (2023) [48]
IBE333	*ibtEx51 [mir-228p*::*fshr-1 cDNA*::*sl2*::*mKate 20 ng/μl; unc-122p*::*GFP 10 ng/μl]; fshr-1(ok778)*	Kenis et al (2023) [48]
IBE223	*ibtEx34[rab-3p*::*fshr-1 cDNA*::*sl2*::*mKate 20 ng/μl; unc-122p*::*gfp 25 ng/μl]; fshr-1 (ok778)*	Kenis et al (2023) [48]
IBE465	*ibtEx67[rgef-1p*::*fshr-1 cDNA*::*sl2*::*GFP*::*tbb-2 3’UTR 40 ng/μl; myo-2p*::*mCherry 10 ng/μl]; fshr-1 (ok778)*	Kenis et al (2023) [48]
IBE88	*gpla-1*(*ibt1*)*4	Kenis et al (2023) [48]
JRK183	*ibtEx15 [fshr-1p*::*fshr-1gDNA*::*sl2*::*mKate 10 ng/μl; unc-122p*::*gfp 25 ng/μl]—*outcrossed from IBE89	This study
JRK94	*agEx43* [P*fshr-1*:: *fshr-1; Pmyo-2*:: *NLS*:: *cherry*]*; fshr-1(ok778); nuIs152*	This study
JRK167	*ibtEx35 [ges-1p*::*fshr-1 cDNA*::*sl2*::*GFP 30 ng/μl; myo-2p*::*mCherry 10 ng/μl*]; *fshr-1(ok778); nuIs152*	This study
JRK177	*ibtEx35 [ges-1p*::*fshr-1 cDNA*::*sl2*::*GFP 30 ng/μl; myo-2p*::*mCherry 10 ng/μl*]—outcrossed from IBE225	This study
JRK182	*ibtEx51 [mir228p*::*fshr-1 cDNA*::*sl2*::*mKate 20 ng/μl; unc-122p*::*GFP 10 ng/μl]—*outcrossed from IBE333	This study
IBE208	gplb-1(ibt4)*4	Kenis et al (2023) [48]
IBE149	*gpla-1(ibt1) V; fshr-1(ok778) V*	Kenis et al (2023) [48]
IBE420	*gplb-1 (ibt4) V; fshr-1(ok778) V*	Kenis et al (2023) [48]
IB265	*gpla-1(ibt1) V; gplb-1(ibt4) V*	Kenis et al (2023) [48]
IB421	*gpla-1(ibt1) V; gplb-1 (ibt4) V; fshr-1(ok778) V*	Kenis et al (2023) [48]
VP303	*kbIs7*;*rde-1(ne219)*	Espelt et al (2005) [84]
JRK186	*ibtEx35; kbIs7;rde-1(ne219)*	This study
JRK189	*ibtEx67[rgef-1p*::*fshr-1 cDNA*::*sl2*::*GFP*::*tbb-2 3’UTR 40 ng/μl; myo-2p*::*mCherry 10 ng/μl]—*outcrossed from IBE465	This study
JRK192	ibtEx35; gpla-1(ibt1)*6	This study
JRK197	ibtEx35; gplb-1(ibt4)*4	This study
JRK199	ibtEx35; gpla-1(ibt1)*6; gplb-1(ibt4)*4	This study

### RNA interference (RNAi)

Intestine-specific knockdown of *fshr-1* expression was achieved using the feeding RNA interference (RNAi) protocol described previously [124]. Briefly, to prepare RNAi plates, 35 mm NGM agar plates containing 50 μg/mL Ampicillin (Amp) and 5 mM IPTG were made and allowed to dry overnight at room temperature. Overnight cultures containing HT115(DE) bacteria carrying the empty L4440 RNAi plasmid or HT115(DE) bacteria carrying the L4440 plasmid containing a fragment of the *fshr-1* gene were prepared in Luria Broth (LB) containing 50 μg/mL Amp and grown overnight at 37°C [124]. The next day, the RNAi plates were spotted with 150–200 μl of either the L4440 or *fshr-1* RNAi cultures and allowed to dry open for about two hours then closed, then dried overnight on the benchtop. On day three, two or three L4440 larval stage 4 (L4) worms of the intestine-specific RNAi strain VP303 [84] were placed onto each plate and left for four days at 20°C until they reached young adult stage.

### Aldicarb assays

NGM agar plates (35 mm) containing 1 mM aldicarb (Sigma-Aldrich) were prepared, then spotted with 150 μL of OP50 *E*. *coli*. After one day, approximately 20 worms were placed on each plate in 2-minute intervals, then measured for total paralysis every 25 minutes. Total paralysis was defined as no physical movement from the worms when prodded three times with a platinum wire on the nose [61]. Three plates were tested for each strain of worms per experiment with the experimenter double-blinded to genotype. The average percentage of worms paralyzed for each strain at each time point +/- s.e.m. was calculated using Microsoft Excel. Data from a total of 8–12 plates were pooled from experiments taken at the same time intervals over several days. Statistical analyses were performed using JMP 14 software R version 2022.02.0+443 to compare the average percentages of worms of each strain paralyzed at the timepoint with the largest differences for each experiment (80 or 100 minutes). All data were first confirmed to fall within a normal distribution, and equality of variances confirmed. For experiments in which all data fell within a normal distribution, one-way ANOVAs then were used to assess statistical significance of the differences in the means between groups (α < 0.05), followed by Tukey’s post hoc test (α < 0.05 for all). *p* value results of ordered difference reports are provided. Non-parametric Wilcoxon Rank Sums test with one-way Chi square approximation, followed by a Steel-Dwass post-hoc test for multiple comparisons (α< 0.05 for all).

### Swimming assays

Thirty worms of each strain were picked onto a clean plate containing OP50. Plates were double blinded to genotype. Then, 100 μL of M9 (22 mM KH_2_PO_4_, 42 mM Na_2_HPO_4_, 86 mM NaCl) buffer was put into a single well on a 96-well plate. An individual worm was placed onto an unspotted NGM agar plate at room temperature for 1 minute. The worm was then placed into the well with the M9 buffer and left to acclimate for 1 minute. Body bends were recorded for 30 seconds following the acclimation period, then multiplied by 2 to obtain body bends/minute. One body bend was counted as one full body bend to one side, followed by a return to the center position [57,125]. Swimming experiments were performed 3–6 times with 30 animals per strain tested per day for genetic mutant analyses and 5–12 times with 10 animals per treatment tested per day for RNAi studies and daily averages taken per strain. The daily averages were then confirmed to fall within a normal distribution and to exhibit equality of variances (variances differ by no more than 10x) using R version 2022.02.0+443. Finally, the statistical significance of differences between the strains was determined using a one-way ANOVA followed by a Tukey post-hoc test (α < 0.05).

### Multi-Worm Tracking

Quantification of body bending was measured using the Multi-Worm Tracker (Rex Kerr, https://sourceforge.net/projects/mwt/). Each individual multi-worm tracking experiment was conducted with 20 staged 1-day old adult animals on Bacto-agar NGM agar plates seeded with a thin lawn of OP50 E. coli (50 μl). Experiments were analyzed using custom MATLAB (The MathWorks, Inc.) scripts to interface with the Multi-Worm Tracker feature extraction software Choreography [126]. Statistical analysis was performed in GraphPad Prism.

### Quantitative fluorescence imaging of synaptic markers

Wild type and *fshr-1* mutant worms carrying fluorescently tagged transgenes (GFP::SNB-1, GFP::UNC-10, mCherry::UNC-10, GFP::SYD-2, GFP::CLA-1, or INS-22:VENUS) in subsets of dorsal motor neurons were grown until the young adult stage [50,70–72]. Worms were paralyzed in 30 mg/ml butanedione monoxime (BDM, Sigma-Aldrich) in M9 on No. 1.0 coverslip (VWR #48366–067) and mounted on 2% agarose pads. For widefield images (Figs 2, S3 and S7), Z-series stacks of the dorsal nerve cord (DNC) of worms halfway between the vulva and the tail were taken using a Leica DMLB compound fluorescence microscope with Exi Aqua cooled CCD camera at 100x/1.4 NA magnification every 0.2 μm over a 1 μm depth. Exposure settings and gain were set to fill a 12-bit dynamic range without saturation. These settings were identical for all images taken of a given fluorescent marker [i.e., GFP::SNB-1 in cholinergic (*nuIs152*) neurons] [127]. Maximum intensity projections were compiled from the z-stacks. Linescans of dorsal nerve cord puncta in these projections were generated using Metamorph (v7.1) software, and the linescan data were analyzed with Igor Pro (Wavemetrics) using custom written software as previously described [128]. Mercury arc lamp output was normalized by measuring the intensities of 0.5 μm FluoSphere beads (Invitrogen Life Technologies) for each imaging day. Puncta intensities were calculated by dividing the average maximal peak intensity by the average bead intensity for the corresponding day. Puncta densities were determined by quantifying the average number of puncta per 10 μm of the dorsal nerve cord. For all data, an average of the values for each worm in the data set ± s.e.m. is shown. Statistical significance of any differences between wild type and *fshr-1* values was determined using a Student’s *t* test or One-way ANOVA with Tukey’s post hoc test, as appropriate (*p* < 0.05). For confocal images (Figs 4 and S6), worms were immobilized using 30 mg/mL BDM in M9 on a No. 1.5 coverslip (VWR #48366–227) and mounted onto a glass slide containing a 2% agarose pad. Imaging was performed on a Nikon Ti2E Yokogawa CSU-X1 Spinning Disk Field Scanning Confocal Microscope equipped with Nikon Elements software. The worms were found and marked using a 10x EC Plan-Neofluar 10x/0.30 NA objective and then imaged using a 100x/1.4NA Plan-Apochromat objective. Images of the dorsal nerve cord halfway between the vulva and the tail were taken using the 488 nm laser microscope set to 26.9% power. A 100 ms exposure time and 1x1 binning were used for focusing, 300 ms exposure, and 1x1 binning for image acquisition. Images were taken over a total depth of 1*μ*m, with a step size of 0.1*μ*m for a total of 11 planes, which were compiled to make a single maximum-intensity projection. Approximately 20–30 maximum intensity projection images, one image per worm, were obtained for each strain. On a single day of imaging, at least three images of each strain were obtained to account for daily variation. Confocal puncta characterization was performed on maximum intensity projections using the Fiji puncta analysis platform [63,129] with the following settings: minimum puncta size = 0.3, sigma = 0.75, radius = 1, method = Phansalkar. For all quantitative imaging data, graphs of puncta intensities show data normalized to wild type values. Representative images were processed in Adobe Photoshop by adjusting levels, cropping images, and converting images from.tif files into.jpeg files. All processing was done identically and uniformly for all images from a given experiment. Representative images were finalized in Microsoft PowerPoint by adjusting sharpness and contrast for clarity in figures. All adjustments were made uniformly for all figures.

### Quantification of synaptic vesicle release

Dorsal nerve cords between the vulval and tail were imaged in wild type, *fshr-1*(*ok778*), and *unc-13*(*s69*) animals expressing SNB-1::Superecliptic pHluorin (SpH) in acetylcholine (ACh)-releasing neurons (*Punc-17*::*SpH*) [63]. Animals were immobilized on 5% agarose pads in 30 mg/mL BDM in M9 buffer and imaged with 100x/1.4NA on a Nikon Ti2-E inverted microscope equipped with a Yokogawa CSU-X1 spinning disk head, an OptiMicroScanner for photostimulation, and a Hamamatsu ORCA fusion camera. Imaging was performed using the CSU-X1 488nm laser at 8% power and photobleaching by the 405 nm FRAP laser at 1% power (40 μsec dwell time, 70 μsec duration). Regions of interest (ROIs) outlining single SpH puncta fluorescence along the dorsal axon were bleached (S3 Fig). Time-lapse images of a single plane with these ROIs in focus were taken over a period of 60 seconds, 10 seconds pre-bleach and 50 seconds post-bleach. Mean fluorescence intensity within the ROI was tracked for each of the two bleached spots, as well as for a control reference synaptic ROI within the nerve cord and for a background ROI outside of the cord. Multiple sections of the dorsal cord (typically 2–4) were imaged in this fashion from each animal. Intensity data for all ROIs was exported to Microsoft Excel and the background ROI was subtracted from the bleached and reference ROIs. The background-normalized intensity values were visualized in Igor Pro 9.0.1.2. Percent recovery after photobleaching was determined using the following equation using pre-bleach (t = 10 sec), post-bleach (t = 10.2 sec), and post-recovery (50 sec post-bleach, t = 60 sec) intensities [130]:

$$\text{Percent}\;\text{Recovery}=\frac{\left[\left(\text{Post-recovery}\;\text{Intensity}\right)\text{-}\left(\text{Bleached}\;\text{Intensity}\right)\right]}{\left[\left(\text{Pre-bleach}\;\text{Intensity}\right)\text{-}\left(\text{Bleached}\;\text{Intensity}\right)\right]}\text{X}\;100\%$$

The mean of the percent recovery from the different ROIs was calculated for every animal. One-way ANOVA and Tukey’s post hoc tests were used to compare the means of the datasets following tests for normality and equality of variance (*p* < 0.05) in R version 2022.02.0+443 as described above.

## Supporting information

S1 Fig Rescue of *fshr-1* neuromuscular defects by additional independent *fshr-1* transgenes. Aldicarb paralysis assays and swimming assays were performed on wild type worms, *fshr-1(ok778)* mutants, and rescued animals re-expressing *fshr-1* under either (A) the endogenous *fshr-1* promoter (P*fshr-1*, *fdEx41*), (B) an intestinal promoter (P*ges-1*, *agIs35*) or (C) a pan-neuronal promoter (P*ric-19*, *agEx52*) in the *fshr-1* mutant background. (A-B) (*Left panels*) Representative aldicarb assays showing the percentage of worms paralyzed on 1mM aldicarb ± s.e.m. for n = 3 plates of approximately 20 young adult animals each per strain. (*Center panels*) Bar graphs showing cumulative data ± s.e.m. pooled from 3–4 independent experiments for worms paralyzed at the timepoint indicated by an asterisk (*) in the upper panels. Scatter points show individual plate averages. (*Right panels*) Box and whisker plots showing mean body bends per minute from swimming assays performed on n = 30 young adult animals of each genotype. Statistical significance of the data was analyzed using a one-way ANOVA and Tukey’s post hoc test or a Wilcoxon Rank Sum test followed by a Steel-Dwass multiple comparison analysis, as appropriate. Results of analyses for which *p* ≤ 0.05 are indicated by horizontal lines above the bars. *p ≤ 0.05, **p ≤ 0.01, ***p ≤ 0.0001.
(PDF)

S2 Fig Single-worm tracking of *fshr-1* mutants demonstrates locomotion defects. Individual wild type and *fshr-1(ok778)* mutants were tracked and analyzed using the Single Worm Tracker during 5 minutes of movement in the presence of food. Each data point in the scatterplots represents the mean measurement for a single animal from 5 min of locomotion. The following movement features were extracted: (A) head bending; (B) crawling speed; and (C) foraging speed. Red lines indicate the means of the datasets; the middle 50% (green/orange shading) and outer quartiles (gray/purple shading) are shown. Student’s *t* test (*p ≤ 0.05).
(PDF)

S3 Fig Loss of *fshr-1* has minimal effects on synaptic vesicle or active zone protein localization in GABAergic motor neurons. (A) Wild type worms and *fshr-1*(*ok778*) mutants that also expressed GFP::SNB-1 in GABAergic (GABA) neurons were imaged using a 100x objective. (*Left panel*) Representative images of the dorsal nerve cords halfway between the vulva and the tail of young adult animals. (*Right panels*) Quantification of puncta (synaptic) intensity and puncta density (per 10 *μ*m) ± s.e.m for n = 25 wild type and n = 31 *fshr-1*. Puncta intensity is shown normalized to wild type. (B-C) Wild type or *fshr-1*(*ok778*) mutant animals that also expressed (B) mCherry::UNC-10 or (C) GFP::SYD-2 in GABAergic neurons were imaged using a 100x objective. (*Upper panels*) Representative images of the dorsal nerve cords halfway between the vulva and the tail of wild type and *fshr-1* young adult animals. (Lower panels) Quantification of puncta (synaptic) intensity and puncta density (per 10 *μ*m) ± s.e.m. Puncta intensity is shown normalized to wild type. For (B), n = 26 for wild type, n = 27 for *fshr-1*. For (C), n = 17 for wild type, n = 20 for *fshr-1*. Student’s *t* tests were used to compare the means of the datasets. *p ≤ 0.05 are shown.
(PDF)

S4 Fig Fluorescence recovery after photobleaching of SNB-1::SpH in cholinergic motor neurons. (A) Representative images of pre-bleach(a), post-bleach(b), and post-recovery(c) of SNB-1::SpH labeled vesicles in the dorsal nerve cords in wild type animals expressing SNB-1::SEP in cholinergic motor neurons (*Punc-17*). Yellow circle marks the ROI of a single SpH punctum. (B) Plot profile of the indicated ROI is shown, indicating the points of measurements of pre-bleach, post-bleach and post-recovery used in calculating % recovery (described in *Materials and Methods*).
(PDF)

S5 Fig Levamisole sensitivity of *fshr-1* mutants is rescued by re-expression of *fshr-1* in known sites of *fshr-1* expression but is exacerbated by re-expression in non-endogenous muscle expression sites. (A-C, E) Box and whisker plots showing results of levamisole paralysis assays performed on wild type and *fshr-1(ok778)* mutant animals, as well *fshr-1* mutants re-expressing *fshr-1* (Rescue) in the indicated tissues (A, intestinal *Pges-1*, *ibtEx35*; B, glial *Pmir-228 ibtEx51*; C, neuronal *Prab-3 ibtEx34*; E, muscle *Pmyo-3*, *kjrEx39*). Worms were exposed on plates containing 200μM levamisole for 100 minutes and paralysis was assessed by nose tap. n = 9 plates of approximately 20 young adult animals per plate per strain were tested. (D) Box and whisker plots of swimming experiment data repeated at least twice with *fshr-1(ok778)* mutants with muscle-specific *fshr-1* re-expression. Note that muscle rescue, unlike intestinal, glial, or neuronal rescue, caused increased body bending rates that did not coincide with any restoration of wild type levamisole sensitivity, as seen with the other rescuing transgenes. One-way ANOVA and Tukey’s post hoc tests were used to compare the means of the datasets (*p ≤ 0.05, ** *p* ≤ 0.01, ***p ≤ 0.001; n.s., not significant).
(PDF)

S6 Fig Glial and neuronal *fshr-1* re-expression also fail to rescue synaptic vesicle accumulation defects. Dorsal nerve cords of wild type worms, *fshr-1(ok778)* mutants, and animals re-expressing *fshr-1* under a pan-glial promoter (A; P*mir-228*, *ibtEx51*) or a pan-neuronal promoter (B; P*rab-3*, *ibtEx34*) also expressing GFP::SNB-1 in cholinergic neurons were imaged halfway between the vulva and the tail of young adult animals. (*Left panels*) Representative images. (*Right panels*) Quantification of normalized mean puncta (synaptic) intensity and puncta density (per 10 *μ*m) ± s.e.m. Scatter points show individual worm means (n = 21–36 animals per genotype). One-way ANOVA and Tukey’s post hoc tests were used to compare the means of the datasets (*p ≤ 0.05, ** *p* ≤ 0.01, ***p ≤ 0.001).
(PDF)

S7 Fig *fshr-1* re-expression in cholinergic and GABAergic neurons is sufficient to restore neuromuscular function but exacerbates synaptic vesicle accumulation defects. Behavioral (A, C) and synaptic structure (B, D) effects of genomic *fshr-1* DNA re-expression in the cholinergic (ACh) neurons (A, B) or GABAergic neurons (C, D) of *fshr-1(ok778)* mutant animals compared to wild type and *fshr-1(ok778)* worms. (A, C) (*Upper panels*) Representative (*left*, n = 3 plates/strain) and cumulative pooled (*right*) aldicarb data ± s.e.m. showing complete and even hyper-rescue of aldicarb paralysis in worms with cholinergic neuron-specific *fshr-1* rescue (ACh Neuron Rescue) (A) and nearly complete rescue of wild type paralysis in worms with GABAergic neuron-specific rescue (GABA Neuron Rescue) (B). Scatter points show individual plate averages taken at the timepoint indicated by the asterisk (*). (*Lower panels*) Box and whisker plots showing mean body bends per minute from swimming assays performed on n = 30 young adult animals of each genotype. Minima and maxima (whiskers) are shown, as well as the first and third quartiles of data (boxes), divided by the median line. The “X” denotes the mean value of the data set, and circles show individual data points. Note that while the GABA rescue worms have body bending rates that are partially restored to wild type levels as seen in the swimming assay, ACh rescue worms retain the reduced body bending rates seen with *fshr-1* mutants, likely due to the excessive muscle excitation caused by *fshr-1* re-expression to above wild type levels (see *Upper panels in* A vs. C). (B, D) Wild type worms, *fshr-1*(*ok778*) mutants, and ACh neuron rescue (B) or GABA neuron rescue (D) animals that also expressed GFP::SNB-1 in cholinergic (ACh) neurons were imaged using a 100x objective. (*Left panels*) Representative images of the dorsal nerve cords halfway between the vulva and the tail of young adult animals. (*Right panels*) Quantification of puncta (synaptic) intensity and puncta density (per 10 *μ*m) ± s.e.m. Puncta intensity is shown normalized to wild type. For (B), n = 29 animals imaged for wild type, n = 26 for *fshr-1*, and n = 32 for ACh Neuron rescue. For (D), n = 21 for wild type, 15 for *fshr-1*, and n = 24 for GABA Neuron rescue. For all data, statistical significance was analyzed using a one-way ANOVA and Tukey’s post hoc test or a Wilcoxon Rank Sum test followed by a Steel-Dwass multiple comparison analysis, as appropriate. Results of analyses for which *p* ≤ 0.05 are indicated by horizontal lines above the bars. *p ≤ 0.05, **p ≤ 0.01, ***p ≤ 0.001, ****p ≤ 0.001.
(PDF)

S8 Fig *fshr-1* is expressed in a subset of glial cells. Representative maximum intensity projections of young adult hermaphrodites co-expressing genomic *fshr-1* DNA under its own promoter (*Pfshr-1*, magenta) and markers of various subsets of glial cells (green) imaged in the head and tail regions where glial reside. (A) Pan-glial expression (*Pmir-228*) shows some colocalization (white, composite) with *fshr-1*, whereas (B) complete co-localization is seen with *fshr-1* and a marker of the six IL socket (ILso) glia *(Pgrl-18*). No colocalization occurs between *fshr-1* and markers of (C, D) AM and PH socket (AMso and PMso) glia (*Pgrl-2*), (E, F) AM and PH sheath (AMsh and PHsh) glia (*PF16F9*.*3*), or (G) CEP sheath (CEPsh) glia. Colocalization was confirmed by matching single planes from the ~25 μm stacks used to create the maximum intensity projects shown here.
(PDF)

S1 Text Supplemental Methods and References. (DOCX)

S1 Data Raw Aldicarb Data Compiled for Figs 1A, 4A, 5A and 5B, S1A, S1B, S1C, S7A and S7C. (XLSX)

S2 Data Raw Representative Aldicarb Data for Figs 1A, 4A, 5A and 5B, S1A, S1B, S1C, S7A and S7C. (XLSX)

S3 Data Raw Genetic Mutant Swimming Assay Data for Figs 1B, 3A–3C, 5A–5C, 6A–6D and 7D–7F. (XLSX)

S4 Data Raw Worm Tracking Data for Figs 1C, 3D and 6E and S2. (XLSX)

S5 Data Raw Quantitative Synaptic Imaging Fiji Data for Figs 4C, S6A and S6B. (XLSX)

S6 Data Quantitative Imaging Igor Analysis Summary for Figs 2A, 2C–2F, S3A–S3C, S7B and S7D. (DOCX)

S7 Data Raw SEPhluorin FRAP Imaging Data for Fig 2B. (XLSX)

S8 Data Raw RNAi Swimming Data for Fig 4B. (XLSX)

S9 Data Raw Supplemental Strain Swimming Data for S1A–S1C, S7A and S7C Figs. (XLSX)

S10 Data Raw Levamisole and Muscle Swimming Rescue Data for S5A–S5E Fig. (XLSX)
