# Supplementary figures and images for: Cell non-autonomous signaling through the conserved C. elegans glycoprotein hormone receptor FSHR-1 regulates cholinergic neurotransmission
Source: PLoS Genet. 2024 Nov 19;20(11):e1011461. doi: 10.1371/journal.pgen.1011461 (PMC11614273; doi:10.1371/journal.pgen.1011461)

Supplemental Figure 1

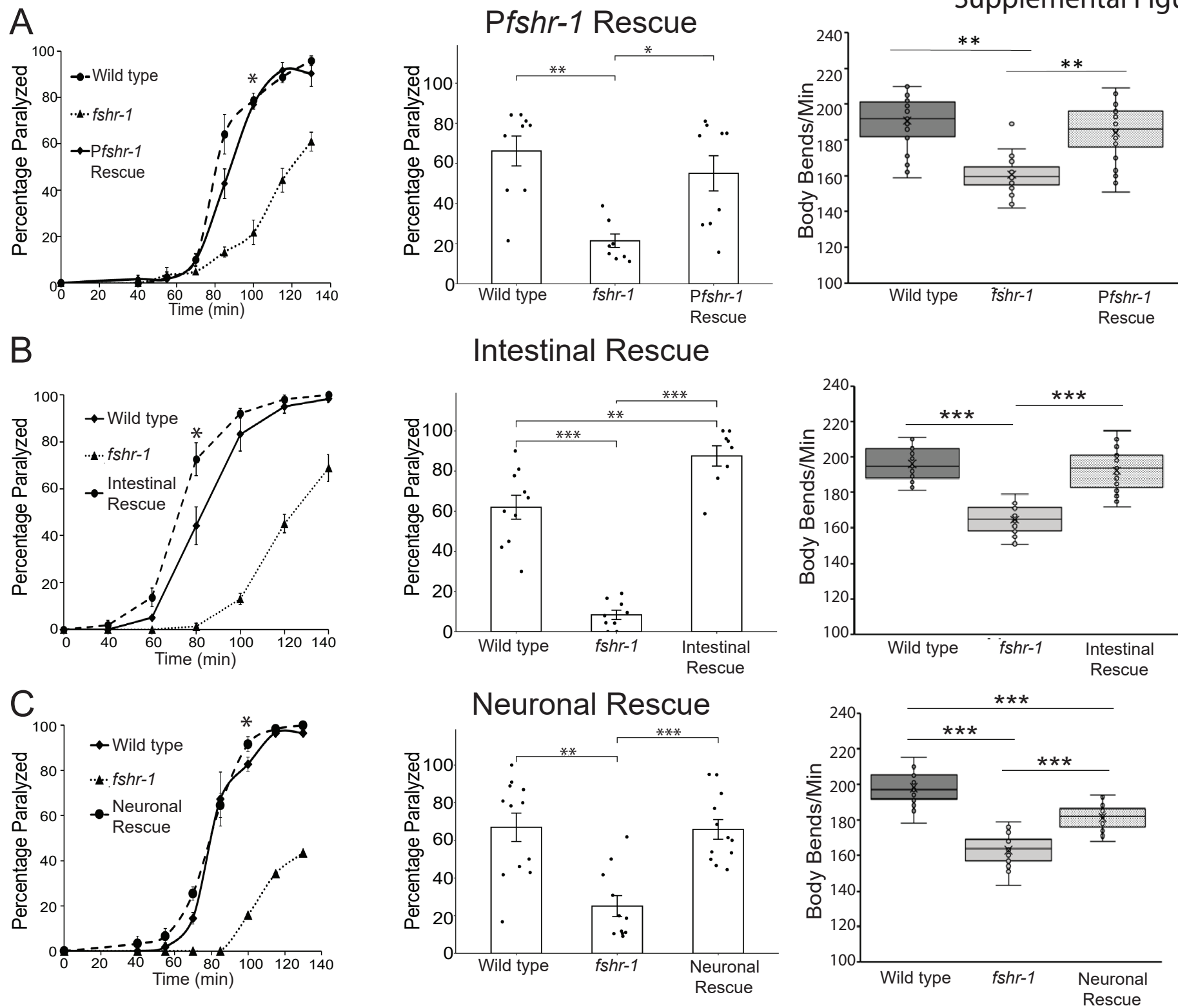

Supplement: S1 Fig — Aldicarb paralysis assays and swimming assays were performed on wild type worms, fshr-1(ok778) mutants, and rescued animals re-expressing fshr-1 under either (A) the endogenous fshr-1 promoter (Pfshr-1, fdEx41), (B) an intestinal promoter (Pges-1, agIs35) or (C) a pan-neuronal promoter (Pric-19, agEx52) in the fshr-1 mutant background. (A-B) (Left panels) Representative aldicarb assays showing the percentage of worms paralyzed on 1mM aldicarb ± s.e.m. for n = 3 plates of approximately 20 young adult animals each per strain. (Center panels) Bar graphs showing cumulative data ± s.e.m. pooled from 3–4 independent experiments for worms paralyzed at the timepoint indicated by an asterisk (*) in the upper panels. Scatter points show individual plate averages. (Right panels) Box and whisker plots showing mean body bends per minute from swimming assays performed on n = 30 young adult animals of each genotype. Statistical significance of the data was analyzed using a one-way ANOVA and Tukey’s post hoc test or a Wilcoxon Rank Sum test followed by a Steel-Dwass multiple comparison analysis, as appropriate. Results of analyses for which p ≤ 0.05 are indicated by horizontal lines above the bars. *p ≤ 0.05, **p ≤ 0.01, ***p ≤ 0.0001. (PDF) [file pgen.1011461.s001.pdf]

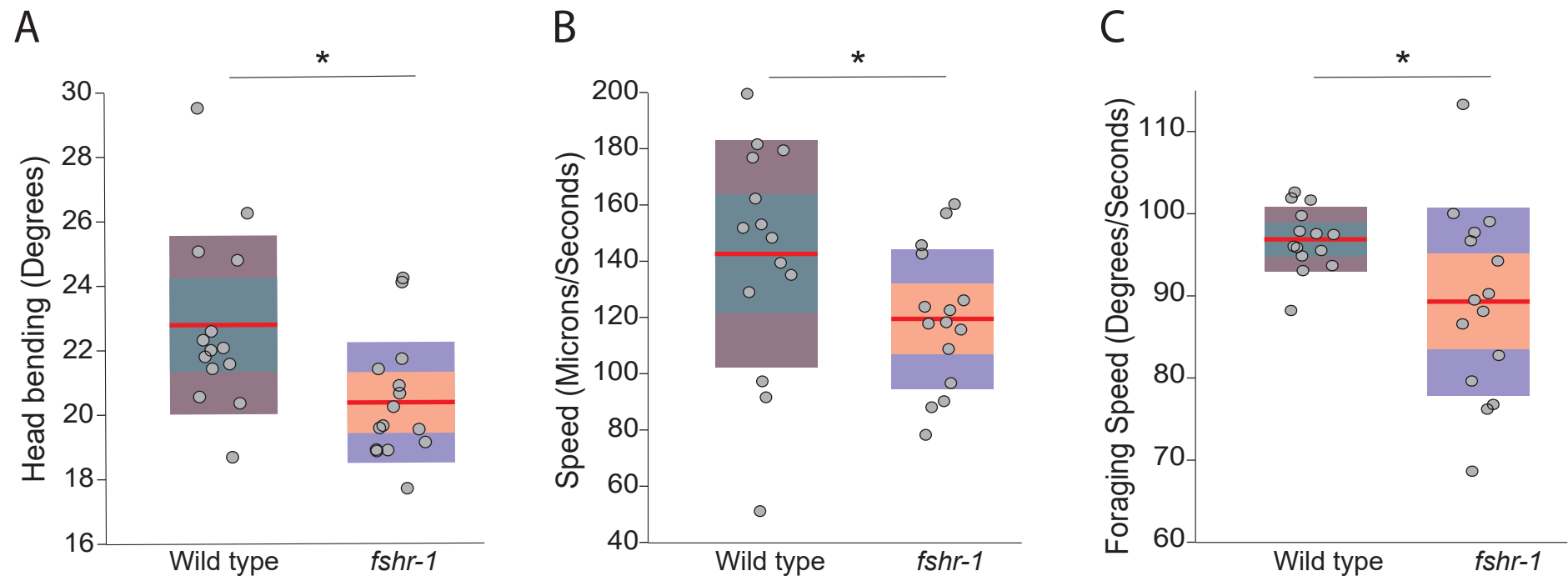

Supplement: S2 Fig — Individual wild type and fshr-1(ok778) mutants were tracked and analyzed using the Single Worm Tracker during 5 minutes of movement in the presence of food. Each data point in the scatterplots represents the mean measurement for a single animal from 5 min of locomotion. The following movement features were extracted: (A) head bending; (B) crawling speed; and (C) foraging speed. Red lines indicate the means of the datasets; the middle 50% (green/orange shading) and outer quartiles (gray/purple shading) are shown. Student’s t test (*p ≤ 0.05). (PDF) [file pgen.1011461.s002.pdf]

A

GFP::SNB-1 in GABA Neurons

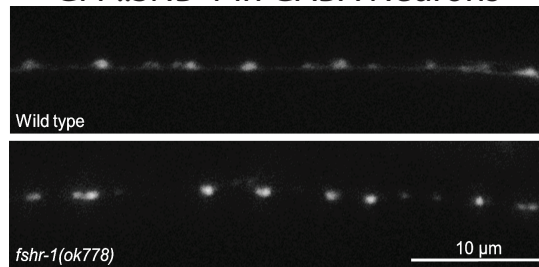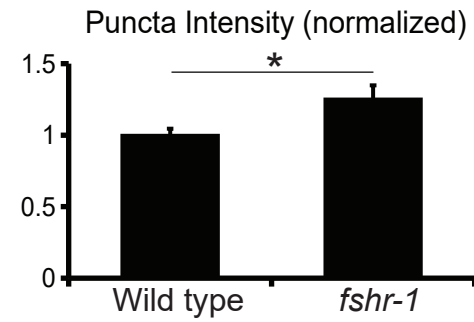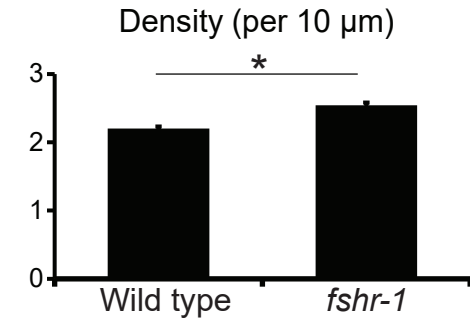

B mCherry::UNC-10 in GABA Neurons

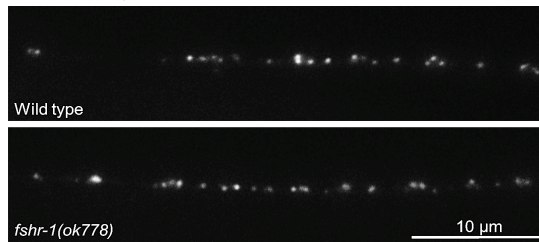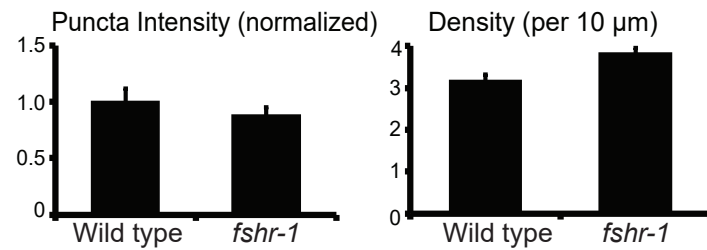

C

GFP::SYD-2 in GABA Neurons

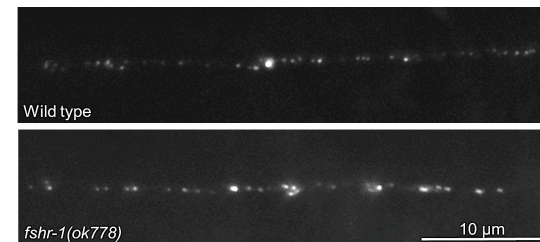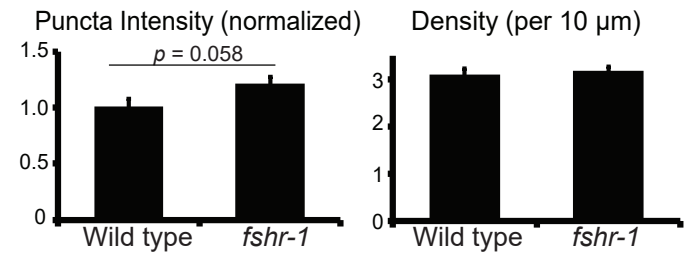

Supplement: S3 Fig — (A) Wild type worms and fshr-1(ok778) mutants that also expressed GFP::SNB-1 in GABAergic (GABA) neurons were imaged using a 100x objective. (Left panel) Representative images of the dorsal nerve cords halfway between the vulva and the tail of young adult animals. (Right panels) Quantification of puncta (synaptic) intensity and puncta density (per 10 μm) ± s.e.m for n = 25 wild type and n = 31 fshr-1. Puncta intensity is shown normalized to wild type. (B-C) Wild type or fshr-1(ok778) mutant animals that also expressed (B) mCherry::UNC-10 or (C) GFP::SYD-2 in GABAergic neurons were imaged using a 100x objective. (Upper panels) Representative images of the dorsal nerve cords halfway between the vulva and the tail of wild type and fshr-1 young adult animals. (Lower panels) Quantification of puncta (synaptic) intensity and puncta density (per 10 μm) ± s.e.m. Puncta intensity is shown normalized to wild type. For (B), n = 26 for wild type, n = 27 for fshr-1. For (C), n = 17 for wild type, n = 20 for fshr-1. Student’s t tests were used to compare the means of the datasets. *p ≤ 0.05 are shown. (PDF) [file pgen.1011461.s003.pdf]

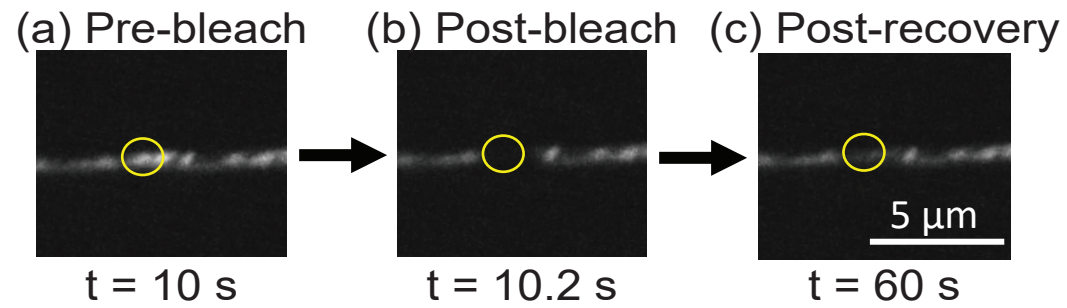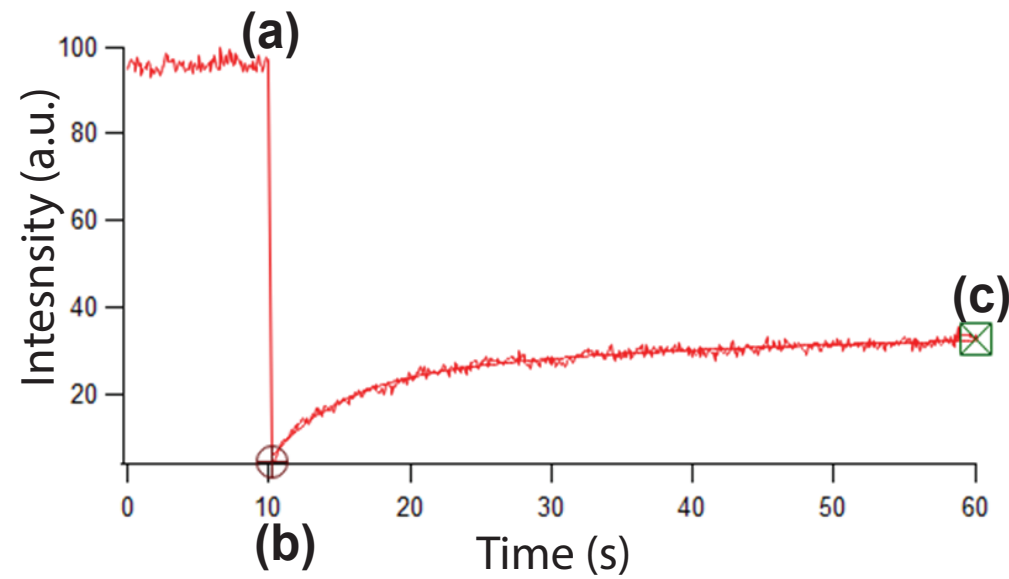

Supplement: S4 Fig — (A) Representative images of pre-bleach(a), post-bleach(b), and post-recovery(c) of SNB-1::SpH labeled vesicles in the dorsal nerve cords in wild type animals expressing SNB-1::SEP in cholinergic motor neurons (Punc-17). Yellow circle marks the ROI of a single SpH punctum. (B) Plot profile of the indicated ROI is shown, indicating the points of measurements of pre-bleach, post-bleach and post-recovery used in calculating % recovery (described in Materials and Methods). (PDF) [file pgen.1011461.s004.pdf]

## Levamisole Paralysis

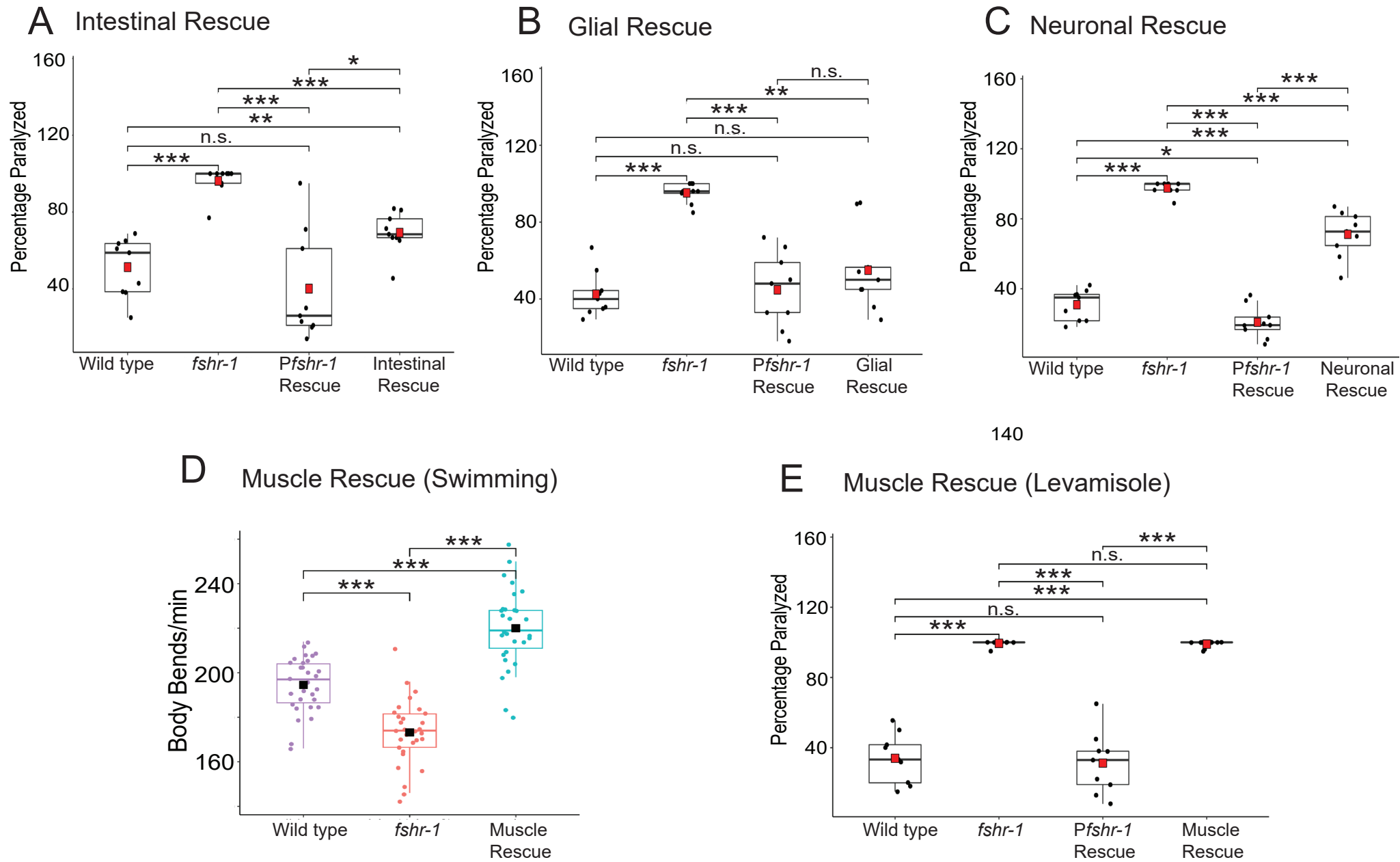

Supplement: S5 Fig — (A-C, E) Box and whisker plots showing results of levamisole paralysis assays performed on wild type and fshr-1(ok778) mutant animals, as well fshr-1 mutants re-expressing fshr-1 (Rescue) in the indicated tissues (A, intestinal Pges-1, ibtEx35; B, glial Pmir-228 ibtEx51; C, neuronal Prab-3 ibtEx34; E, muscle Pmyo-3, kjrEx39). Worms were exposed on plates containing 200μM levamisole for 100 minutes and paralysis was assessed by nose tap. n = 9 plates of approximately 20 young adult animals per plate per strain were tested. (D) Box and whisker plots of swimming experiment data repeated at least twice with fshr-1(ok778) mutants with muscle-specific fshr-1 re-expression. Note that muscle rescue, unlike intestinal, glial, or neuronal rescue, caused increased body bending rates that did not coincide with any restoration of wild type levamisole sensitivity, as seen with the other rescuing transgenes. One-way ANOVA and Tukey’s post hoc tests were used to compare the means of the datasets (*p ≤ 0.05, ** p ≤ 0.01, ***p ≤ 0.001; n.s., not significant). (PDF) [file pgen.1011461.s005.pdf]

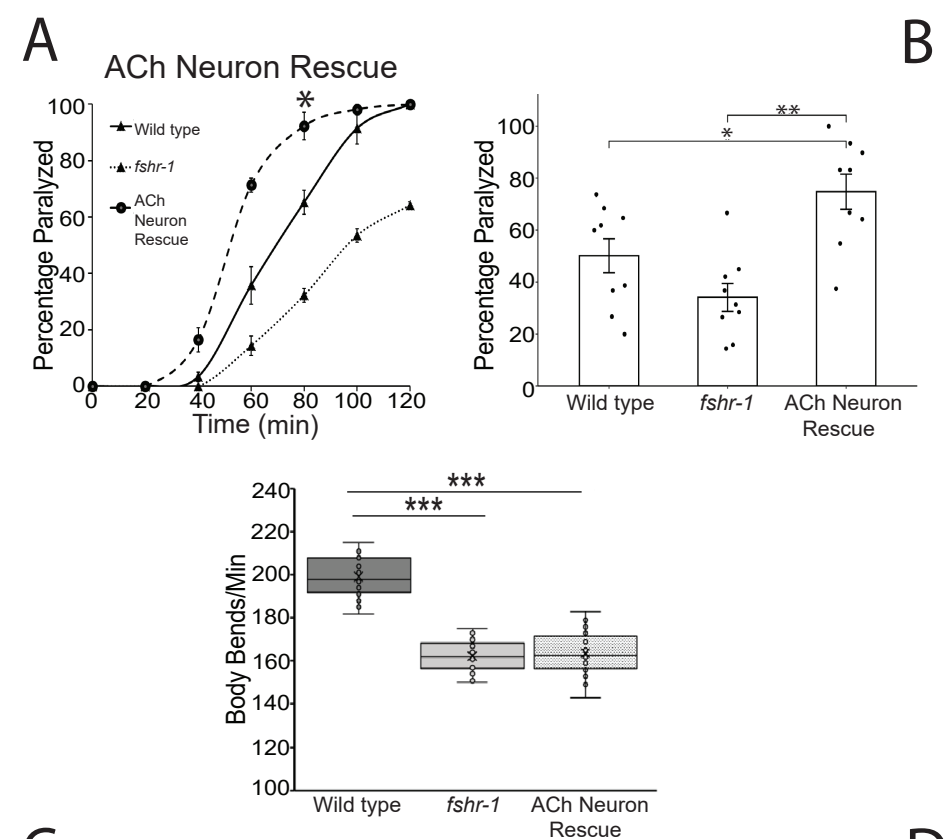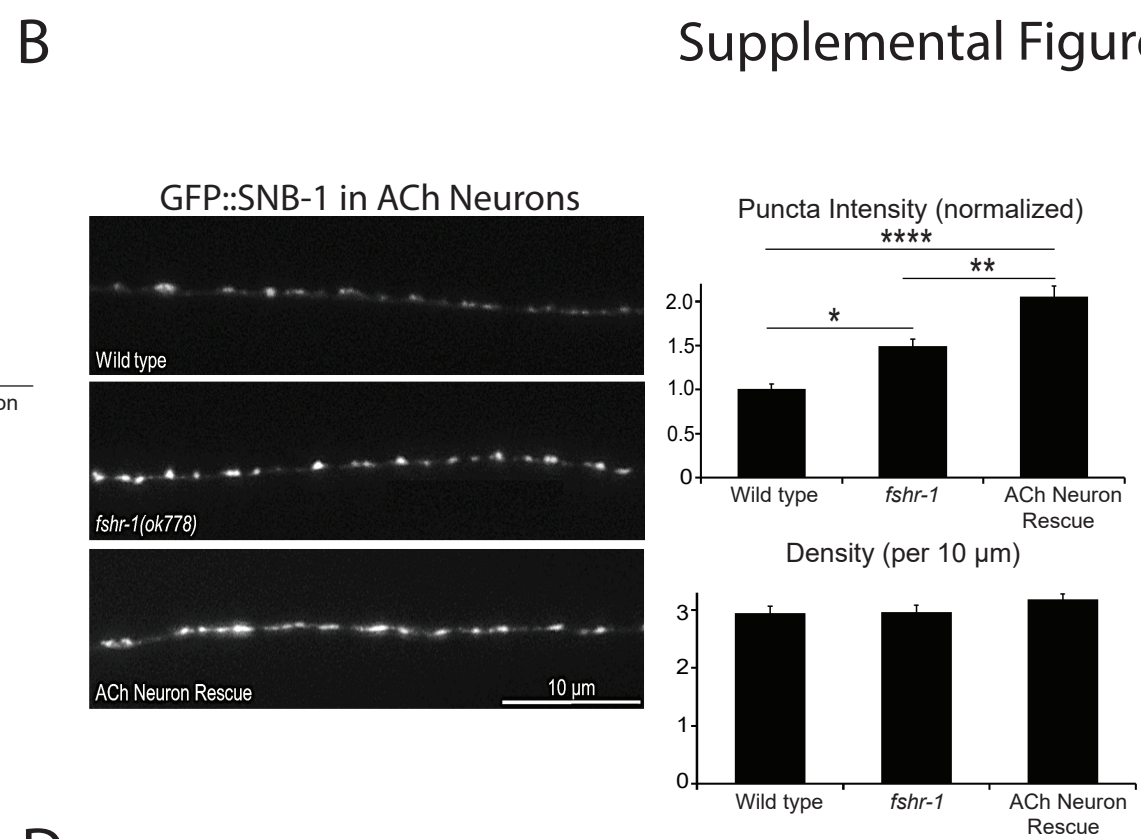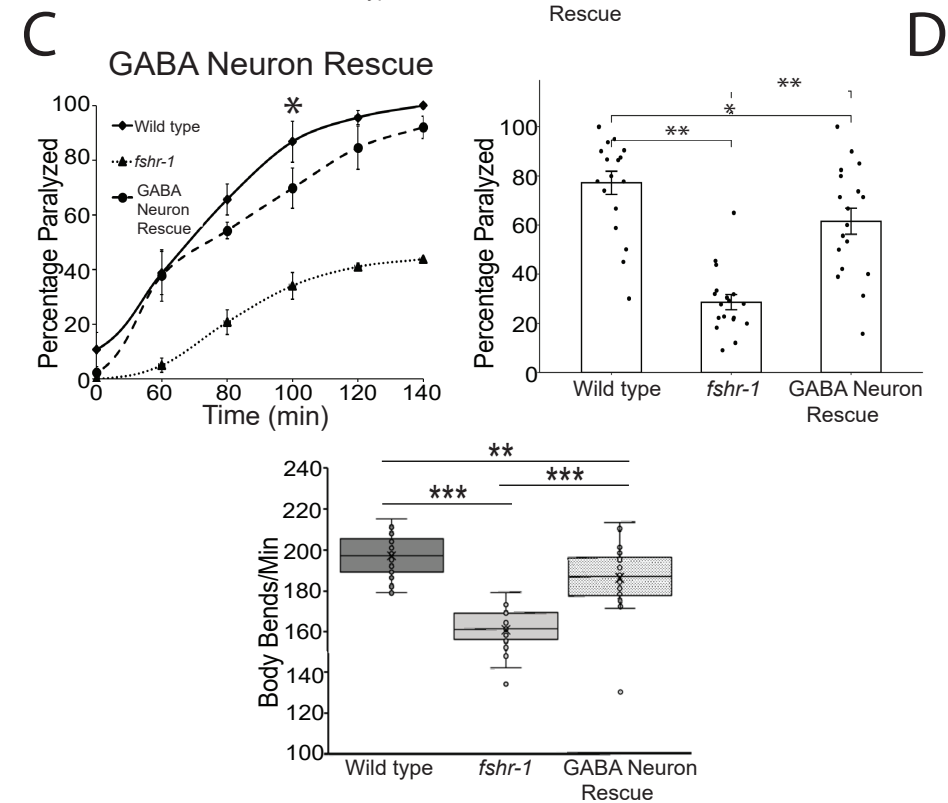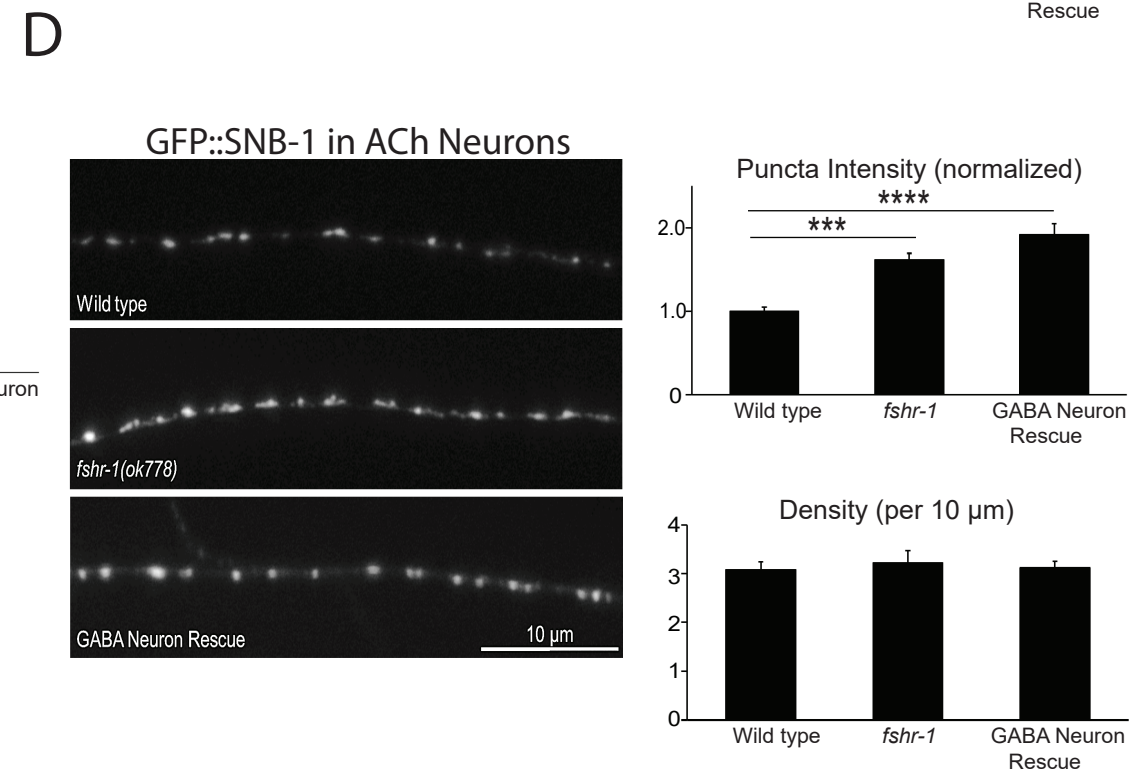

Supplement: S7 Fig — Behavioral (A, C) and synaptic structure (B, D) effects of genomic fshr-1 DNA re-expression in the cholinergic (ACh) neurons (A, B) or GABAergic neurons (C, D) of fshr-1(ok778) mutant animals compared to wild type and fshr-1(ok778) worms. (A, C) (Upper panels) Representative (left, n = 3 plates/strain) and cumulative pooled (right) aldicarb data ± s.e.m. showing complete and even hyper-rescue of aldicarb paralysis in worms with cholinergic neuron-specific fshr-1 rescue (ACh Neuron Rescue) (A) and nearly complete rescue of wild type paralysis in worms with GABAergic neuron-specific rescue (GABA Neuron Rescue) (B). Scatter points show individual plate averages taken at the timepoint indicated by the asterisk (*). (Lower panels) Box and whisker plots showing mean body bends per minute from swimming assays performed on n = 30 young adult animals of each genotype. Minima and maxima (whiskers) are shown, as well as the first and third quartiles of data (boxes), divided by the median line. The “X” denotes the mean value of the data set, and circles show individual data points. Note that while the GABA rescue worms have body bending rates that are partially restored to wild type levels as seen in the swimming assay, ACh rescue worms retain the reduced body bending rates seen with fshr-1 mutants, likely due to the excessive muscle excitation caused by fshr-1 re-expression to above wild type levels (see Upper panels in A vs. C). (B, D) Wild type worms, fshr-1(ok778) mutants, and ACh neuron rescue (B) or GABA neuron rescue (D) animals that also expressed GFP::SNB-1 in cholinergic (ACh) neurons were imaged using a 100x objective. (Left panels) Representative images of the dorsal nerve cords halfway between the vulva and the tail of young adult animals. (Right panels) Quantification of puncta (synaptic) intensity and puncta density (per 10 μm) ± s.e.m. Puncta intensity is shown normalized to wild type. For (B), n = 29 animals imaged for wild type, n = 26 for fshr-1, and n = 32 f [file pgen.1011461.s007.pdf]

Supplemental Figure 8

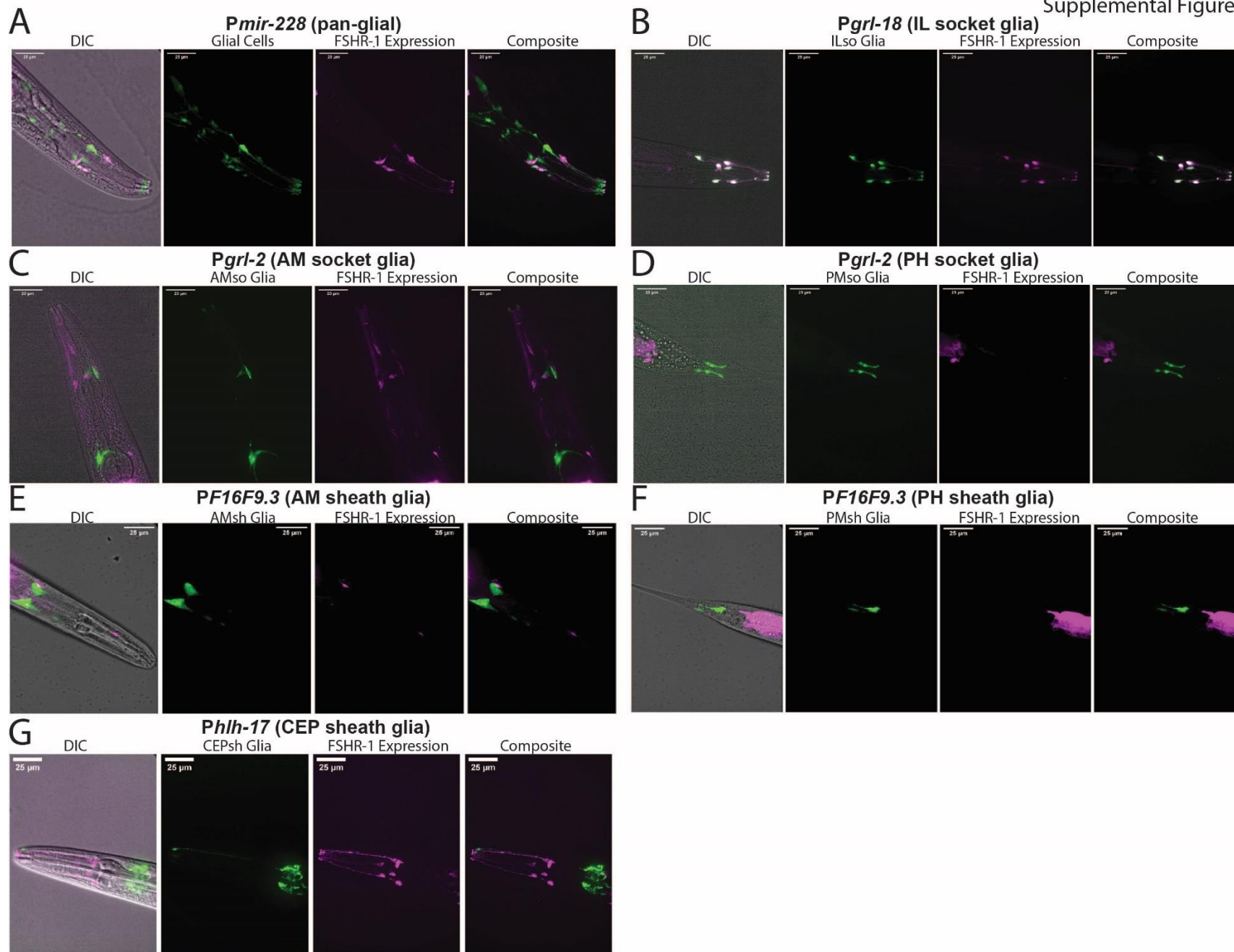

Supplement: S8 Fig — Representative maximum intensity projections of young adult hermaphrodites co-expressing genomic fshr-1 DNA under its own promoter (Pfshr-1, magenta) and markers of various subsets of glial cells (green) imaged in the head and tail regions where glial reside. (A) Pan-glial expression (Pmir-228) shows some colocalization (white, composite) with fshr-1, whereas (B) complete co-localization is seen with fshr-1 and a marker of the six IL socket (ILso) glia (Pgrl-18). No colocalization occurs between fshr-1 and markers of (C, D) AM and PH socket (AMso and PMso) glia (Pgrl-2), (E, F) AM and PH sheath (AMsh and PHsh) glia (PF16F9.3), or (G) CEP sheath (CEPsh) glia. Colocalization was confirmed by matching single planes from the ~25 μm stacks used to create the maximum intensity projects shown here. (PDF) [file pgen.1011461.s008.pdf]
