# Supplementary material for: Cell non-autonomous signaling through the conserved C. elegans glycoprotein hormone receptor FSHR-1 regulates cholinergic neurotransmission
Source: PLoS Genet. 2024 Nov 19;20(11):e1011461. doi: 10.1371/journal.pgen.1011461 (PMC11614273; doi:10.1371/journal.pgen.1011461)

Supplemental Figure 6

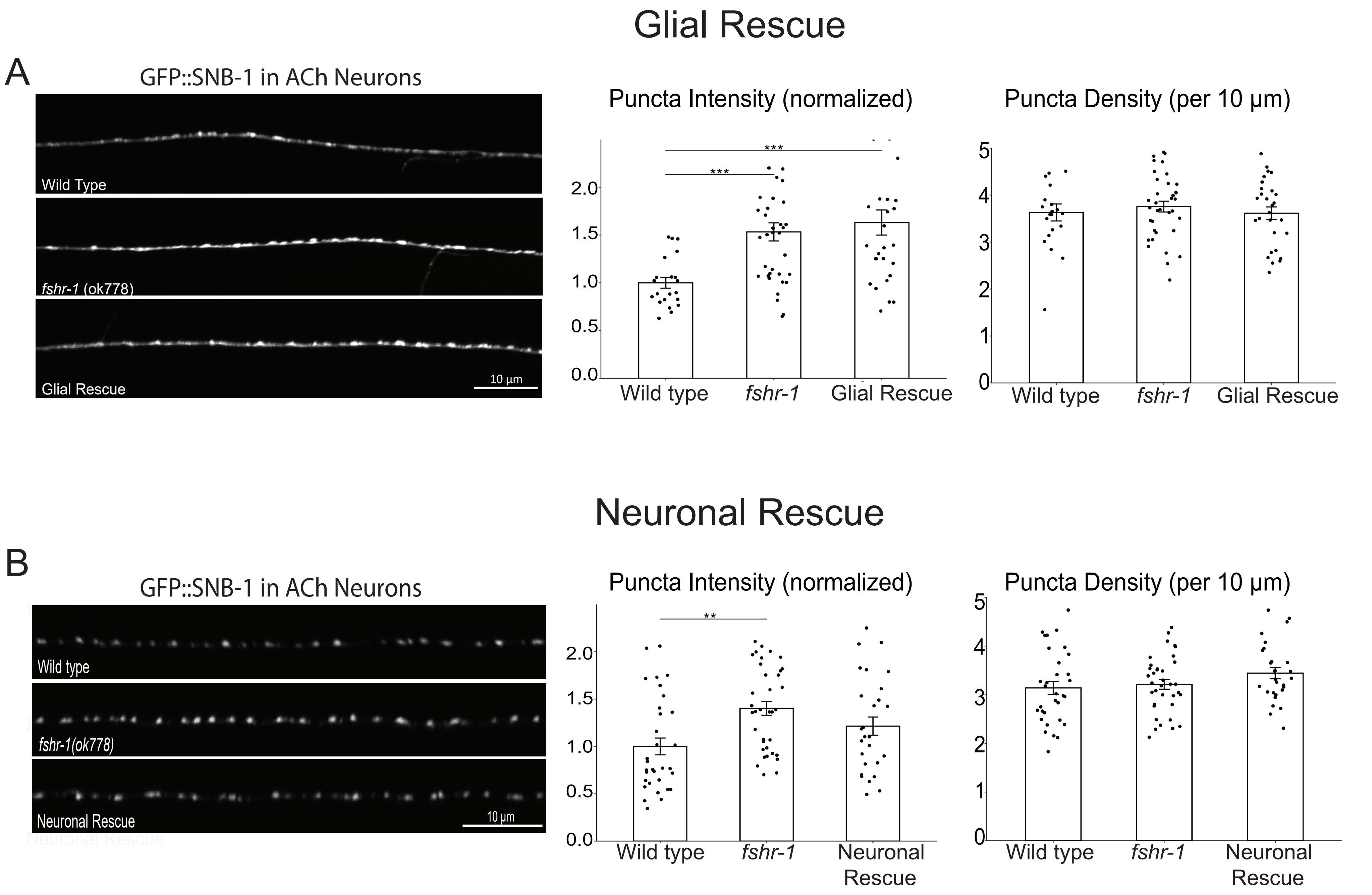

Supplement: S6 Fig — Dorsal nerve cords of wild type worms, fshr-1(ok778) mutants, and animals re-expressing fshr-1 under a pan-glial promoter (A; Pmir-228, ibtEx51) or a pan-neuronal promoter (B; Prab-3, ibtEx34) also expressing GFP::SNB-1 in cholinergic neurons were imaged halfway between the vulva and the tail of young adult animals. (Left panels) Representative images. (Right panels) Quantification of normalized mean puncta (synaptic) intensity and puncta density (per 10 μm) ± s.e.m. Scatter points show individual worm means (n = 21–36 animals per genotype). One-way ANOVA and Tukey’s post hoc tests were used to compare the means of the datasets (*p ≤ 0.05, ** p ≤ 0.01, ***p ≤ 0.001). (PDF) [file pgen.1011461.s006.pdf]
