# Supplementary material for: Cell non-autonomous signaling through the conserved C. elegans glycoprotein hormone receptor FSHR-1 regulates cholinergic neurotransmission
Source: PLoS Genet. 2024 Nov 19;20(11):e1011461. doi: 10.1371/journal.pgen.1011461 (PMC11614273; doi:10.1371/journal.pgen.1011461)
Supplement: S1 Text — (DOCX) [file pgen.1011461.s009.docx]

# **S1 Text. Supplemental Methods and References**

**Supplemental Methods**

*Strains and Strain Maintenance*

Worms were maintained as described in *Materials and Methods.*

| **Strain Number** | **Genotype** | **Reference** |
| --- | --- | --- |
|  | N2 |  |
| JRK165 | *fshr-1(ok778)*6* | *C. elegans* Deletion Mutant Consortium (2012)(1) |
| JRK42 | *nuIs152;fshr-1(ok778)*3* | This study |
| AU209 | *agEx52* P*ric-19::fshr-1(+)*;P*myo-2*::*mCherry*]; *fshr-1*(*ok778*), | Powell et al (2009)(2) |
| JRK56 | *kjrEx15* (P*unc-30::fshr-1*; Pmyo-2::NLS::gfp); *fshr-1(ok778*) | This study |
| WY335 | *fdEx41* [*fshr-1p ::fshr-1 ::gfp*] ; *fshr-1*(*ok778*) | Cho et al (2007)(3) |
| AU218 | *agEx58 [Pges-1::fshr-1;Pmyo-2::NLS::mcherry];fshr-1(ok778)* | Powell et al (2009)(2) |
| AU209 | *agEx52[Pric-19::fshr-1(+);Pmyo-2::mCherry (10ng/ul)];fshr-1(ok778)* | Powell et al (2009)(2) |
| AU251 | *agIs35;fshr-1(ok778) [agIs35 = agEx58 integran*t] | Powell et al (2009)(2) |
| AU175 | *agEx43[Pfshr-1::fshr-1(+);Pmyo-2::mCherry (10ng/ul)];fshr-1(ok778)* | Powell et al (2009)(2) |
| JRK173 | *kjrEx39; [myo-3p::fshr-1cDNA::sl2::mKate 20 ng/µl; unc-122p::gfp 25 ng/µl 10 ng/µl] ; fshr-1*(*ok778*)**6*Line#1 | This study |
| JRK99 | *kjrEx27* (P*unc-17::fshr-1*; P*myo-2::NLS::gfp*), | This study |
| JRK81 | *kjrEx15;nuIs152; fshr-1(ok778)* | This study |
| JRK151 | *kjrEx27;nuIs152;fshr-1(ok778)* | This study |
| JRK65 | *juIs1*2,* | Hallam and Jin (1998)(4) |
| JRK37 | *juIs1;fshr-1(ok778)* | This study |
| JRK170 | *nuIs152; fshr-1(ok778)*; *ibtEx51 [mir228p::fshr-1 cDNA::sl2::mKate 20 ng/µl; unc-122p::GFP 10 ng/µl]* | This study |
| JRK187 | *nuIs152; fshr-1(ok778)*; *ibtEx34 [rab-3p::fshr-1 cDNA::sl2::mKate 20 ng/µl; unc-122p::GFP 25 ng/µl]* | This study |
| ZM2246 | *hpIs88* (*Punc-25::UNC-10::mCherry*), | Hung et al. (2007)(5) |
| JRK106 | *hpIs88;fshr-1,* | This study |
| ZM54 | *hpIs3* | Yeh et al. (2005)(6) |
| JRK58 | *hpIs3;fshr-1(ok778)* | Kowalski et al (2014)(7) |
| IBE89 | *ibtEx15 (Pfshr-1::mCherry); fshr-1 (ok778)* | Kenis et al (2023)(8) |
| OS4260 | *nsIs198 (Pmir-228::gfp)* | Fung et al (2020)(9) |
| JRK176 | *ibtEx15;nIs198;fshr-1(ok778)**6 | This study |
| OS1579 | *nsEx858* (P*F16F9.3::gfp*) | Bacaj et al. (2008)(10) |
| JRK188 | *nsEx858* (P*F16F9.3::gfp*);*fshr-1(ok778)**6 | This study |
| CHB3829 | *hmnIs82 [grl-18p::gfp]* | Cebul et al (2020)(11) |
| JRK178 | *ibtEx15;hmIs82;fshr-1(ok778)**6 | This study |
| CHB3746 | *hmEx2333[grl-2p::CFP; grl-18p ::YFP ; rol-6(su1006)]* | Mizeracka et al (2021)(12) |
| JRK185 | *ibtEx15;hEx2333;fshr-1(ok778)**6 | This study |
| VPR839 | *irIs67 [Phlh-17::gfp + unc-119];unc-119(ed4)* | Stout and Parpura 2011(13) |
| JRK196 | *ibtEx15;irIs67;fshr-1(ok778)*6* | This study |

*Plasmid and Strain Generation*

To create P*unc-17::fshr-1* (pJRK66), the 3.2 kb P*unc-17* promoter was amplified from the PD49.46 backbone in pFJ18 using primers and inserted into the SphI restriction site of pJRK21 upstream of the 5.5 kb genomic *fshr-1* clone. This *fshr-1* DNA was previously amplified from N2 genomic DNA and subcloned into SacI and SpeI sites in PD49.26 (2). To create P*unc-30::fshr-1* (pJRK34), the genomic *fshr-1* DNA was cut out of pJRK21 using SacI and SpeI and subsequently ligated in to the KP1587 plasmid containing the P*unc-30* promoter(14,15). To create P*fshr-1*::*NLS::gfp* (pJRK11), the 4 kb P*fshr-1* promoter(3) was amplified from N2 genomic DNA using primers engineered with SphI and BamHI restriction sites. The amplified promoter was ligated in the PD95.67 vector, which contains *NLS::GFP.*  To create P*myo-3::fshr-1*, the Gateway system was used to generate pIBE219 containing the 2.5 kb *myo-3* promoter (16) and the *fshr-1* cDNA (Kenis et al., 2023).(8)

Transgenic strains were isolated following standard microinjection of the plasmids into the gonads of gravid N2 adult worms as described previously (17). P*unc-17::fshr-1*  (pJRK66) was injected at a concentration of 20 ng/μL, along with 10 ng/μl of Pmyo-2::NLS::gfp (co-injection marker); P*unc-30::fshr-1* (pJRK34) was injected at 50ng/μL along with 10 ng/μl of Pmyo-2::NLS::gfp (co-injection marker); P*myo-3::fshr-1* (pIBE219) was injected at 20ng/μL along with 10 ng/μl of P*unc-122::gfp* (co-injection marker)] into *fshr-1(ok778)*6* animals. Lines were selected and propagated by picking fluorescent animals.

*Single Worm Tracking*

Single worm tracking was carried out using Worm Tracker 2 (18). Individual staged 2-day adult animals were tracked for 5 minutes on Bacto-agar NGM agar plates seeded with a thin lawn of OP50 *E. coli* (50 µl). Movement features were extracted from 5 min of continuous locomotion tracking. Worm tracker software version 2.0.3.1, created by Eviatar Yemini and Tadas Jucikas (Schafer lab, MRC, Cambridge, UK), was used to analyze movement (19).

*NMJ Behavioral Experiments*

Aldicarb and swimming assays (S1 Fig, S7 Fig) were performed as described in Materials and Methods. Levamisole assays (S5 Fig) were performed as follows: NGM agar plates containing 200 μM levamisole (Sigma-Aldrich # L9756) and seeded with 150 µL OP50 *E. coli* were prepared one day prior to the assay. To begin the experiment, 20-25 young adult worms were transferred onto each drug-containing plate. The worms were assayed for complete paralysis after 100 minutes and the average percentage of worms of each strain paralyzed ± S.D. was calculated at each timepoint. Worms were considered paralyzed only if they did not move at all in response to harsh anterior touch with a platinum wire. Three plates were assayed for each strain per experiment with the experimenter always double-blinded to genotype. Experiments were performed at least three times (n = 9 plates).

*Quantitative Imaging*

Widefield imaging (S3 Fig, S7 Fig) and confocal imaging (S6 Fig) were performed as described in the *Materials and Methods*.

*Co-localization Imaging*

Co-localization imaging (S8 Fig) was completed using Nikon Yokogawa Spinning Disk Field Scanning Confocal Microscope equipped with Nikon Elements software. Young adult worms were immobilized in a 30 mg/ml solution of 2,3-butanedione monoxime (BDM) in M9 on a No. 1.5 coverslip (VWR #48366-227) and mounted onto a glass slide containing a 2% agarose pad. Worms were located and marked using a 10x EC Plan-Neofluar 10x/0.30 NA objective and then imaged using a 60x Plan-Apochromat (1.2 NA) water objective. Worms were viewed under FITC/DAPI and TRITC filters to observe the green/blue and red fluorescence respectively. The FITC filter was set to an exposure time of 50ms, a Fast scan, no binning, 16-bit image with the 488nm laser at 26.9%. The DAPI filter was set to an exposure time of 100ms, a Fast scan, no binning, 16-bit image with the 405nm laser at 26.9%. The TRITC filter was set to a 600ms exposure time, an Ultra-quiet scan, no binning, 16-bit image with the 561nm laser at 26.9%. The top and bottom of the stack were defined as the positions in which the fluorescence from the FITC/DAPI filter went out of focus, giving a total stack size of ~20-25 μm with 0.5 μm step sizes. For imaging, the FITC/DAPI and TRITC filters were set to be used at every stack, with the DIC filter taking one image in the middle (home) plane. Images of 30-35 worms were acquired per strain to ensure the full panel of expression was detected given the use of an extrachromosomal array, which has inherently variable expression levels, for these experiments. Merged images were generated both for maximum intensity projections and for individual image planes. Individual image planes from each stack were examined under both the FITC/DAPI and TRITC filters, finding points where red fluorescence in the TRITC filter overlapped in the same plane as green/blue fluorescence under the FITC/DAPI filter. These spots of overlap, especially if in the same shape, represent fluorescence in the same cell and therefore, some level of colocalization. These spots were identified and counted for all images taken to determine the maximum number of cells per strain where *fshr-1* expression was observed. Representative maximum intensity projections of images showing the maximal number of colocalized cells are shown. Prior to imaging the dual reporter strains, bleed-through imaging was performed similarly on both single reporter strains for every imaging pair to ensure co-localization was not the result of bleed-through.

**References**

1. C. elegans Deletion Mutant Consortium. large-scale screening for targeted knockouts in the *Caenorhabditis elegans* genome. *G3 (Bethesda)*. 2012 Nov;2(11):1415–25.

2. Powell JR, Kim DH, Ausubel FM. The G protein-coupled receptor FSHR-1 is required for the *Caenorhabditis elegans* innate immune response. *PNAS*. 2009 Feb 24;106(8):2782–7.

3. Cho S, Rogers KW, Fay DS. The C. elegans Glycopeptide Hormone Receptor Ortholog, FSHR-1, Regulates Germline Differentiation and Survival. *Current Biology*. 2007 Feb;17(3):203–12.

4. Hallam SJ, Jin Y. lin-14 regulates the timing of synaptic remodelling in *Caenorhabditis elegans*. *Nature*. 1998 Sep 3;395(6697):78–82.

5. Hung W, Hwang C, Po MD, Zhen M. Neuronal polarity is regulated by a direct interaction between a scaffolding protein, Neurabin, and a presynaptic SAD-1 kinase in *Caenorhabditis elegans*. *Development*. 2007 Jan;134(2):237–49.

6. Yeh E, Kawano T, Weimer RM, Bessereau JL, Zhen M. Identification of Genes Involved in Synaptogenesis Using a Fluorescent Active Zone Marker in *Caenorhabditis elegans*. J Neurosci. 2005 Apr 13;25(15):3833–41.

7. Kowalski JR, Dube H, Touroutine D, Rush KM, Goodwin PR, Carozza M, et al. The Anaphase-Promoting Complex (APC) ubiquitin ligase regulates GABA transmission at the *C. elegans* neuromuscular junction. *Mol Cell Neurosci*. 2014 Jan;58:62–75.

8. Kenis S, Istiban MN, Van Damme S, Vandewyer E, Watteyne J, Schoofs L, et al. Ancestral glycoprotein hormone-receptor pathway controls growth in *C. elegans*. *Front Endocrinol (Lausanne*). 2023;14:1200407.

9. Fung W, Wexler L, Heiman MG. Cell-type-specific promoters for *C. elegans* glia. *Journal of Neurogenetics.* 2020 Oct 1;34(3–4):335–46.

10. Bacaj T, Tevlin M, Lu Y, Shaham S. Glia are essential for sensory organ function in *C. elegans*. *Science*. 2008 Oct 31;322(5902):744–7.

11. Cebul ER, McLachlan IG, Heiman MG. Dendrites with specialized glial attachments develop by retrograde extension using SAX-7 and GRDN-1. *Development.* 2020 Feb 17;147(4):dev180448.

12. Mizeracka K, Rogers JM, Rumley JD, Shaham S, Bulyk ML, Murray JI, et al. Lineage-specific control of convergent differentiation by a Forkhead repressor. *Development.* 2021 Oct 1;148(19):dev199493.

13. Stout RF, Parpura V. Voltage-gated calcium channel types in cultured *C. elegans* CEPsh glial cells. *Cell Calcium.* 2011 Jul;50(1):98–108.

14. Jin Y, Hoskins R, Horvitz HR. Control of type-D GABAergic neuron differentiation by *C. elegans* UNC-30 homeodomain protein. *Nature*. 1994 Dec 22;372(6508):780–3.

15. Vashlishan AB, Madison JM, Dybbs M, Bai J, Sieburth D, Ch’ng Q, et al. An RNAi screen identifies genes that regulate GABA synapses. *Neuron.* 2008 May 8;58(3):346–61.

16. Choi U, Hu M, Zhang Q, Sieburth D. The head mesodermal cell couples FMRFamide neuropeptide signaling with rhythmic muscle contraction in *C. elegans.* *Nat Commun.* 2023 Jul 14;14(1):4218.

17. Mello CC, Kramer JM, Stinchcomb D, Ambros V. Efficient gene transfer in *C.elegans*: extrachromosomal maintenance and integration of transforming sequences. *EMBO J.* 1991 Dec;10(12):3959–70.

18. Yemini E, Kerr RA, Schafer WR. Preparation of Samples for Single-Worm Tracking. *Cold Spring Harb Protoc.* 2011 Dec;2011(12):pdb.prot066993.

19. Yemini E, Jucikas T, Grundy LJ, Brown AEX, Schafer WR. A database of *Caenorhabditis elegans* behavioral phenotypes. *Nat Methods.* 2013 Sep;10(9):877–9.
