# Supplementary material for: Cell non-autonomous signaling through the conserved C. elegans glycoprotein hormone receptor FSHR-1 regulates cholinergic neurotransmission
Source: PLoS Genet. 2024 Nov 19;20(11):e1011461. doi: 10.1371/journal.pgen.1011461 (PMC11614273; doi:10.1371/journal.pgen.1011461)
Supplement: S6 Data — (DOCX) [file pgen.1011461.s015.docx]

# **Quantitative Imaging Igor Analysis Summary Data**

# **Figure 2A - nuIs152 (GFP::SNB-1) in ACh Motor Neurons with Pfshr-1 Rescue**

## ***Summary Table***


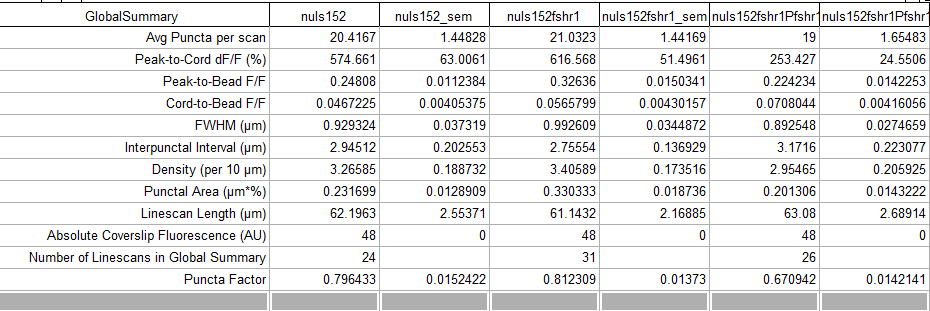


## ***Puncta Intensity***

- COMPARE: nuIs152_Peakworm to nuIs152fshr1_Peakworm
- Tukey Kramer P value is 0.0013768
- COMPARE: nuIs152_Peakworm to nuIs152fshr1Pfshr1_Peakworm
- Tukey Kramer P value is 0.60717
- COMPARE: nuIs152fshr1_Peakworm to nuIs152fshr1Pfshr1_Peakworm
- Tukey Kramer P value is 2.7497e-05

## ***Puncta Density***

- COMPARE: nuIs152_Densityworm to nuIs152fshr1_Densityworm
- Tukey Kramer P value is 0.85961
- COMPARE: nuIs152_Densityworm to nuIs152fshr1Pfshr1_Densityworm
- Tukey Kramer P value is 0.50505
- COMPARE: nuIs152fshr1_Densityworm to nuIs152fshr1Pfshr1_Densityworm
- Tukey Kramer P value is 0.20134

# **Figure 2C - nuIs165 (GFP::UNC-10) in ACh Motor Neurons**

***Summary Table***


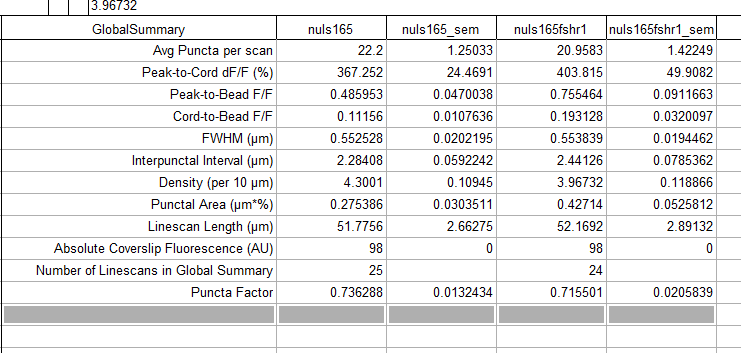


## ***Puncta Intensity***

- COMPARE: nuIs165_Peakworm to nuIs165fshr1_Peakworm
- Student's T Test gives a p value of 0.021356.

## ***Puncta Density***

- COMPARE: nuIs165_Densityworm to nuIs165fshr1_Densityworm
- Student's T Test gives a p value of 0.045069.

# **Figure 2D - nuIs159 (GFP::SYD-2) in ACh Motor Neurons**

## ***Summary Table***


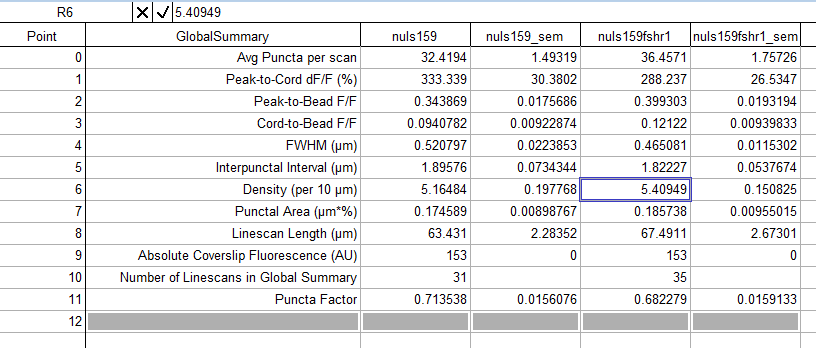


## ***Puncta Intensity***

- COMPARE: nuIs159_Peakworm to nuIs159fshr1_Peakworm
- Student's T Test gives a p value of 0.056709

## ***Puncta Density***

- COMPARE: nuIs159_Densityworm to nuIs159fshr1_Densityworm
- Student's T Test gives a p value of 0.32939.

# **Figure 2E - wyIs687 (GFP::CLA-1) in ACh Motor Neurons**

## ***Summary Table***


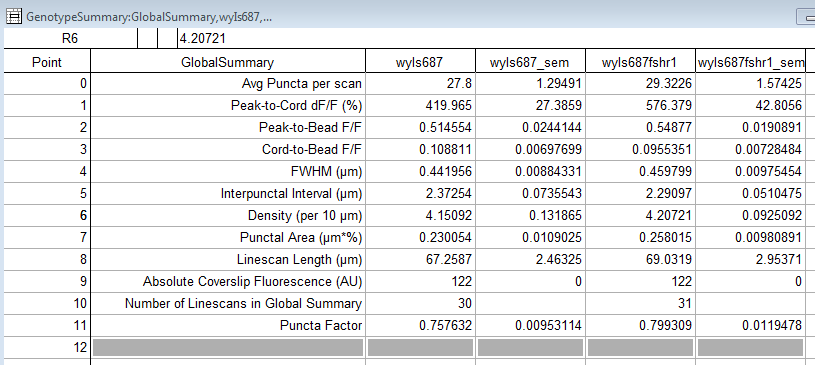


## ***Puncta Intensity***

- COMPARE: wyIs687_Peakworm to wyIs687fshr1_Peakworm
- Student's T Test gives a p value of 0.45565.

## ***Puncta Density***

- COMPARE: wyIs687_Densityworm to wyIs687fshr1_Densityworm
- Student's T Test gives a p value of 0.72814.

# **Figure 2F - nuIs195 (INS-22::VENUS) in ACh Motor Neurons**

## ***Summary Table***


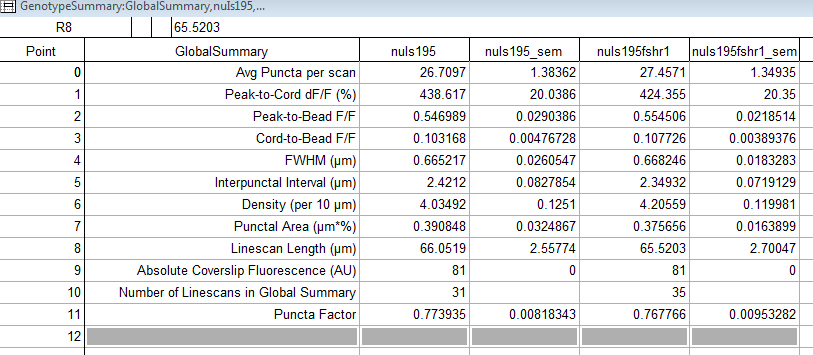


## ***Puncta Intensity***

- COMPARE: nuIs195_Peakworm to nuIs195fshr1_Peakworm
- Student's T Test gives a p value of 0.98886.

## ***Puncta Density***

- COMPARE: nuIs195_Peakworm to nuIs195fshr1_Peakworm
- Student's T Test gives a p value of 0.98886.

# **Supplemental Figure 2A - juIs1 (GFP::SNB-1) in GABA Neurons**

## ***Summary Table***


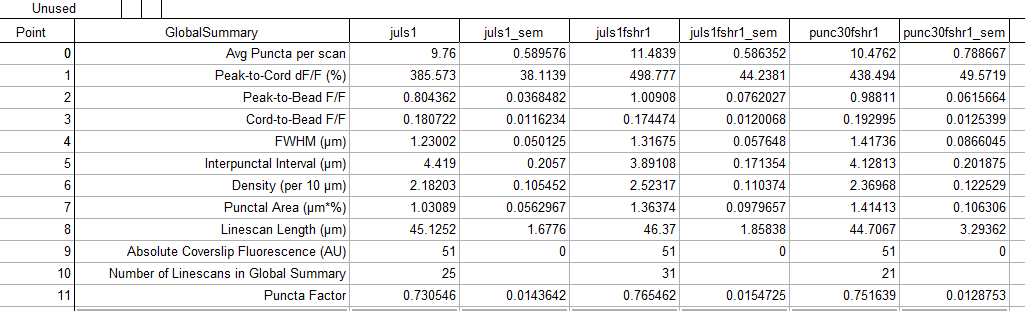


## ***Puncta Intensity***

- COMPARE: juIs1_Peakworm to juIs1fshr1_Peakworm
- Student's T Test gives a p value of 0.029619.

## ***Puncta Density***

- COMPARE: juIs1_Densityworm to juIs1fshr1_Densityworm
- Student's T Test gives a p value of 0.029612.

# **Supplemental Figure 2B – hpIs88 (mCherry::UNC-10) in GABA Neurons**

## ***Summary Table***


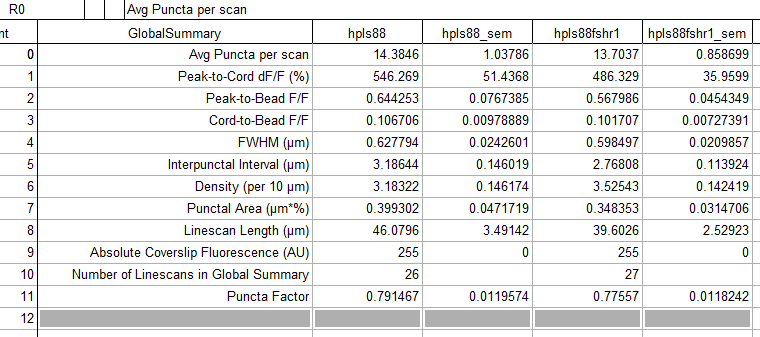


## ***Puncta Intensity***

- COMPARE: hpIs88_Peakworm to hpIs88fshr1_Peakworm
- Student's T Test gives a p value of 0.39373.

## ***Puncta Density***

- COMPARE: hpIs88_Densityworm to hpIs88fshr1_Densityworm
- Student's T Test gives a p value of 0.099714.

# **Supplemental Figure 2C - hpIs3 (GFP::SYD-2) in GABA Neurons**

## ***Summary Table***


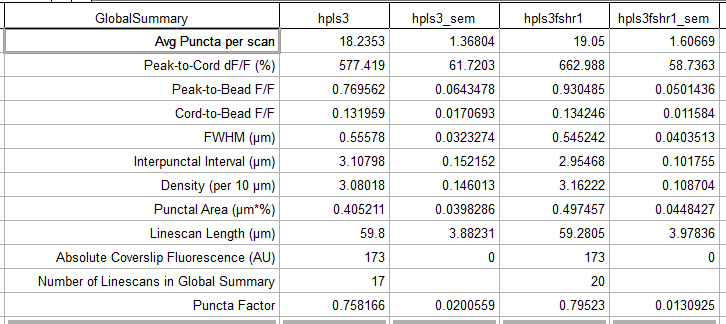


## ***Puncta Intensity***

- COMPARE: hpIs3_Peakworm to hpIs3fshr1_Peakworm
- Student's T Test gives a p value of 0.057716.

## ***Puncta Density***

- COMPARE: hpIs3_Densityworm to hpIs3fshr1_Densityworm
- Student's T Test gives a p value of 0.6554.

# **Supplemental Figure 7B - nu152 (GFP::SNB-1) in ACh Neurons with FSHR-1 Cholinergic Rescue**

## ***Summary Table***


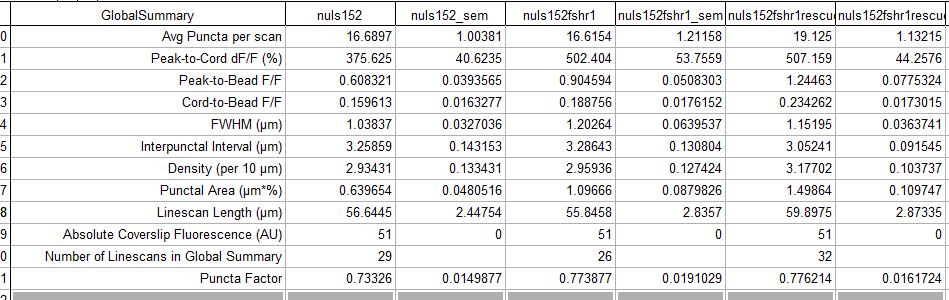


## ***Puncta Intensity***

- COMPARE: nuIs152_Peakworm to nuIs152fshr1_Peakworm
- Tukey Kramer P value is 0.030129
- COMPARE: nuIs152_Peakworm to nuIs152fshr1rescue_Peakworm
- Tukey Kramer P value is 1.7542e-08
- COMPARE: nuIs152fshr1_Peakworm to nuIs152fshr1rescue_Peakworm
- Tukey Kramer P value is 0.0012421

## ***Puncta Density***

- COMPARE: nuIs152_Densityworm to nuIs152fshr1_Densityworm
- Tukey Kramer P value is 0.9889
- COMPARE: nuIs152fshr1_Densityworm to nuIs152fshr1rescue_Densityworm
- Tukey Kramer P value is 0.41899
- COMPARE: nuIs152_Densityworm to nuIs152fshr1rescue_Densityworm
- Tukey Kramer P value is 0.31909

# **Supplemental Figure 7C - nu152 (GFP::SNB-1) in ACh Neurons with FSHR-1 GABAergic Rescue**

## ***Summary Table***


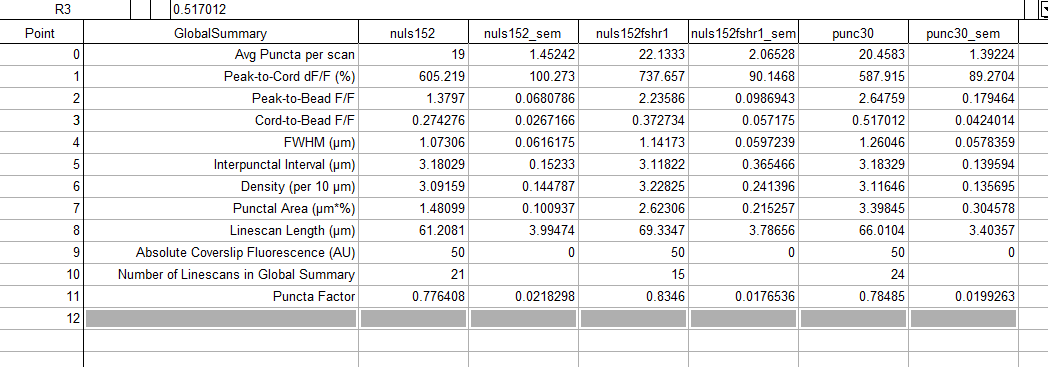


## ***Puncta Intensity***

- COMPARE: nuIs152_Peakworm to nuIs152fshr1_Peakworm
- Tukey Kramer P value is 0.00043708
- COMPARE: nuIs152fshr1_Peakworm to punc30_Peakworm
- Tukey Kramer P value is 0.32291
- COMPARE: nuIs152_Peakworm to punc30_Peakworm
- Tukey Kramer P value is 2.109e-07

## ***Puncta Density***

- COMPARE: nuIs152_Densityworm to nuIs152fshr1_Densityworm
- Tukey Kramer P value is 0.84876
- COMPARE: nuIs152fshr1_Densityworm to punc30_Densityworm
- Tukey Kramer P value is 0.89058
- nuIs152_Densityworm to punc30_Densityworm
- Tukey Kramer P value is 0.99305
