# Supplementary material for: Cell non-autonomous signaling through the conserved C. elegans glycoprotein hormone receptor FSHR-1 regulates cholinergic neurotransmission
Source: PLoS Genet. 2024 Nov 19;20(11):e1011461. doi: 10.1371/journal.pgen.1011461 (PMC11614273; doi:10.1371/journal.pgen.1011461)
Supplement: Attachment — Submitted filename: 144278_1_rebuttal_2999177_sc7bgd.pdf [file pgen.1011461.s020.pdf]

Revision Plan

Review COMMONS

Manuscript number: RC-2024-02385
Corresponding author(s): Jennifer R. Kowalski

[The "revision plan" should delineate the revisions that authors intend to carry out in response to the points raised by the referees. It also provides the authors with the opportunity to explain their view of the paper and of the referee reports.

The document is important for the editors of affiliate journals when they make a first decision on the transferred manuscript. It will also be useful to readers of the reprint and help them to obtain a balanced view of the paper.

If you wish to submit a full revision, please use our "Full Revision" template. ***It is important to use the appropriate template to clearly inform the editors of your intentions.****]*

1. General Statements [optional]

This section is optional. Insert here any general statements you wish to make about the goal of the study or about the reviews.

Our manuscript describes a novel role for the conserved glycoprotein hormone receptor, FSHR-1, in regulating *C. elegans* neuromuscular function through an inter-tissue, gut-brain signaling pathway. FSHR-1 is the sole *C. elegans* homolog of a family of vertebrate glycoprotein receptors that includes FSHR, TSHR, and LHR, and has previously been shown to regulate body size, germline differentiation, lipid homeostasis, and various stress responses in the worm (Kenis at al 2023; Cho et al 2007; Torzone et al 2023; Powell et al 2009; Miller et al 2015; Robinson and Powell 2016; Wei and Kowalski, 2018; Kim and Sieburth 2020; Wang et al 2023) but its role in neuromuscular regulation, although identified in a 2005 RNA interference screen (Sieburth et al 2005), has not been previously explored. Here, through a combination of genetic, behavioral, and fluorescence imaging approaches, we demonstrate that FSHR-1 is both necessary and sufficient in the intestine of the worm (and may also act in several other distal tissues, including glia and head neurons) to promote muscle excitation through effects on active zone protein localization and synaptic vesicle release from cholinergic motor neurons. Additionally, we identify the FSHR-1 ligands, glycoproteins GPLA-1 and GPLB-1, as well as several known downstream effectors of FSHR-1 in other contexts, GSA-1/GalphaS, ACY-1/adenylyl cyclase, and the lipid kinase SPHK-1, as interactors in the FSHR-1 pathway for neuromuscular control. This work represents a detailed description of the ability of this conserved and multi-functional receptor in inter-tissue coordination that may ultimately be connected to its functions in other physiological processes, such as the stress response, and may also prove relevant for understanding roles for FSHR-1 homologs in humans.

Revision Plan

We greatly appreciate the thoughtful and constructive feedback provided by each of the three reviewers of this manuscript. We are pleased that all three reviewers noted the novelty of the mechanisms of cross-tissue regulation of neuromuscular function by FSHR-1 that we uncovered. Reviewer 1 comments, "They demonstrate a novel phenomenon of cross-tissue regulation by restoring FSHR-1 in neurons, intestines, or glia to restore NMJ function." Reviewer 2 echoes this sentiment, also noting, "*The data is well presented, compelling and the conclusions are well supported by the data*. . .. *[T]his study provides a solid foundation to address many interesting questions regarding the role of fshr-1 signaling in regulating neuronal function."* Reviewer 3 adds "*This is a highly worthy contribution to the field of cell non-autonomous signaling and neuromodulation, and specifically synaptic transmission modulation. The study deepens and enhances the understanding of fshr-1 function within the C. elegans intestine and adds in several molecular components into the signaling pathway, acting both upstream and downstream. . . While this work relies on an invertebrate system of C. elegans, all components have vertebrate counterparts, so findings are likely of broader interest."*

As described below, we are working to address many of the comments made by the reviewers and have already made some of the suggested minor changes to the manuscript. We are hopeful that, given the reviewers' excitement about this work, the changes we have already made, and the additional revisions we intend to make in the coming months, including the completion of several new experiments we propose in the revision plan below, our manuscript will be of interest to a broad genetics audience.

2. Description of the planned revisions

Insert here a point-by-point reply that explains what revisions, additional experimentations and analyses are planned to address the points raised by the referees.

Planned Revisions based on comments from Reviewer #1

- **The authors found that expressing FSHR-1 in intestinal cells was sufficient to compensate for the fshr-1 mutation phenotype, suggesting that intestinal cell FSHR-1 can regulate neuromuscular junction (NMJ) function across tissues. However, the molecular mechanism remains unexplored. Since the downstream signaling pathways of FSHR-1 are clear, analyzing the gain-of-function (gf) mutations of gsa-1 and acy-1 in different tissues can help elucidate the signaling pathways transmitted across tissues.**

We completely agree that tissue-specific pathway analysis is important for understanding the molecular mechanism underlying the ability of FSHR-1 to control neuromuscular function from its location in distal tissues, like the intestine. Because of the complexity of these questions and the time required for us to generate strains to perform tissue-specific protein depletion or overexpression experiments, we intend these studies to be the focus of a future manuscript However, in lieu of performing a full suite of tissue-specific analyses of FSHR-1 downstream components, we will perform intestine-specific RNA interference experiments (as we did for *fshr-1* in Figure 4B) of *gsa-1, acy-1, and sphk-1* in wild type worms and in animals

overexpressing *fshr-1* in the intestine (which causes increased swimming behavior, Figure 3A) to determine if these downstream players are required for the effects of intestinal *fshr-1* on the NMJ. We appreciate the reviewer's suggestion to address these important questions regarding the site of action of the downstream players.

- **The images of neurons should be presented in higher resolution and magnification to provide clearer visualization.**

We appreciate the reviewer's request for increased visualization of the neurons; however, because the current larger, lower resolution images show several release sites and were used for the quantitative analyses we present, we would like to keep the images as they are. However, *we will provide higher resolution insets for the images in Figures 2A, 2C-F, and 4C, as requested.*

- **It is unclear whether the glycoprotein subunit orthologs act in the intestine to regulate NMJ function with FSHR-1. This should be investigated and clarified in the manuscript.**

We fully agree that determining where and how the glycoproteins GPLA-1 and GPLB-1 interact with FSHR-1 – and if this is happening at the level of the intestine - is an important outstanding question. Based on prior work, it is known that these subunits are not expressed in intestinal cells, but they are found in several gut-associated neurons and tissues. Specifically, *gpla-1* is expressed in neurons of the gastrointestinal tract, including M1, M5, I5 and NSM pharyngeal motor neurons, as well the AVL and DVB excitatory motor neurons that control defecation contractions in the hindgut. *gplb-1* is also expressed in the DVB neuron, as well as in non-neuronal tissues (head mesodermal cells and the hindgut enteric muscles), and both glycoprotein genes show reporter expression in the RME motor neurons in the head (Kenis et al 2023). *We will complete experiments testing whether the effects of intestinal FSHR-1 overexpression require the ligands, as suggested by Reviewer #2.* We intend that our future work will explore the glycoprotein-FSHR-1 interactions more deeply in a variety of contexts.

- **In Figure 4C, there are no error bars, and individual values should be shown in all statistical analyses to provide a complete representation of the data and its variability.**

We again thank the reviewer for catching this error in Figure 4C. We have replaced the graph with the complete one that includes error bars. *We will replace the graphs in 1B, 1C, 3D, 4A, 4C, 5A, 5B, and 6E, as well as Supplemental Figure 5A, 5B, 6A, 6B, 7A, and 7C with bars overlaid with the individual data points.* We are unable to do this for Figures 2A-F or Supplemental Figures 2A-C, 7B or 7D because these analyses were run using Custom-written Igor software (Burbea et al 2002) that does not provide individual values, only mean values and cumulative probability plots of the datasets. We recently showed consistency between the Igor analysis program and the newer Fiji plug-in we used for our more recent imaging data, supporting concordance of results despite not having the individual data points in Igor (Hulsey-Vincent et al 2023).

Revision Plan

Planned Revisions based on comments from Reviewer #2

- **Fig 4B: An intestinal site of action seems likely for fshr-1 and is nicely supported by the intestine-specific RNAi experiment in Fig 4B. Does intestine-specific knockdown of fshr-1 also cause the aldicarb and SNB-1 defects seen in the mutant? Including this data especially for the synaptic markers would strengthen the gut to neuron inter-tissue signaling model that is proposed here (OPTIONAL).**

We appreciate the reviewer's suggestion to include additional intestine-specific knockdown data for the aldicarb, SNB-1::GFP, and other imaging data. *We have the reagents to perform the intestine-specific knockdown of fshr-1 in the aldicarb assay and will complete these experiments as part of our revision plan.* Although performing the same experiments in the imaging strains requires first crossing each imaging line to the intestine-specific RNAi line, which may may prove challenging, *we are currently working to cross the intestinal RNAi line with nuIs152, the cholinergic SNB-1::GFP line and, assuming the cross goes well, will include results in our revised manuscript.*

- **Fig 5A: The authors show that G alpha s and adenylyl cyclase function downstream of fshr-1, but it is unclear whether these are direct fshr-1 effectors or whether they function less directly. Does expressing gsa-1(gf) or acy-1(gf) transgenes specifically in the intestine (or neurons) suppress the fshr-1 defects? (OPTIONAL)**

As stated in our response to Reviewer #1, we completely agree that tissue-specific pathway analysis is important for understanding the molecular mechanism underlying the ability of FSHR-1 to control neuromuscular function from its location in distal tissues, like the intestine. While the complexity of these questions and the time required for us to generate strains to perform tissue-specific protein depletion or overexpression experiments is likely more than is suitable for the revision time frame of this manuscript (and will be the focus of future work), in lieu of these experiments *we will perform intestine-specific RNA interference experiments (as we did for fshr-1 in Figure 4B) of gsa-1, acy-1, and sphk-1 in wild type worms and in animals overexpressing fshr-1 in the intestine (which causes increased swimming behavior, Figure 3A) to determine if these downstream players are required for the effects of intestinal fshr-1 on the NMJ.* We appreciate the reviewer's suggestion to address these important questions regarding the site of action of the downstream players.

- **Fig 6A-D: The authors propose that fshr-1 is activated by its ligands for locomotion, but no evidence is presented to support this. This could be experimentally addressed with the reagents that are used in this study by determining whether the increased locomotion caused by overexpressing fshr-1 in the intestine (reported in Fig 3A), is dependent upon gpla-1 and/or gplb-1 activity. This experiment would help to distinguish whether gpla-1 and/or gplb-1 indeed are fshr-1 ligands or whether fshr-1 functions in a ligand-independent manner, and would justify the sentence on line 526 "...ligands...act upstream in this context..."**

We agree with the reviewer that the question of GP ligand activation of FSHR-1 in this context is an important and interesting question. We plan to cross the intestinal *fshr-1* transgene into the *gpla-1, gplb-1,* and *gpla-1gplb-1* mutants, as suggested and then will test their swimming

behavior to see if the overexpression effect depends upon the ligands. We thank the reviewer for this experimental suggestion.

Planned Revisions based on comments from Reviewer #3

- **Within Figure 6, the authors state that an experiment was run 2-3X which seems inconsistent with other figure panels. It would be better if three times was consistently used. Adding in another run seems appropriate. To add another experimental run where needed within Figure 6 A-D seems realistic. The strains, reagents and skills are all in place, so the only significant investment is time. These experiments should be able to be completed in a few weeks/months.**

We appreciate the reviewer's desire for consistency in terms of the number of replicates. We will ensure all swimming experiments, which were the experiments in question in Figure 6, have been completed at least 3 times as part of our revision plan.

- **The authors findings would be strengthened by doing further work to delineate in which tissues the downstream factors act, by doing tissue specific epistasis basically for gsa-1, acy-1 etc. This would entail a lot of work and would delay publication significantly. I do not see this as necessary unless the authors wish for a big impact journal publication.**

As stated in our response to Reviewers #1 and 2, we agree that tissue-specific pathway analysis is important for understanding the molecular mechanism underlying the ability of FSHR-1 to control neuromuscular function from its location in distal tissues, like the intestine. While the complexity of these questions and the time required for us to generate strains to perform tissue-specific protein depletion or overexpression experiments is likely more than is suitable for the revision time frame of this manuscript (and will be the focus of future work), in lieu of these experiments *we will perform intestine-specific RNA interference experiments (as we did for fshr-1 in Figure 4B) of gsa-1, acy-1, and sphk-1 in wild type worms and in animals overexpressing fshr-1 in the intestine (which causes increased swimming behavior, Figure 3A) to determine if these downstream players are required for the effects of intestinal fshr-1 on the NMJ.* We appreciate the reviewer's suggestion to address these important questions regarding the site of action of the downstream players.

- ***Figures:* Overall the authors have presented everything in a clear and thorough manner. Some modification of the Y-axes on several aldicarb resistance graphs & body bend bar graphs could improve the clarity. Trying to standardize the Y axis range and the tick mark locations would make it easier to read and compare between figures and panels.**

We appreciate the reviewer's attention to detail here and will work to further standardize the Y-axes on the graphs as requested.

3. Description of the revisions that have already been incorporated in the transferred manuscript

Please insert a point-by-point reply describing the revisions that were already carried out and included in the transferred manuscript. If no revisions have been carried out yet, please leave this section empty.

Revisions made to the manuscript in response to comments by Reviewer #1

- **The authors should demonstrate the expression of FSHR-1 in various tissues, as this is essential for analyzing its function.**

We appreciate the reviewer's request for additional clarity regarding the sites of tissue-specific FSHR-1 expression and agree that this information was not sufficiently clear in the text. It is already known that FSHR-1 is expressed in various tissues (e.g., head neurons, glia, intestine) from prior studies (Cho et al, 2007; Kenis et al 2023; Hammarlund et al 2018); thus, we would like to defer to Reviewer #3's suggestion about the expression information and have added a description of FSHR-1 expression patterns to lines 129 -130 within the *Introduction* of the paper. (Reviewer #3: "*In the discussion there is a section about the reported areas of endogenous fshr-1 expression. I would have appreciated knowing that information much earlier in the paper. Without being reminded of the reported normal expression pattern it is difficult to fully appreciate why how the neuronal and glial expression could be at work*") This expression information is also mentioned in the *Results* section lines 420-421 when we first discuss the tissue-specific rescue experiments.

- **Figure 4A appears to be the same as Figure S5B. The authors should ensure that the figures are correctly labeled and distinct from each other.**

We thank the reviewer for noticing this oversight. We apologize for the inadvertent duplication. We have replaced the graphs in Figure 4A with the correct rescue experiment using the P*ges-1*, *ibtEx35*-expressing strain.

Revisions made to the manuscript in response to comments by Reviewer #2

- **Fig 3: Using transgenic rescue experiments the authors observe rescue when expressing fshr-1 under promoters for the intestine as well as glia and neurons. Is it possible that the apparent rescue using glia and neuronal promoters may arise from leaky expression of these transgenes in the intestine? Leaky intestinal expression is a reported caveat for rescue experiments. This possibility should be discussed**.

We appreciate the reviewer's note regarding the potential caveat of leaky intestinal expression. We have added a mention of this possibility to the discussion (lines 612-616) where we outline other potential explanations for the ability of multiple transgenes to rescue the neuromuscular phenotype. This possibility is why we feel most confident in the intestine site of action given that we have intestine-specific RNA interference data showing *fshr-1* necessity in this tissue. We also acknowledge the need for tissue-specific depletion studies to address requirements for *fshr-1* in the other distal tissues. We hope to be able to address these other potential sites of action in our future work.

- **Fig 4B: Please clarify at what stage the intestine-specific knockdown of fshr-1 was conducted. It would be informative to treat animals with fshr-1 RNAi at various developmental stages to distinguish whether fshr-1 plays a developmental or post-developmental role in this process (OPTIONAL).**

We thank the reviewer for bringing to our attention the omission of details regarding the feeding RNA interference experiments. We have added an "RNA Interference" subsection with this information to the *Materials and Methods* section of the manuscript. Briefly, the intestine-specific knockdown was performed by feeding worms at the L4 stage HT115(DE) bacteria containing L4440 empty plasmid or one targeting *fshr-1*. Worms were grown for 4 days on NGM agar plates containing Ampicillin and IPTG, then offspring of the treated worms were assayed at the young adult stage. Thus, the knockdown animals we tested had been exposed to the RNAi for their lifetime. We are very interested in exploring the developmental timing of *fshr-1* expression and function in future work; thus, we thank the reviewer for this suggestion. However, we feel that a detailed panel of developmental knockdown effects of *fshr-1* is beyond the scope of the current study.

- ***Fig 4C: Is rescue significant? p values are not shown.***

In figure 4C, *p*-values are only shown for statistically significant differences, as noted in the figure legend. A Tukey's post-hoc test indicates that the Intestinal Rescue strain is not significantly different from either the wild type *or* the *fshr-1* mutants, indicating partial rescue. While we cannot fully explain the discrepancy between the partial rescue of the SNB-1::GFP phenotype in light of the full behavioral rescue in the swimming, aldicarb, and crawling assays, we suspect it may be due to the fact that synaptic vesicle release has been sufficiently restored to recover neuromuscular signaling even though synaptic vesicle localization is not fully returned to wild type levels, given the variable and likely non-endogenous levels of *fshr-1* re-expression from the tissue-specific transgenes. We have noted this discrepancy in the Discussion (lines 633-639) when considering the levamisole and SNB-1::GFP data in light of the aldicarb and swimming results. *"For some tissue-specific fshr-1 expression experiments, we observed partial rescue of the swimming and crawling fshr-1 mutant phenotypes without a restoration of normal synaptic vesicle localization (e.g., cholinergic motor neurons, GABAergic motor neurons, glial cells, Supplemental Figures 6 and 7). We conclude that GFP::SNB-1 accumulation may not solely report on rates of synaptic vesicle release and/or that there are compensatory mechanisms for increasing muscle excitation (e.g. upregulation of postsynaptic ACh receptors or muscle excitatory machinery."*

- **Fig 6E. There are two bars in this graph labeled gpla-1; gplb-1 that show significantly different amplitudes. Please clarify and define the different colors that each graph is outlined with.**

We thank the reviewer for catching this error. The third bar from the left should say "*gpla-1;fshr-1*". We have corrected this in the manuscript. We have also added descriptions of the colors to the figure legend indicating the following: dark blue = wild type, yellow = *fshr-1*; green = glycopeptide mutants; blue = glycopeptide;*fshr-1* mutants. Similar clarification has been added to the legend for the bar graph in Figure 3D.

Revision Plan

Revisions made to the manuscript in response to comments by Reviewer #3

Suggested Text Revisions: I have some suggestions to consider.

- **In the abstract the term expression analysis is used to analyses of areas of FSHR-1 function using tissue specific rescue experiments. Expression analysis often means directly exploring mRNA, localization, or levels using transcriptomic approaches or reporter genes so some revision of language could improve accuracy in the abstract.**

We appreciate the reviewer's point and have removed the phrase "expression analysis" from the summary at the end of the *Introduction* section where it initially appeared.

- **In Figure 1, the authors do not comment on the overexpression phenotype or why this strain was included.**

We thank the reviewer for noticing this oversight. We have added a sentence describing the overexpression experiment and its implications in our description of Figure 1 in the *Results* section (lines 337-339).

- **In the discussion there is a section about the reported areas of endogenous fshr-1 expression. I would have appreciated knowing that information much earlier in the paper. Without being reminded of the reported normal expression pattern it is difficult to fully appreciate why how the neuronal and glial expression could be at work.**

We appreciate the reviewer's request for additional clarity regarding the sites of tissue-specific FSHR-1 expression and agree that this information was not sufficiently clear in the text prior to the discussion. We have added a description of FSHR-1 expression patterns to lines 129 -130 within the *Introduction* of the paper. It is also mentioned in *Results* section lines 420-421 when we first discuss the tissue-specific rescue experiments.

- **The section on tissue specific rescue could be written more strongly. The use of many "transition" phrases dilutes the importance of the findings in this paragraph.**

We are grateful for the reviewer's suggestions to improve the clarity of the text, specifically regarding the tissue-specific rescue section. We have tightened up the text in this section of the *Discussion* (lines 547-621) to remove some of the transitional phrases. We believe this has enhanced the readability of the manuscript and the impact of our findings.

- ***Figures:* Fig. 3 panel D: it is not clear what the last 2 bars (Neuronal rescues) are being compared to, its it w.t.? Were the differences between fshr-1 and these rescues not significantly different?**

We appreciate the reviewer bringing this point of confusion to our attention with Figure 3D. We have clarified in the figure legend that the Neuronal rescue bars are compared to wild type and that there is no significant difference from the *fshr-1* mutants for these two lines, further supporting our central focus on the intestine as the best-supported site of FSHR-1 action.

4. Description of analyses that authors prefer not to carry out

Please include a point-by-point response explaining why some of the requested data or additional analyses might not be necessary or cannot be provided within the scope of a revision. This can be due to time or resource limitations or in case of disagreement about the necessity of such additional data given the scope of the study. Please leave empty if not applicable.

Comment from Reviewer #1

- **The article concludes that the fshr-1 mutation affects the release of acetylcholine vesicles. However, using fluorescent proteins to label key proteins released by vesicles may introduce artifacts. Therefore, electron microscopy should be used to analyze vesicle accumulation for more reliable results.**

We thank the reviewers for this suggestion and acknowledge the potential value of EM to definitively show vesicle accumulation in *fshr-1* mutants. However, these experiments are technically demanding, involve specialized high-pressure freezing, and would require us to establish new collaborations to complete; thus, we would not be able to be complete such experiments in a timeframe reasonable for revision. While the fluorescence microscopy experiments admittedly offer less resolution, this approach has been used with great success in numerous other studies to identify alterations in synaptic vesicle localization in motor neurons that correlate with electron microscopy, electrophysiology, and aldicarb data that more directly measure numbers of synaptic vesicles and synaptic function (Jorgensen et al 1995; Jin et al 1999; Nonet et al 1999). Thus, we believe that the pHluorin experiments, coupled with the SNB-1::GFP imaging, are sufficient to demonstrate defects in vesicle release, regardless of the specific effects on vesicle clustering. We have been mindful not to overstate our conclusions (lines 371-372: "*Together, these data demonstrate that FSHR-1 signaling promotes the localization and/or release of cholinergic synaptic vesicles*.") We hope the reviewer will agree that our analysis provides meaningful information about SV organization in the absence of EM level experiments.

- **The authors analyzed the release of vesicles from GABA and acetylcholine (Ach) neurons separately to demonstrate that the fshr-1 mutation specifically affects Ach neuron vesicle release. However, while GFP::SNB-1 and GFP::SYD-1 accumulated in GABA neurons, mCherry::UNC-10 did not change significantly in GABA neurons. To fully understand vesicle release, the authors should also use synaptopHluroin (SpH) to analyze GABA neuron vesicle release.**

We agree that our data indicate that, in addition to effects on cholinergic synaptic vesicle release, there may be effects on release of vesicles from GABAergic neurons, and we acknowledge this in the manuscript. However, while we are interested in potentially exploring the effects of *fshr-1* in GABAergic neurons, we believe this question requires extensive additional work that is beyond the scope of this manuscript, which is focused on *fshr-1* effects on cholinergic signaling. Moreover, given that *fshr-1*-deficient animas are aldicarb resistant (Figure 1A), it is unlikely that GABA release is decreased. If GABA release was decreased, we would expect hypersensitivity to aldicarb. Thus, while it is still possible there are different effects

on GABA vesicles, our data suggest the most physiological relevant effect is on cholinergic signaling. We do acknowledge in the *Discussion* that it will be of interest to determine the relevance of effects in the GABA neurons (lines 649-651).
