# Supplementary material for: Cell non-autonomous signaling through the conserved C. elegans glycoprotein hormone receptor FSHR-1 regulates cholinergic neurotransmission
Source: PLoS Genet. 2024 Nov 19;20(11):e1011461. doi: 10.1371/journal.pgen.1011461 (PMC11614273; doi:10.1371/journal.pgen.1011461)
Supplement: Attachment — Submitted filename: PGENETICS-D-24-00438_List of Changes to the Manuscript.docx [file pgen.1011461.s021.docx]

**List of Changes to the Manuscript and Completion of Proposed Revision Plan**

In response to reviewer comments from our initial submission to *Review Commons,* we submitted a revision plan along with a preliminary revised manuscript to *PLoS Genetics.* We have completed all the proposed experiments, as well as text and figure revisions included in that plan. A summary of the results of the new experiments performed, as well as a list of the changes to figures and text, that we have made to incorporate those new data, as well as to address the other revisions proposed, are included below.

***Revision Plan* Experiments Completed**

1. As suggested by Reviewers #1, 2, and 3, we performed intestine-specific RNA interference (RNAi) experiments knocking down expression of *gsa-1, acy-1, and sphk-1* in wild type worms and in animals overexpressing *fshr-1* in the intestine (which causes increased swimming behavior, Figure 3A) to determine if these downstream players are required for the effects of intestinal *fshr-1* on the NMJ.
   a. **Result:** Knocking down expression of each of the FSHR-1 effectors only in the intestine caused minimal effects on swimming behavior when depleted on their own; however, knock down of each of these three genes was reduced the increased swimming rates caused by intestinal *fshr-1* overexpression (Figure 7A-C). These results demonstrate that *gsa-1, acy-1*, and *sphk-1* are required for the ability of intestinal fshr-1 to drive increased neuromuscular function, particularly under conditions of heightened FSHR-1 activation or expression, and support a cell autonomous role for these molecules downstream of intestinal FSHR-1.
2. As suggested by Reviewer #2, we completed experiments testing whether the effects of intestinal FSHR-1 overexpression require the ligands.
   a. **Result:** We found that animals overexpressing *fshr-1* in the intestine but carrying deletions in *gpla-1* and/or *gplb-1* were unable to promote the increased swimming rates seen with intestinal *fshr-1* overexpression alone (Figure 7D-F). These data indicate that despite some evidence of constitutive receptor activity in other systems, the activity of the FSHR-1 receptor in the *C. elegan*s intestine requires the presence of the GPLA-1 and GPLB-1 ligands.
3. As suggested by Reviewer #2, we performed intestine-specific knockdown of *fshr-1* in the aldicarb assay, as well as crossed the intestinal RNAi line with *nuIs152*, the cholinergic SNB-1::GFP line and performed quantitative imaging experiments on these animals.
   a. **Result:** Unfortunately, the aldicarb experiments proved inconclusive and highly variable, such that it was not possible to obtain clear results. Interpretation of the aldicarb experiments was complicated by the dominant *rol-6* mutation in the RNAi sensitized strain, which disrupts movement and which we think may have impacted feeding on the aldicarb and detection of paralysis.
   b. **Result:** In the SNB-1::GFP imaging experiments, while there was a trend towards increased synaptic vesicle accumulation with intestine-specific knockdown of *fshr-1*(see Reviewer Figure 1 below), consistent with the *fshr-1* genetic *lf* animals (Fig 2A), the results were more variable day to day and there was no statistically significant trend. The intestinal *fshr-1* RNAi animals also showed trends towards decreased puncta density, which could be due to multiple brighter puncta appearing as a single punctum in the analysis. While these RNAi data are consistent with our genetic mutant data, we felt that they, like the aldicarb data, were too variable to warrant inclusion in the manuscript.

**Reviewer Figure 1.**  Intestine-specific RNAi knockdown of *fshr-1* shows trends towards increased SNB-1::GFP puncta intensity and decreased SNB-1::GFP puncta density. (n = 5 experimental days, 6-12 animals imaged per day per treatment; control = L4440 empty plasmid; gfp = *gfp* knockdown (both negative controls, as *gfp* knockdown in the intestine should not impact SNB-1::GFP in the motor neurons).

**Proposed *Revision Plan* Text and Figure Changes to the Manuscript Completed**

- (*Reviewer #1 suggestion*) High resolution insets were added to Figures 2A, C-F and Figure 4C, as requested.
- (*Reviewer #1 suggestion*) We replaced the graphs in 1B, 1C, 3D, 4A, 4C, 5A, 5B, and 6E, as well as Supplemental Figure 5A, 5B, 6A, 6B, 7A, and 7C with bars overlaid with the individual data points. As noted previously in the *Revision Plan,* we are unable to do this for Figures 2A-F or Supplemental Figures 2A-C, 7B or 7D because these analyses were run using Custom-written Igor software (Burbea et al 2002) that does not provide individual values, only mean values and cumulative probability plots of the datasets. We recently showed consistency between the Igor analysis program and the newer Fiji plug-in we used for our more recent imaging data, supporting concordance of results despite not having the individual data points in Igor (Hulsey-Vincent et al 2023).
- (*Review #3 suggestion*) All swimming data were updated to include at least three replicates of all experiments. For experiments done with genetic mutants (Figures 1B, 3A-C, 5A-C, 6A-D, and new 7D-F), graphs now show the average from 3-6 independent experiments in which 30 worms of each genotype were tested; RNAi experiment graphs (Figures 4B, new 7A-C) show the average of 5-12 experiments in which 10 worms of each treatment group were tested. The box and whisker plots previously used for these data were switched to bar graphs with overlaid data points for better data visualization in this format.
- (*Reviewer #3 suggestion*) Y-axes in Figures 1-7 have been standardized as requested to the best of our abilities.

**Additional Text and/or Figure Changes to the Manuscript**

- Figure 1A was replaced with new data using the *ibtEx15* *Pfshr-1* rescue strain to be consistent with the strain used for the rest of the manuscript. The original Figure 1A aldicarb experiment graphs with the *fdEx41 Pfshr-1* rescue strain were moved to Supplemental Figure 5 with the other secondary rescue strains used as confirmation. We also simplified Figure 1B by removing the lefthand graph of the *fdEx41* strain and moved it to Supplemental Figure 5 for the same reason.
- All worm tracking experiments (Figures 1C, 3D, and 6E) now also show the average of 3-10 experiments in which 10 worms/strain were tested per genotype. Bar graphs are now overlaid with individual data points as requested.
- A new Figure 7 was added with data showing the effects of Intestinal FSHR-1 overexpression in combination with either intestinal knockdown of *acy-1,* gsa-1, or *sphk-1* (A-C) or mutation of *gpla-1, gplb-1,* or *gpla-1gplb-1* (D-F).
- The model that was previously Figure 7 is now Figure 8. The model has also been adjusted to remove question marks from GSA-1, ACY-1, and SPHK-1 in the intestine since our data do support a role for these three proteins there.
- An additional panel (G) was added to Supplemental Figure 8, as we completed additional imaging demonstrating lack of localization of our *fshr-1* transcription reporter to CEP sheath glia marked by the P*hlh-17* reporter.
- Text was added at lines 375-394 in the Results section and at lines 563-566 in the Discussion section to describe new data in Figure 7.
- Information about strains that had been inadvertently left out of the strain list in Table 1 was added.
- *Materials and Methods* section was moved to follow the *Discussion* as per the standard *PLOS Genetics* research article format.
- Funding information was removed from the *Acknowledgements* per *PLOS Genetics* instructions.
- References format was switched to Vancouver (ICMJE) per *PLOS Genetics* instructions.
