# Supplementary material for: Cell non-autonomous signaling through the conserved C. elegans glycoprotein hormone receptor FSHR-1 regulates cholinergic neurotransmission
Source: PLoS Genet. 2024 Nov 19;20(11):e1011461. doi: 10.1371/journal.pgen.1011461 (PMC11614273; doi:10.1371/journal.pgen.1011461)
Supplement: Peer review history

# Author comment: Author response to previous submission

**DOI:** 10.1371/journal.pgen.1011461.r001

22 Apr 2024

Attachment Submitted filename: 144278_1_rebuttal_2999177_sc7bgd.pdf

# Editor report: Decision Letter 0

**DOI:** 10.1371/journal.pgen.1011461.r002

**Authors:** Giovanni Bosco

27 Apr 2024

Dear Dr Kowalski,

Thank you very much for submitting your Research Article entitled 'Cell non-autonomous signaling through the conserved C. elegans glycoprotein hormone receptor FSHR-1 regulates cholinergic neurotransmission' to PLOS Genetics.

The manuscript was fully evaluated at the editorial level, and independent peer reviewer comments (provided through Review Commons) were considered. The reviewers appreciated the attention to an important problem, but raised some substantial concerns about the original manuscript. We were pleased to read your detailed revision plan-- this was very useful. Based on the reviews, and your robust revision plan submitted along with your manuscript, we would like to invite you to submit a much revised manuscript that incorporates additional changes you detailed in your revision plan. We are confident that this resubmission would greatly improve your manuscript, and then we will consider it again for publication. We cannot, of course, promise publication at that time, however if we are satisfied that your revised manuscript adheres to your detailed revision plan, then another round of reviews may not be necessary.

Should you decide to revise the manuscript for further consideration here, your revisions should address the specific points made by each reviewer and adhere very closely to the revision plan you submitted. We will also require a detailed list of the changes you have made in the manuscript.

If you decide to revise the manuscript for further consideration at PLOS Genetics, please aim to resubmit within the next 60 days, unless it will take extra time to address the concerns of the reviewers, in which case we would appreciate an expected resubmission date by email to plosgenetics@plos.org.

If present, accompanying reviewer attachments are included with this email; please notify the journal office if any appear to be missing. They will also be available for download from the link below. You can use this link to log into the system when you are ready to submit a revised version, having first consulted our Submission Checklist.

To enhance the reproducibility of your results, we recommend that you deposit your laboratory protocols in protocols.io, where a protocol can be assigned its own identifier (DOI) such that it can be cited independently in the future. Additionally, PLOS ONE offers an option to publish peer-reviewed clinical study protocols. Read more information on sharing protocols at https://plos.org/protocols?utm_medium=editorial-email&utm_source=authorletters&utm_campaign=protocols

Please be aware that our data availability policy requires that all numerical data underlying graphs or summary statistics are included with the submission, and you will need to provide this upon resubmission if not already present. In addition, we do not permit the inclusion of phrases such as "data not shown" or "unpublished results" in manuscripts. All points should be backed up by data provided with the submission.

While revising your submission, please upload your figure files to the Preflight Analysis and Conversion Engine (PACE) digital diagnostic tool.  PACE helps ensure that figures meet PLOS requirements. To use PACE, you must first register as a user. Then, login and navigate to the UPLOAD tab, where you will find detailed instructions on how to use the tool. If you encounter any issues or have any questions when using PACE, please email us at figures@plos.org.

PLOS has incorporated Similarity Check, powered by iThenticate, into its journal-wide submission system in order to screen submitted content for originality before publication. Each PLOS journal undertakes screening on a proportion of submitted articles. You will be contacted if needed following the screening process.

To resubmit, use the link below and 'Revise Submission' in the 'Submissions Needing Revision' folder.

We are sorry that we cannot be more positive about your manuscript at this stage. Please do not hesitate to contact us if you have any concerns or questions.

Yours sincerely,

Giovanni Bosco, Ph.D.

Section Editor

PLOS Genetics

Giovanni Bosco

Section Editor

PLOS Genetics

# Author comment: Author response to Decision Letter 0

**DOI:** 10.1371/journal.pgen.1011461.r003

20 Sep 2024

Attachment Submitted filename: PGENETICS-D-24-00438_List of Changes to the Manuscript.docx

# Editor report: Decision Letter 1

**DOI:** 10.1371/journal.pgen.1011461.r004

**Authors:** Anne C. Hart, Giovanni Bosco

14 Oct 2024

Dear Dr Kowalski,

We are pleased to inform you that your manuscript entitled "Cell non-autonomous signaling through the conserved C. elegans glycoprotein hormone receptor FSHR-1 regulates cholinergic neurotransmission" has been editorially accepted for publication in PLOS Genetics. Congratulations!

Before your submission can be formally accepted and sent to production you will need to complete our formatting changes, which you will receive in a follow up email. Please be aware that it may take several days for you to receive this email; during this time no action is required by you. Please note: the accept date on your published article will reflect the date of this provisional acceptance, but your manuscript will not be scheduled for publication until the required changes have been made.

Once your paper is formally accepted, an uncorrected proof of your manuscript will be published online ahead of the final version, unless you’ve already opted out via the online submission form. If, for any reason, you do not want an earlier version of your manuscript published online or are unsure if you have already indicated as such, please let the journal staff know immediately at plosgenetics@plos.org.

In the meantime, please log into Editorial Manager at https://www.editorialmanager.com/pgenetics/, click the "Update My Information" link at the top of the page, and update your user information to ensure an efficient production and billing process. Note that PLOS requires an ORCID iD for all corresponding authors. Therefore, please ensure that you have an ORCID iD and that it is validated in Editorial Manager. To do this, go to ‘Update my Information’ (in the upper left-hand corner of the main menu), and click on the Fetch/Validate link next to the ORCID field.  This will take you to the ORCID site and allow you to create a new iD or authenticate a pre-existing iD in Editorial Manager.

If you have a press-related query, or would like to know about making your underlying data available (as you will be aware, this is required for publication), please see the end of this email. If your institution or institutions have a press office, please notify them about your upcoming article at this point, to enable them to help maximise its impact. Inform journal staff as soon as possible if you are preparing a press release for your article and need a publication date.

Thank you again for supporting open-access publishing; we are looking forward to publishing your work in PLOS Genetics!

Yours sincerely,

Anne C. Hart

Academic Editor

PLOS Genetics

Giovanni Bosco

Section Editor

PLOS Genetics

www.plosgenetics.org

Twitter: @PLOSGenetics

----------------------------------------------------

Comments from the reviewers (if applicable):

----------------------------------------------------

**Data Deposition**

If you have submitted a Research Article or Front Matter that has associated data that are not suitable for deposition in a subject-specific public repository (such as GenBank or ArrayExpress), one way to make that data available is to deposit it in the Dryad Digital Repository. As you may recall, we ask all authors to agree to make data available; this is one way to achieve that. A full list of recommended repositories can be found on our website.

The following link will take you to the Dryad record for your article, so you won't have to re‐enter its bibliographic information, and can upload your files directly: 

http://datadryad.org/submit?journalID=pgenetics&manu=PGENETICS-D-24-00438R1

More information about depositing data in Dryad is available at http://www.datadryad.org/depositing. If you experience any difficulties in submitting your data, please contact help@datadryad.org for support.

Additionally, please be aware that our data availability policy requires that all numerical data underlying display items are included with the submission, and you will need to provide this before we can formally accept your manuscript, if not already present.

----------------------------------------------------

**Press Queries**

If you or your institution will be preparing press materials for this manuscript, or if you need to know your paper's publication date for media purposes, please inform the journal staff as soon as possible so that your submission can be scheduled accordingly. Your manuscript will remain under a strict press embargo until the publication date and time. This means an early version of your manuscript will not be published ahead of your final version. PLOS Genetics may also choose to issue a press release for your article. If there's anything the journal should know or you'd like more information, please get in touch via plosgenetics@plos.org.

# Editor report: Acceptance letter

**DOI:** 10.1371/journal.pgen.1011461.r005

**Authors:** Anne C. Hart, Giovanni Bosco

28 Oct 2024

PGENETICS-D-24-00438R1 

Cell non-autonomous signaling through the conserved C. elegans glycoprotein hormone receptor FSHR-1 regulates cholinergic neurotransmission 

Dear Dr Kowalski, 

We are pleased to inform you that your manuscript entitled "Cell non-autonomous signaling through the conserved C. elegans glycoprotein hormone receptor FSHR-1 regulates cholinergic neurotransmission" has been formally accepted for publication in PLOS Genetics! Your manuscript is now with our production department and you will be notified of the publication date in due course.

The corresponding author will soon be receiving a typeset proof for review, to ensure errors have not been introduced during production. Please review the PDF proof of your manuscript carefully, as this is the last chance to correct any errors. Please note that major changes, or those which affect the scientific understanding of the work, will likely cause delays to the publication date of your manuscript. 

Soon after your final files are uploaded, unless you have opted out or your manuscript is a front-matter piece, the early version of your manuscript will be published online. The date of the early version will be your article's publication date. The final article will be published to the same URL, and all versions of the paper will be accessible to readers.

Thank you again for supporting PLOS Genetics and open-access publishing. We are looking forward to publishing your work! 

With kind regards,

Anita Estes

PLOS Genetics

On behalf of:

The PLOS Genetics Team

Carlyle House, Carlyle Road, Cambridge CB4 3DN | United Kingdom

plosgenetics@plos.org | +44 (0) 1223-442823

plosgenetics.org | Twitter: @PLOSGenetics
